# Targeting the Main Protease (M^pro^, nsp5) by Growth of Fragment Scaffolds Exploiting Structure-Based Methodologies

**DOI:** 10.1021/acschembio.3c00720

**Published:** 2024-01-17

**Authors:** Nadide Altincekic, Nathalie Jores, Frank Löhr, Christian Richter, Claus Ehrhardt, Marcel J. J. Blommers, Hannes Berg, Sare Öztürk, Santosh L. Gande, Verena Linhard, Julien Orts, Marie Jose Abi Saad, Matthias Bütikofer, Janina Kaderli, B. Göran Karlsson, Ulrika Brath, Mattias Hedenström, Gerhard Gröbner, Uwe H. Sauer, Anastassis Perrakis, Julian Langer, Lucia Banci, Francesca Cantini, Marco Fragai, Deborah Grifagni, Tatjana Barthel, Jan Wollenhaupt, Manfred S. Weiss, Angus Robertson, Adriaan Bax, Sridhar Sreeramulu, Harald Schwalbe

**Affiliations:** † Institute for Organic Chemistry and Chemical Biology, Goethe University Frankfurt am Main, D-60438 Frankfurt, Germany; ‡ Center of Biomolecular Magnetic Resonance (BMRZ), Goethe University Frankfurt am Main, D-60438 Frankfurt, Germany; § Institute of Biophysical Chemistry, Goethe University Frankfurt am Main, D-60438 Frankfurt, Germany; ∥ Department of Biochemistry, University of Zurich, 8093 Zurich, Switzerland; ⊥ SavernaTherapeutics, 4105 Biel-Benken, Switzerland; # Department of Pharmaceutical Sciences, University of Vienna, Josef-Holaubek-Platz 2, 1090 Vienna, Austria; ∇ Swiss Federal Institute of Technology, Laboratory of Physical Chemistry, ETH Zurich, 8093 Zürich, Switzerland; ○ Swedish NMR Centre, Department of Chemistry and Molecular Biology, University of Gothenburg, SE40530 Göteborg, Sweden; ◆ SciLifeLab, University of Gothenburg, SE40530 Göteborg, Sweden; ¶ Swedish NMR Centre, Department of Chemistry, University of Umeå, SE-90187 Umeå, Sweden; ⋈ Protein Production Sweden, Department of Chemistry, University of Umeå, SE-90187 Umeå, Sweden; ⧓ Oncode Institute and Division of Biochemistry, The Netherlands Cancer Institute, 1066CX Amsterdam, The Netherlands; ⧖ Max Planck Institute of Biophysics, D-60438 Frankfurt am Main, Germany; ● Magnetic Resonance Center and Department of Chemistry, University of Florence, Via L. Sacconi 6, 50019 Sesto Fiorentino, Italy; ¤ Consorzio Interuniversitario Risonanze Magnetiche Metalloproteine, Via L. Sacconi 6, 50019 Sesto Fiorentino, Italy; ☼ Macromolecular Crystallography, Helmholtz-Zentrum Berlin, Albert-Einstein-Str. 15, D-12489 Berlin, Germany; ◎ NIH, LCP NIDDK, Bethesda, Maryland 20892, United States

## Abstract

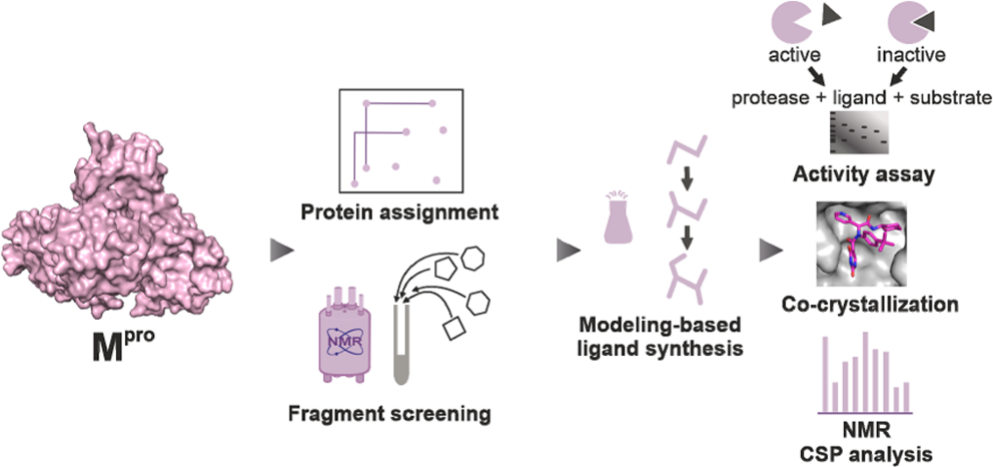

The main protease M^pro^, nsp5, of SARS-CoV-2 (SCoV2) is one of its most attractive drug targets. Here, we report primary screening data using nuclear magnetic resonance spectroscopy (NMR) of four different libraries and detailed follow-up synthesis on the promising uracil-containing fragment Z604 derived from these libraries. Z604 shows time-dependent binding. Its inhibitory effect is sensitive to reducing conditions. Starting with Z604, we synthesized and characterized 13 compounds designed by fragment growth strategies. Each compound was characterized by NMR and/or activity assays to investigate their interaction with M^pro^. These investigations resulted in the four-armed compound **35b** that binds directly to M^pro^. **35b** could be cocrystallized with M^pro^ revealing its noncovalent binding mode, which fills all four active site subpockets. Herein, we describe the NMR-derived fragment-to-hit pipeline and its application for the development of promising starting points for inhibitors of the main protease of SCoV2.

## Introduction

A global pandemic originated in late 2019 in Wuhan, China, and is caused by severe acute respiratory syndrome coronavirus 2 (SARS-CoV-2, SCoV2). The disease resulting from infection with SCoV2 is called coronavirus disease 2019 (Covid19). Covid19 led to nearly seven million deaths up to May 2023.^1^ SCoV2 shares similarities with previous outbreaks of SCoV in 2002 and the Middle East respiratory syndrome coronavirus (MERS-CoV) in 2012.^2^ The SCoV2 outbreak surpassed both its scope and severity due to its fast spread and pathology, resulting in a severe course of disease and eventually death.^3^ The positive-strand RNA virus with ∼30,000 nucleotides encodes for 14 nonstructural proteins (nsps), four structural proteins, and approximately 10 accessory factors.^4,5^ Besides vaccines that mainly focus on the viral entry mechanism targeting the viral structural protein Spike,^6−8^ development of small-molecule-based drugs is a major approach to combat the virus, especially after viral infection. Viral proteins have been shown to be validated targets, mainly those that are conserved from SCoV and to harbor crucial roles within the viral life cycle.^9^

One group of these proteins comprises the two proteases (nsp3d and nsp5) that post-translationally process the two polyprotein chains 1a and 1ab to release the 14 individual nsps. nsp5 is known as the main protease (M^pro^). It is a 3C-lile protease (3CLPro), possesses a chymotrypsin-like fold and processes 11 out of 14 cleavage sites, including its own release,^10^ whereas nsp3d, the papain-like protease (PL^Pro^), processes the remaining three cleavage sites.^11^ Besides the RNA polymerase nsp12 with mainly nucleoside analogues as inhibitors, either approved or in clinical trials,^12−14^ M^pro^ is the major drug target of the drug PF-07321332 (Nirmatrelvir) developed by Pfizer,^15^ which was conditionally approved by the EMA and FDA. The highly sequentially and structurally conserved cysteine protease M^pro^ is a 67.6 kDa homodimer with an active site catalytic dyad His41-Cys145. The protein monomers are arranged almost perpendicular to one another, and both C- and N-termini are involved in the dimer interface. The active site consists of four subpockets S1′, S1, S2, and S3 with the catalytic dyad located in subpocket S2. The second monomer is crucial in the formation of the S1 subpocket of the first monomer, underlining the importance of dimerization for the catalytic activity. Strikingly, M^pro^ requires the amino acid glutamine on the N-terminal cleavage site of the substrate. This sequence requirement has not been observed for any human protease, rendering M^pro^ a promising viral drug target with the potential for low off-target effects.^10^ Further, M^pro^ does not show any mutation from the original SARS-CoV-2 in alpha, gamma, and delta variants and only one point mutation in the beta (K90R) and omicron (P132H) variants, while, e.g. the omicron spike protein has more than 30 mutations.^16^

Both the necessity of dimerization and the availability of the substrate-binding pocket for M^pro^ activity allow the design of compounds that block the activity by masking the dimer interface that block the substrate-binding pocket, or that allosterically reshape.

Both noncovalent and covalent binders have been reported. Since M^pro^’s active site requires glutamine to be part of the recognized substrate polypeptide chain, several peptidomimetics were designed that use noncleavable cyclic glutamine analogues mimicking the substrate’s binding mode.^17,18^ One such molecule is Nirmatrelvir, which has a nitrile group as the reactive warhead allowing reversible covalent binding to the active site Cys145.^15^ Furthermore, noncovalent high-affinity binders such as *(R)*-X77 that fill all four subpockets can be derivatized to form covalent bonds by replacement of the imidazole moiety that is near the active site Cys145 by warheads.^19,20^

Repurposing of drugs is commonly utilized to accelerate the approval phase.^21^ A different approach is to start with fragment molecules that serve as molecular scaffolds and to grow or link one or several fragments to improve binding affinity and specificity.^22,23^ In both cases, experimental structural data and/or modeling are used to prioritize initial screening hits for subsequent medicinal chemistry optimization cycles. Also AI methodolgy has very recently been proposed to expedite the process.^70^

Crystallography (X-ray) and nuclear magnetic resonance spectroscopy (NMR)^24−28^ have been used as primary structural screens for M^pro^. In addition, activity-based readouts have been performed.^29,30^ Soon after the outbreak of Covid19, based on the previously determined SCoV M^pro^ structure,^31^ the structure of the SCoV2 homologue was solved, thus establishing crystallization conditions for fragment soaking into crystals.^10^ For NMR, optimized expression and buffer conditions were reported.^32^

One of the campaigns initiated from X-ray fragment screening that used the DSI-poised library helped launch the COVID Moonshot open-science initiative aiming toward developing antivirals.^25,33,34^ Within the Covid19-NMR consortium, complementary NMR-screening campaigns used the same library to screen 20 RNA elements and 25 viral proteins under identical conditions.^27,28^

**Figure 1:**
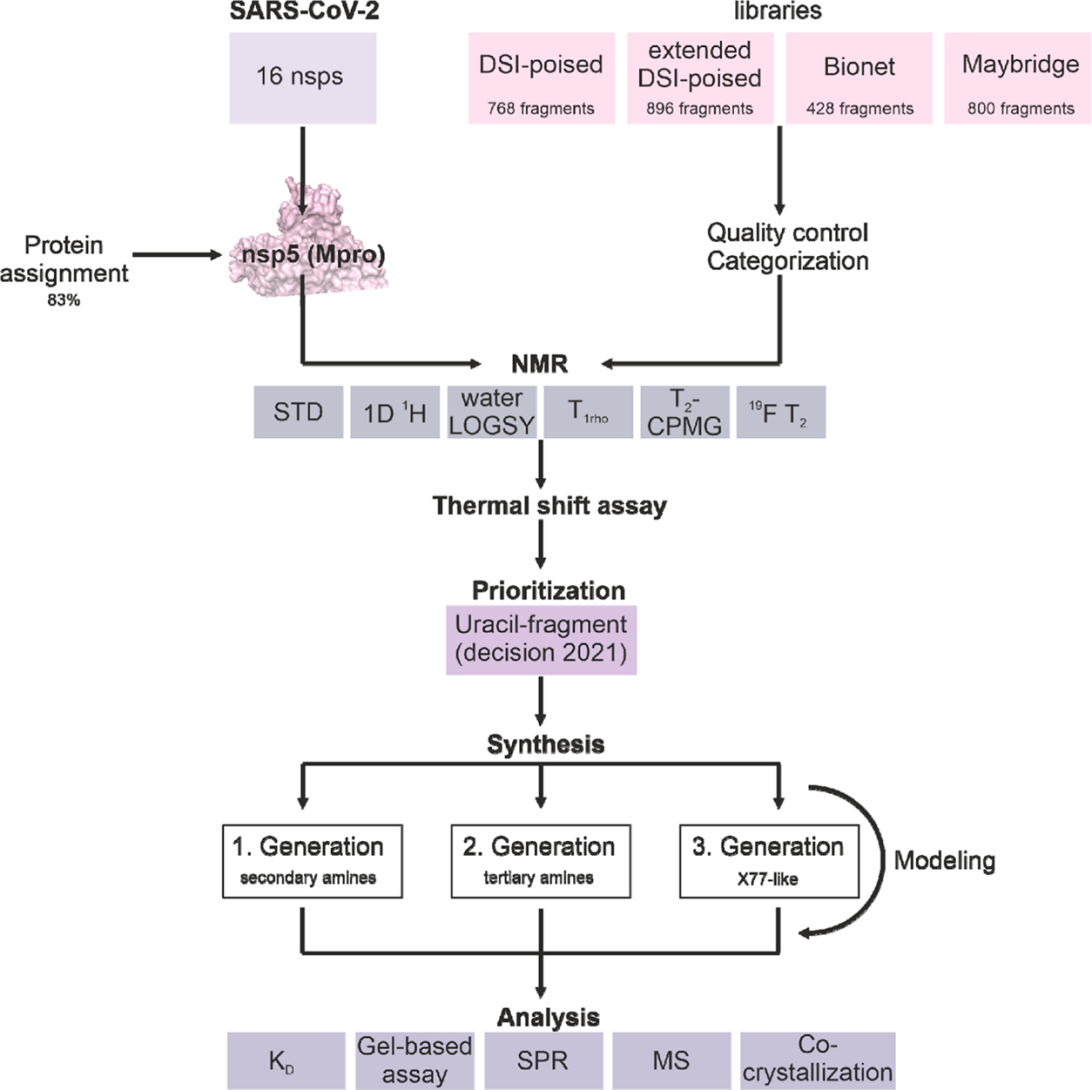
Workflow showing all components of this approach from fragment screening and protein assignment through ligand development finally to a fragment containing the X77-like M^pro^ inhibitor.

**Figure 2:**
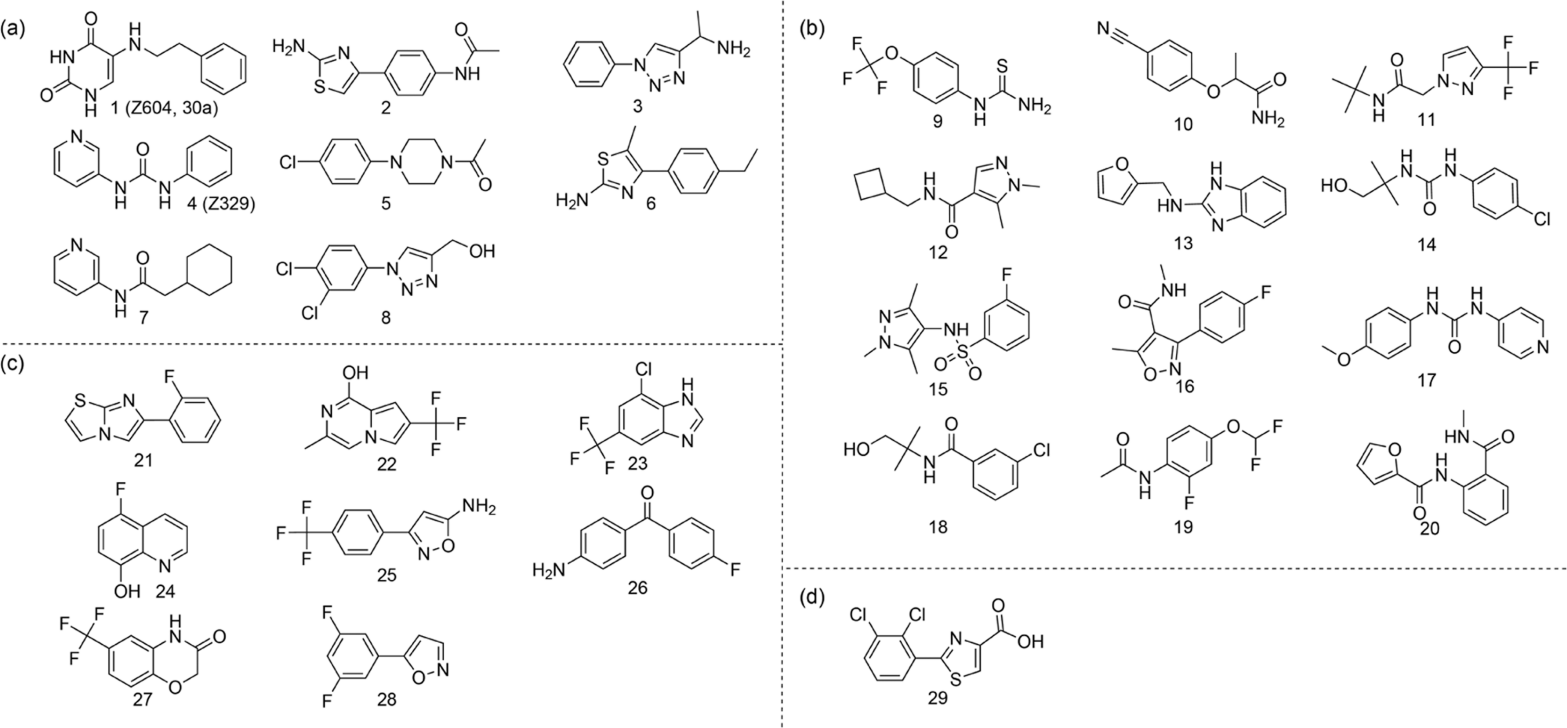
Summary of prioritized binders for M^pro^. Hits from (a) DSI-poised, (b) extended DSI-poised, (c) Bionet, and (d) the Maybridge libraries.

Here, we report the follow-up of these initial NMR-based screenings using four fragment libraries with 768/896/428/800 compounds in total screening three previously established M^pro^ constructs resulting in 217 hits for M^pro^, 112 hits for _GS_nsp5_GPH6_, and 38 hits for _GHM_nsp5. These screens were conducted in three different NMR laboratories. Twenty-nine hits of these were pursued to conform their binding by additional methods such as their in vitro inhibitory effect. One compound (Z604, Figure 2) derived from NMR screens was prioritized based on computational modeling. A series of derivatives were synthesized to decorate the molecule and improve its characteristics including stability, solubility, and binding. We here document the iterative workflow implementing NMR screening, additional biophysical screens, computational docking, medicinal chemistry, and biological assays (Figure 1).

## Results and Discussion

### Primary Screening Hits

M^pro^ is the most highly prioritized viral target protein. Here, we report on efforts to investigate the main protease. These efforts were conducted in several NMR groups at the same time. As a consequence of this interlaboratory project, different protein constructs, libraries, screening experiments, and buffer conditions were used. Thus, our report allows validating the outcome of the different approaches pursued. In addition to the coordinated NMR screening within the Covid19-NMR network, the group of Hanoulle screened wild-type (wt) M^pro^ independently and reported their results in 2021.^35^

Within the Covid19-NMR consortium, we screened four libraries with NMR (Table 1). One of these libraries, the DSI-poised library, was screened against M^pro^ not only by NMR but also by X-ray. Conducting structural screens using different libraries and two independent methods allows us to compare interoperability between different NMR screens, assess hit-rates over four different libraries, optimize NMR data acquisition and analysis, broaden an initial SAR profile of over 1000 fragments derived from four different libraries, and compare the results from NMR and X-ray. The DSI-poised library was also used to screen different constructs of M^pro^, showing that the screening results are sensitive to the exact protein sequence and the associated monomer–dimer ratio.^28^

**Table 1:** Summary of Primary Screening Performed at Three Different Sites with Four Libraries

library	DSI-poised	extended DSI-poised	bionet	Maybridge
compound quantity	768	896	428	800
group	Schwalbe et al.	Orts et al.	Karlsson et al.	Karlsson et al.
performed NMR experiments	1D ^1^H	STD	^19^F T_2_	T_2_-CPMG
	STD		T_2_-CPMG	STD
	waterLOGSY		T_1rho_	
	T_2_-CPMG		STD	
NMR spectrometer	600 MHz, cryoprobe	600 MHz, cryoprobe	600 and 700 MHz, cryoprobe (sites 1 and 2)	600 MHz, cryoprobe
sample condition	10 μM _GHM_nsp5, 200 μM of each ligand in 25 mM NaPi (pH 7.5), 150 mM NaCl, 5% *d*_6_-DMSO.	11.5 μM _GS_nsp5_GPH6_, 320 μM of each ligand in 10 mM NaPi (pH 7.6), 50 mM NaCl in D_2_O, 0.04% NaN_3_, 3.85% *d*_6_-DMSO.	10 μM wt M^pro^, 100 μM of each ligand in 10 mM NaPi (pH 7.4), 130 mM NaCl, 2.4 mM KCl, 10% D_2_O, 8% *d*_6_-DMSO.	10 μM wt M^pro^, 100 μM of each ligand in 10 mM NaPi (pH 7.4), 130 mM NaCl, 2.4 mM KCl, 10% D_2_O, 1% *d*_6_-DMSO.
ligand mixture	12 ligands per mixture, 64 mixtures in total	max. six ligands per mixture	9–11 ligands per mixture	10 ligands per mixture
hit definition	satisfaction of ≥2 criteria	STD signal	binding response in ≥5 experiments:	
	CSP	S/*N* > 10	T_2_ (site 1)	
	line broadening		STD (site 1)	
	sign change in the waterLOGSY		^19^F T_2_ (site 1)	Binding response in both experiments:
	STD signal		T_1rho_ (site 2)	T_2_
	Signal intensity decrease in T_2_-CPMG		STD (site 2)	STD
			^19^F T_2_ (site 2)	
additional experiments	X-ray		SPR	SPR
	SPR			
	TSA			

Interestingly, the comparison of screening hits reveals only a small overlap of the screening results. This is due to several factors: (1) differences in the exact library composition; (2) differences in the NMR experiments, analysis, and thresholding for binding; and (3) differences in the protein constructs despite almost identical crystallographic structures.

In the Orts group, the _GS_nsp5_GPH6_ construct (SI methods) was screened against the extended DSI-poised library containing 896 compounds using saturation transfer difference (STD) NMR experiments, and 104 hits were identified and analyzed for structural and chemical similarities (IDs **9**–**20**, **796**–**1679**, Table S1 and Figure S1). Even using the identical protein construct but slight differences in buffer conditions (Table 1), work conducted in the Schwalbe group identified only four overlapping binders when applying additional ligand-based NMR experiments and more stringent hit criteria, suggesting that STD experiments are the most sensitive to detect even weak binding. From this screening, based on three-dimensional (3D) pharmacophore analysis, 12 hits were recommended for follow-up studies (Figure 2(b)). The Bionet library with 428 fragments and the Maybridge library with 800 fragments were screened by the Swedish NMR Centre (SNC) (Karlsson group) against wt M^pro^ using several NMR experiments (including ^19^F-specific NMR in addition to ^1^H NMR experiments). The ^19^F-labeled Bionet library was screened at two different sites within SNC in parallel to further validate the results, resulting in 113 hits in the Bionet library (Table S1, IDs **21**–**28**, **1680**–**2099**, Figure S1) and 26 hits in the Maybridge library (Table S1, IDs **29**, **2100**–**2758**, and Figure S1). Eight hits from the Bionet library and one from the Maybridge library were selected for further studies by surface plasmon resonance (SPR) and chemical shift perturbation (CSP) experiments as binding could be detected in at least five out of six experiments recorded for the Bionet fragments and both experiments recorded for the Maybridge fragments (Figure 2(c,d)).

In the Schwalbe group, hits from the DSI-poised library were screened over three different constructs to prioritize for follow-up medicinal chemistry, as reported below. Using the _GHM_nsp5 construct (SI methods) resulted in 38 initial hits (Table S1, IDs **1**–**8**, **36**–**795**, Figure S1). From these hits, four have cocrystal structures with wt M^pro^. The NMR hits were followed up by thermal shift assays (TSAs) that narrowed down the number of hits to 18. With these, we performed SPR spectroscopy showing responses for 8 hits (Figure 2(a)). Two of these hits were already cocrystallized.

To characterize the binding site, we performed two-dimensional (2D) [^1^H,^15^N]-NMR experiments using _GHM_nsp5. In parallel, we had also established the expression and purification of _GS_nsp5 (SI methods) and wt M^pro^. The two proteins have different tendencies to dimerize,^32^ revealing that at primary screening conditions both _GHM_nsp5 and _GS_nsp5 are mainly monomeric. As a consequence, we repeated the experiments using the _GS_nsp5 and the dimeric wt for the five most promising of eight hits. Two hits, Z57744604 (Z604) and Z44592329 (Z329), showed binding to the wt protein in 2D HSQC experiments. Binding of Z604 is detected only by NMR (Figure S2), while Z329 is detected as a hit by both NMR and X-ray.

In general, most of the binders consist of the two common (aromatic) rings that possibly fit into two of the four subpockets of the active site. Differences in the substituents of aromatic or saturated 5- or 6-membered rings likely discriminate between the subpockets depending on the available functional groups of the ligands. Several compounds show amide moieties that mimic the native substrate. Further, several of the ^19^F-containing compounds harbor a trifluoromethyl moiety known from Nirmatrelvir. The ligands of the extended DSI-poised library show the highest druglikeliness on average (Figure S3). The lipophilicity is the highest for hits from the Bionet and Maybridge libraries, which in turn is reflected in the lower water solubility of hits derived from those two libraries compared to the DSI-poised library hits. Water solubility is an important property of bioavailability. In addition, the ligands of the first two libraries have a higher number of H-acceptors and H-donors, conferring binding specificity. The libraries used have been proven to represent chemical diversity.^36^

### Mapping of Ligand Binding Epitope

To determine epitopes for ligand binding and to map chemical shift perturbations onto the published M^pro^ structures, the availability of NMR chemical shift assignment of the wt protein is crucial. Previous work yielded a wt backbone resonance assignment to an extent of 63% (BMRB: 50780),^35^ and the nearly complete (>97%) assignment of the active site mutant C145A without (BMRB: 51455) and with its native substrate peptide (BMRB: 51456).^37^ This C145A mutant was reported to stabilize the dimer state resulting in an increase in protein stability allowing extensive multidimensional NMR experiments.^38^ In fact, we observe that the spectrum of wt M^pro^ changes over time starting after one day of measurement at 500 μM concentration and a temperature of 298 K. Under these conditions, a second set of signals appears arising from the protein monomer. We assign this newly appearing second set of NMR resonances to a partially proteolyzed monomer. This assignment is supported by the comparison of the chemical shift pattern of wt vs monomeric mutant (_GS_nsp5 and _GHM_nsp5) and SEC-MALS data.^32^

Using multiple uniformly ^2^H-, ^3^C-,^15^N-labeled samples, we were able to assign 83% of the wt backbone signals (Figure S6). Most of the amino acids, which are part of the substrate-binding pocket, are assigned, except for residues of the catalytic dyad.

With the assignment in hand, we were able to determine the affected amino acids by detection of CSPs and by mapping these CSPs onto the crystal structure, allowing the detection of binding sites and affinities of the various compounds.

For the purpose of demonstrating the binding, we used known noncovalent binders: Z329,^39^ which is also a hit in our primary screening; *(R)*-X77, which is a nanomolar binder;^20^ the irreversible covalent binder Carmofur;^40,41^ and the noncovalent binder Mcule-5948770040 (M040)^42^ (Figure 2(b,c), Table S2 and Figure S7), all of which have published cocrystal structures with M^pro^ as positive controls.

**Figure 3:**
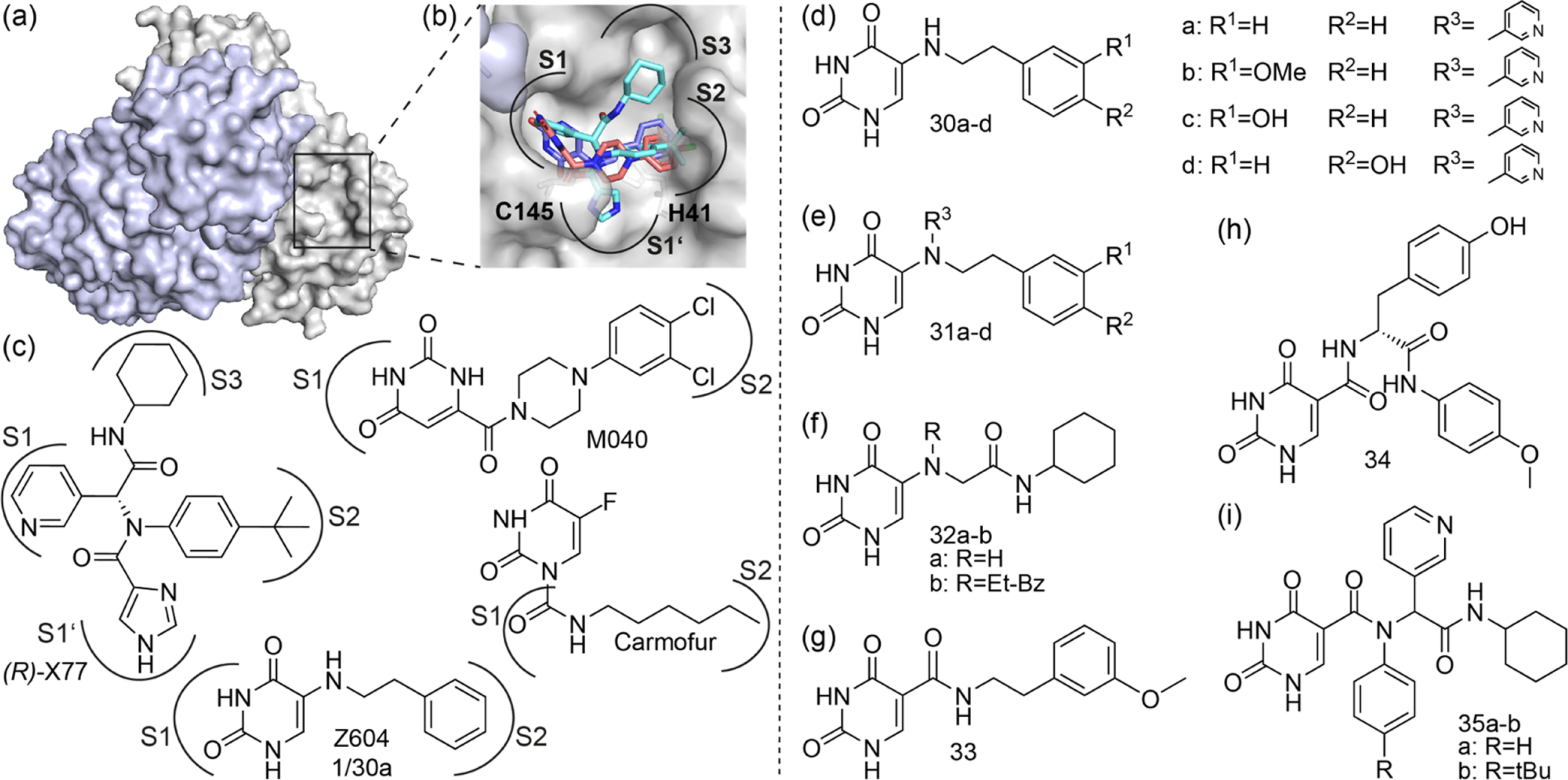
M^pro^ dimer visualized in surface representation (a), with monomers in gray and light purple (PDB: 6Y2E), and a detail of the active site pocket (b), as circled in (a). The active site dyad, modeled Z604 (purple), cocrystallized *(R)*-X77 (cyan), and M040 (red) are shown in stick representation. Molecular structures of reference molecules *(R)*-X77, M040, and Z604 (c) and their derivatives (d–i). Secondary amines **30a**–**d** (d), their corresponding tertiary amines **31a**–**d** (e), derivatives based on **30b** or **30d** involving amide bonds **32**–**34** (f–h), and four-armed derivatives with amide bonds (**35a**–**b**), which are hybrids of Z604 and *(R)*-X77 (i). The subpockets are indicated for the cocrystallized compounds and for Z604 from our modeling data.

One ligand that was defined as a hit in the initial screenings and that stands out in protein-detected NMR experiments is Z604 (Figure 3(c) and Table S2). This compound harbors a uracil moiety also found in Carmofur and M040, confirming the uracil scaffold as a lead. In the case of Carmofur, this nucleobase moiety acts as a leaving group to promote covalent binding of its substituent, whereas in the case of M040, the uracil moiety binds in the subpocket S1. The uracil moiety seems to be able to interact with the subpocket S1 and thus helps in the localization of Carmofur prior to the covalent bond formation.

Z604 features characteristics of a reversible covalent binder: it is sensitive to reducing agents (Figure S4) and reveals effects that depend on incubation time (Figure S8). In the presence of dithiothreitol (DTT), the ligand does not bind to M^pro^, but as soon as DTT loses its reducing activity, binding is driven to saturation. Then, addition of a reducing agent has no effect on the NMR CSPs of the already bound state. Z604 ages with time to convert into several reaction products. In DMSO at room temperature, this degradation reaches >90% only after ∼2 months. The reaction product is unstable but is more rapidly formed in an aqueous buffer and can no longer be detected by NMR after several hours (Figure S5).

Interestingly, Z604 binds M^pro^ in a time-dependent manner (∼10 h). This time-dependent binding is in agreement with the reversible covalent binding behavior of Z604. We speculate that Z604’s reactivity could be linked to a nucleophilic attack of a free sulfhydryl group of the cysteine side chain forming a carbon–sulfur bond (Figure 3(c)).^43^ Such reactivity is known from the methylation of uracil to form thymine by the enzyme thymidylate synthase.^44^

The covalent binding is further supported by NMR competition experiments with *(R)*-X77. If *(R)*-X77 is added first to the protein, Z604 is not able to outcompete the nanomolar binder. If, however, Z604 is added first, *(R)*-X77 is not able to outcompete Z604.

### Modeling and Synthetic Ligand Growth

Despite several attempts, we were not successful in cocrystallizing the Z604-M^pro^ complex. Therefore, we modeled Z604 into the active site of M^pro^ based on known ligand binding modes of similar moieties (Figure 5(c,e)).^42^ The molecular graphics program Wit!P^45^ was used to build possible protein/ligand complexes manually. CHARMM22^46^ was used to assign atom types and to conduct energy minimization calculations. The model provided insights into which groups in Z604 are important for binding and where in the molecule changes could be made to improve binding affinity (Figure 3(d–i)).

Compounds **30a**–**d** (Figure 3(d)) feature secondary amines linking the uracil moiety to an alkyl chain with differently substituted phenyl rings. We thus synthesized derivatives containing various groups at the phenyl moiety like hydroxyl/methoxy in different substitution patterns through a nucleophilic amination described by Phillips et al. (Scheme S1).^47^

The second-generation compounds **31a**–**d** are tertiary amines for which we utilized *N*-alkylation of the corresponding synthesized secondary amines with bromomethyl-pyridine in the presence of DIPEA (Scheme S1).^48^

For further derivatives, the aim was to generate peptide-like linkages that resemble substructural moieties of the already known nanomolar binder *(R)*-X77 (Figure 3(b,c)). For this purpose, derivatives **32a–b, 33**, and **34** (Figure 3(f–h)) were synthesized with a classical carbodiimide approach following *N*-alkylation in the cases of **32b** and **34** (Schemes S2–S4).

Finally, analyzing the substituents promoting binding and inhibition from the first ligand series, we realized the utility of the *Ugi* reaction to assemble ligands that potentially bind to all four subpockets in a single reaction step. That method was previously established by other groups.^49,50^ Using this four-component one-pot *Ugi* condensation,^51^ we were able to synthesize X77-like molecules **35a**–**b** by changing the imidazole of the X77 to the uracil moiety (Scheme S5).

### Crystallization

For all synthesized ligands, several cocrystallization conditions were tested without yielding any cocrystals. However, soaking resulted in a cocrystal of **35b** (Figure 5(d)). As predicted from our modeling data, the uracil moiety of **35b** occupies the subpocket S1’ as the imidazole group of *(R)*-X77. This is in contrast to the uracil moiety occupying the S1 subpocket in the case of M040 (as well as in our modeling of Z604). The pyridine group is in the subpocket S1 as for *(R)*-X77, but both the *tert*-butylbenzene and the cyclohexane moieties are twisted in comparison to *(R)*-X77. In addition, the **35b** cocrystal structure revealed a second binding site for **35b** remote from the active site (Figure S13). This was also verified via CSPs (Figure 5(a)). However, the second binding site is remote from the active site.

**Figure 4:**
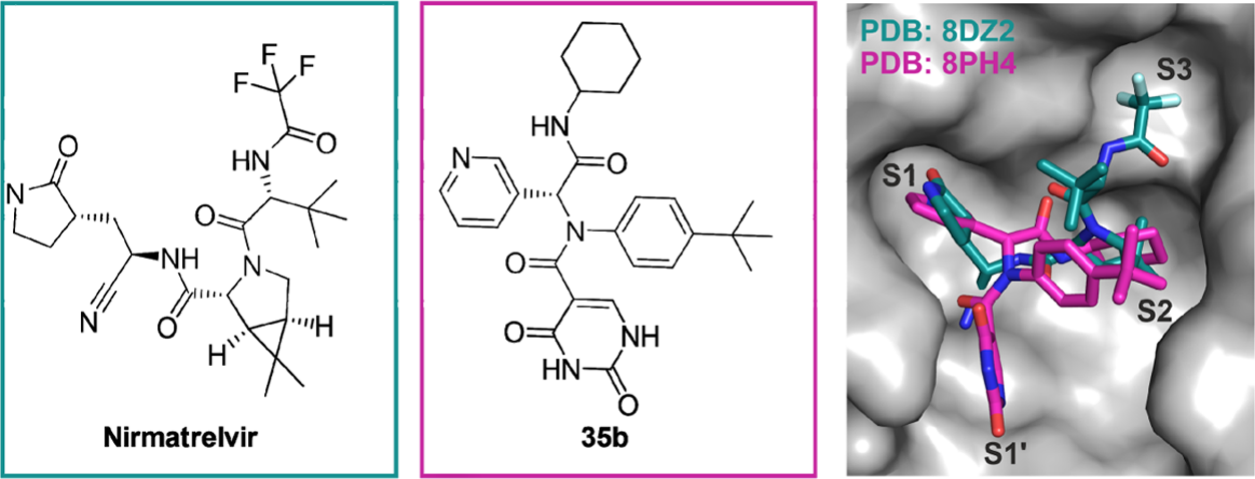
Comparison of the crystal structures of Nirmatrelvir (PDB: 8DZ2) and **35b** (PDB: 8PH4).

A comparison of synthesized **35b** with the already known Mpro drug Nirmatrelvir (reversible covalent binding) shows that there are differences in their orientation in the binding pockets. While Nirmatrelvir enters deep into subpocket S3, our lead compound **35b** is able to fill the subpocket S1’(Figure 4).

**Figure 5:**
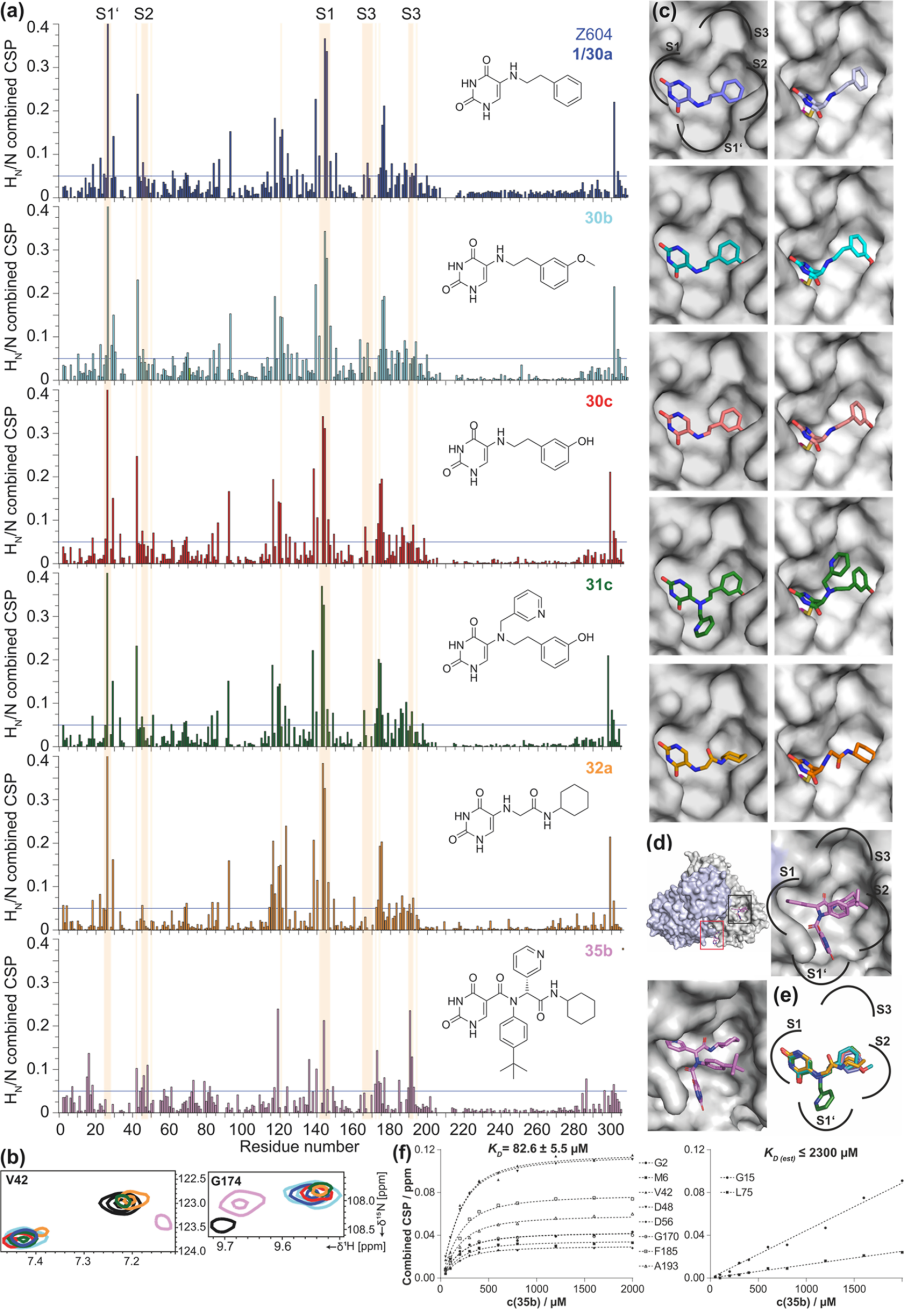
CSP plots of Z604 (purple), **30b** (light blue), **30c** (red), **31c** (green), **32a** (orange), and **35b** (pink) (a). The light-pink shade shows the active site amino acids with the indication of the subpockets (exemplary amino acids for S1’: T24, T25, T26, L27, S1: F140, L141, N142, G143, S144, C145, H163, H164, S2: C44, T45, S46, E47, M49, S3: L167, P168, G170, T190, Q192). Exemplary CSPs of V42 and G174 in the absence (black) and presence of ligands are shown in (b). See also Figures S9–S10 for the reference compounds. Modeling of each ligand within the active site pocket of M^pro^ ((c) left column: noncovalent; right column: covalent binding). Amino acids N142 and Q189 are removed for visibility of the active site pockets (see Figure S11 for the same structures with all amino acids). The cocrystal structure of M^pro^ with two **35b** binding sites (PDB: 8PH4) shown as a dimer with indication of first (black rectangle) and second binding sites (red). A section of the active site is shown in the same position as in (c) and when tilted to show all moieties within the pocket (d). Overlay of all modeled ligands with the indication of subpockets (e). *K*_D_ determination derived from combined CSPs for the first (left) and second binding sites of **35b** (right) (f).

### Activity and NMR Interaction Data

**Figure 6:**
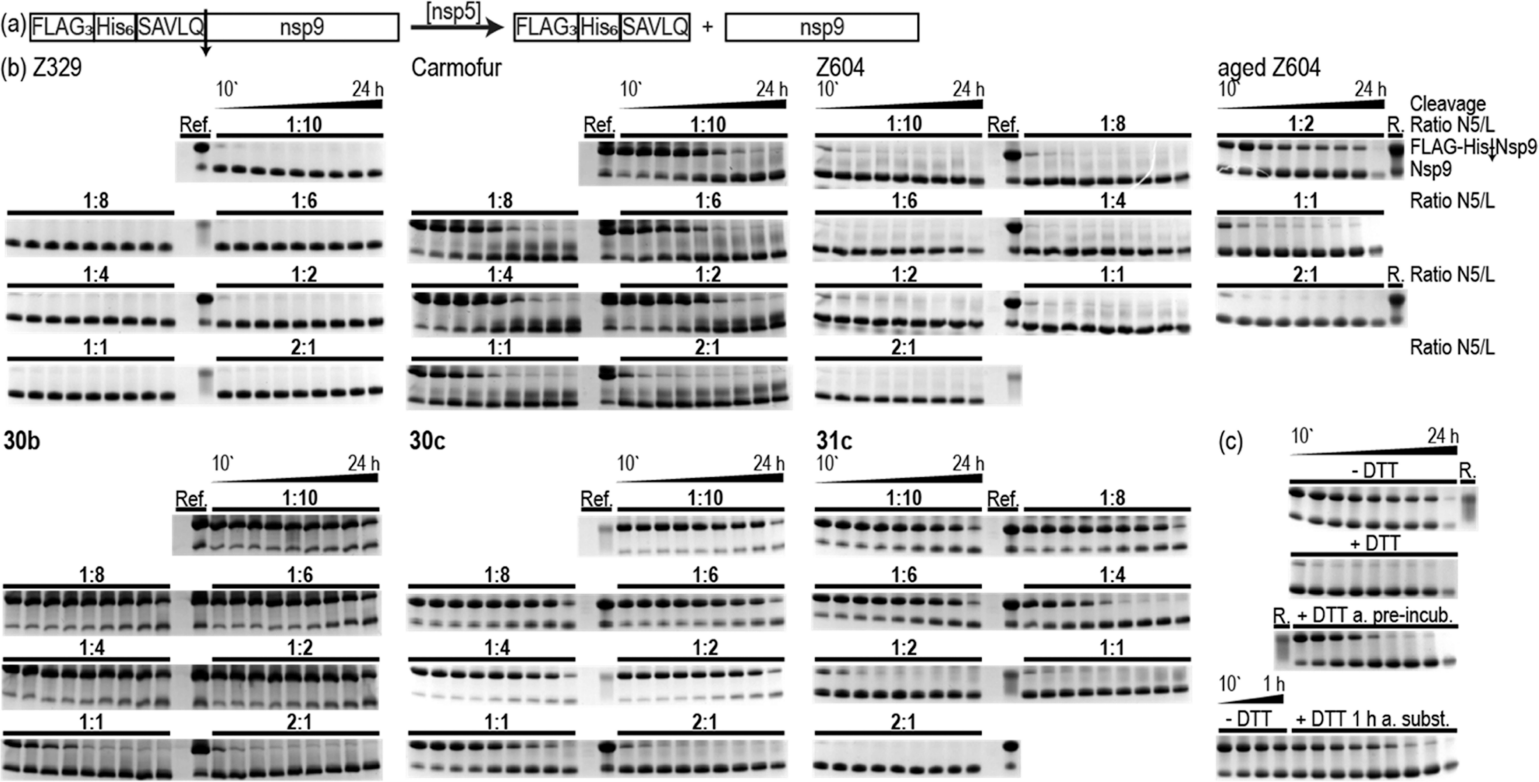
(a) Schematic representation of the enzymatic cleavage reaction performed. The substrate FLAG_3_-His_6_-SAVLQ-nsp9 is cleaved by M^pro^, resulting in the FLAG_3_-His_6_-SAVLQ peptide and the native nsp9. (b) Concentration-dependent activity assay of M^pro^ in the presence of ligands **30b**, **30c**, **and 31c** and the reference compounds Z604, aged Z604, Z329, and Carmofur. 200 μM M^pro^ was preincubated at 22 °C for 24 h. Ligand concentrations during preincubation are 0.1, 0.2, 0.4, 0.8, 1.2, 1.6, and 2 mM (diluted to half the concentration for cleavage). DTT dependence on inhibition of aged Z604 (c), without DTT (-DTT), with DTT during both preincubation and cleavage (+DTT), addition of DTT after the preincubation but before substrate addition (+DTT a. preincub.), and addition of DTT 1 h after substrate addition (+DTT 1 h a. subst.). The substrate concentration is 275 μM for fresh and aged Z604, **30c**, **31c**, Z329, and 296 μM for **30b** and Carmofur. The cleavage reaction is stopped after 10', 20', 40', 1, 2, 4, 6, 8, and 24 h and is indicated in the figure by a bar with the starting and endpoints. The ratio N5/L indicates the ratio of M^pro^ to ligand; for DTT dependence, the ratio N5/L is 1:2. ref/R. (reference) lanes include M^pro^ (cut from gel), and FLAG_3_-His_6_-SAVLQ-nsp9. Substrate (FLAG-His-nsp9) and cleaved nsp9 migration levels are indicated at the right of the gels.

Next to binding, we tested the inhibition of the M^pro^ cleavage activity. A sodium dodecyl-sulfate polyacrylamide gel electrophoresis (SDS-PAGE)-based activity assay described previously^30^ (Figure 6(a)) was performed under similar conditions as our NMR experiments with all 18 above-mentioned compounds (M^pro^/ligand 1:10). We preincubated M^pro^ with the ligand for either one or 24 h prior to addition of the substrate FLAG_3_-His_6_-SAVLQ-nsp9. Since Z604 is sensitive to reducing conditions, all experiments were carried out in the absence of a reducing agent. The presence of the reducing agents TCEP/DTT, DMSO (solvent of all compounds), and the preincubation time had no significant effect on the activity of M^pro^ (Figure S13(a)). Other oxidizing agents, such as oxygen, were not considered.

Both Carmofur and *(R)*-X77 show similar results and no cleavage up to 5 h independent of preincubation (Figure S13(b)). M040 with a *K*_D_ of 1.3 ± 0.18 μM^42^ shows reduced activity but is less effective than Carmofur and *(R)*-X77. Z329 has been reported to feature a *K*_D_ of 461 μM.^28^ Thus, M040, a reported weak inhibitor, shows full cleavage after less than 1 h with no effect of preincubation time (Figure S13(b)). Freshly synthesized Z604 shows no effect after 1 h of preincubation but an effect comparable to Z329 after 24 h of preincubation. Thus, in line with our NMR data, the inhibitory effect is preincubation-time-dependent. Strikingly, if we use aged Z604, this changes drastically. In line with our NMR data, the inhibitory effect is preincubation-time-independent, and the inhibitory effect is then comparable to *(R)*-X77 and Carmofur (Figure S13(d)).

The three first-generation ligands (**30b**–**d**) show preincubation-time-dependent inhibition as the original compound Z604 (Figure S13(c)). The introduction of a hydroxyl or methoxy group (**30b**, **30c**) leads to an increase in stability and inhibitory effect, rendering these promising scaffolds/hits. The second-generation ligands (**31a**–**d**), however, show differences. **31a** and **31b** show no inhibition, in line with NMR data showing no significant changes in the NMR spectra. **31c** and **31d**, in contrast, show time-dependent inhibition. **31d** is less effective than the secondary amines, showing full cleavage after 1 h. **31c**, which only differs by the position of the hydroxyl group from **31d**, shows comparable results as the corresponding secondary amines (**30c**–**d**). These results are in line with fluorescence data and MALDI-MS data using the peptide AVLQSGFRKK as the ligand, however, using different buffers and concentrations of protein, substrate, and ligand.^52,53^ The third-generation compounds (**32**–**34**) containing amides show comparable inhibition as for the secondary amines in the case of **32a** (Figure S13(b)), weak inhibition in the cases of **35a** and **35b**, and no effect for **33** and **34**. These results are in line with the NMR experiments of **33** and **34**, showing no significant changes after compound addition (NMR data on **32b** and **35a**, not measured), and CSPs in the presence of **32a** and **35b**. Interestingly, the CSPs of all Z604 derivatives show a similar pattern, hinting at a similar binding mode, while the binding of **35b** seems to differ (Figure 5(a) and Table S2). By titration of **35b** to a protein sample and extraction of CSPs, a *K*_D_ constant of 82.6 ± 5.5 μM was determined for the first binding site (active site) and an estimation of ≤2.3 mM for the second (allosteric) binding site (Figure 5(f)).^54^

To further characterize and differentiate the ligands, we performed concentration-dependent activity experiments with an enzyme/ligand ratio of 2:1 up to 1:10 after 24 h preincubation of M^pro^ with the most promising ligands **30b**, **30c**, and **31c**, as well as freshly synthesized Z604, the aged Z604, Carmofur, and Z329 as references (Figure 6). Z604 and Z329 both show very weak inhibition at 1:10, and Carmofur shows partial inactivation at 1:1 but never fully inhibits the reactivity of M^pro^ under the conditions tested here. Aged Z604 shows comparably partial inactivation at 1:1 and nearly full inhibition at 1:2. The secondary amines **30b** and **30c** show comparable inhibition at an enzyme/ligand ratio of 1:1 but are fully preventing M^pro^ activity at 1:2. The effect of **30c** with the hydroxyl group seems to be slightly better. In the case of the tertiary amine **31c**, weak inhibition is visible at 1:2 and full inhibition is reached at 1:6, showing that the pyridine moiety decreases ligand activity.

To characterize the extent of reversible versus irreversible binding mode, we investigated the DTT presence with aged Z604 during the activity assay (Figure 6(c)).^55−57^ If DTT is absent, the activity is nearly fully inhibited with a 2-fold excess of ligand. The presence of DTT during both preincubation and cleavage leads to no inhibition. If DTT is added after the preincubation but before substrate addition, a regain of function is obtained. This is also the case if DTT is added 1 h after substrate addition. Although we have not proven reversible covalent binding, our data strongly suggest that there is no irreversible covalent bond.

## Conclusions

In conclusion, we report here a systematic approach to develop hits from initial fragments for which binding was detected by NMR structural screens. Our comparative analysis of four different screening campaigns shows that initial screening results are strongly dependent on exact buffer conditions and threshold settings to classify binding. These results strongly necessitate follow-up validation experiments, therefore we conducted a thermal shift assay and SPR experiments to prioritize from a large set of initial binding fragments.

We have designed and conducted follow-up medicinal chemistry experiments to improve the affinity and activity of compounds derivatized from a single initially prioritized ligand. From these experiments, we can derive a structure–function relation of the developed hits. During the development of new compounds, analysis of binding and activity showed that a large number of new compounds can be synthesized using one-pot *Ugi* condensation chemistry, which allows a versatile combinatorial synthesis for new hits and leads.

With new and reference compounds, we could derive binding epitopes for binding by NMR spectroscopy for all hits and by X-ray crystallography for one selected compound. For **35b**, we consistently find a second remote binding site by X-ray and NMR with very different binding affinities of ∼80 μM and ∼2 mM. This finding is interesting for exploring dual-inhibitor strategies for further medicinal chemistry campaigns.

## Methods

### Protein

The expression and purification of M^pro^ constructs have been described elsewhere.^32^ FLAG_3_-His_6_-SAVLQ-nsp9 expression has been described elsewhere.^30^ The Sf9 insect cells were lysed by microfluidization in 30 mM HEPES (pH 7.6), 250 mM sodium chloride, 5 mM magnesium acetate, 10% glycerol, 0.02% Triton X-100, 5 mM imidazole, and 10 mM β-mercaptoethanol. The cleared cell lysate was purified by Ni^2+^-affinity chromatography by use of the above-mentioned buffer with 500 mM imidazole. This was followed by size-exclusion chromatography (HiLoad 16/60 Superdex 75 pg (GE Healthcare)) in 25 mM sodium phosphate buffer (pH 7.5), 150 mM NaCl, and 2 mM TCEP-HCl.

### Primary Screening

See Table 1 for a summary of screening data collection and the SI Methods section for detailed protocols.

### Resonance Assignment

Experiments for protein resonance assignment employed a 530 μM uniformly ^2^H, ^13^C, ^15^N-labeled nsp5 sample in 25 mM sodium phosphate buffer (pH 7.0), containing 2 mM TCEP, 5% D_2_O, and 0.15 mM DSS as the internal chemical shift reference. BEST-TROSY versions of 3D HNCO, HNCA, HNCACB, and HN(CO)CACB experiments^58,59^ were acquired at a sample temperature of 298 K on a Bruker AvIIIHD spectrometer with a ^1^H frequency of 800.13 MHz equipped with a 5 mm TCI ^1^H[^13^C/^15^N] cryogenic probe. Comparison with the published assignment of the active site mutant (BMRB: 51455) helped in cases with some degree of uncertainty due to the monomeric protein, giving rise to a second signal set, which gets more populated with time. Data analysis and assignment were done in Bruker TopSpin 4.0.9 and Sparky 3.114.^60^

### Ligand Interaction and Mapping

For protein-detected ligand interactions, we recorded 2D [^1^H, ^15^N]-NMR experiments with 100–200 μM uniformly ^15^N-labeled M^pro^ with 10-fold excess for Z604 and its derivatives, 1.5-fold for *(R)*-X77, and 2-fold for Carmofur, and corresponding reference spectra without ligands with the same amount of *d*_6_-DMSO. For CSP analysis and time-resolved experiments, we used [^1^H, ^15^N]-BEST-TROSY and [^1^H, ^15^N]-SOFAST-HMQC pulse sequences, respectively.^61,62^ Dissociation constants were determined as described elsewhere by plotting the largest CSP against the ligand concentration and fitting with the following equation: Δδ_obs_ = Δδ_max_{([*P*]_*t*_ + [*L*]_*t*_ + *K*_d_)-[([*P*]_*t*_ + [*L*]_*t*_ + *K*_d_)^2^-4[*P*]_*t*_ [*L*]_*t*_]^1/2^}/2[*P*]_*t*_.^54^ Data analysis was done in Bruker TopSpin 4.0.9 and NMRFAM-Sparky 1.470.60.^63^

### Crystallization

See the SI Methods section for detailed protein expression, purification, and crystallization protocols.

### Activity Data

All preincubations and cleavage reactions were carried out with wt M^pro^ in 50 mM sodium phosphate buffer (pH 7.0), 1 mM TCEP (only used as control without ligand), and 4% DMSO (when the ligand is added or for the DMSO control) with 0.5–10-fold excess of ligand. The preincubation was with protein at 200 μM for 1 or 24 h at 22 °C. For the cleavage reaction, we diluted M^pro^ to 100 μM with the addition of the substrate FLAG_3_-His_6_-SAVLQ-nsp9 to a final concentration of 235–296 μM, allowing a multiple-turnover reaction with 2.35–2.96 excess of substrate over the enzyme. The cleavage reaction was detected for a time period of 1 min up to 24/48 h. For each activity experiment, we used apo M^pro^ with and without TCEP, or DMSO as references. Initial activity data and preincubation time dependence are tested at an enzyme/ligand ratio of 1:10, meaning 200 μM M^pro^ and 2 mM ligand during the preincubation, which then are diluted to 100 μM and 1 mM, respectively, for the cleavage reaction. For the concentration-dependent activity data, we used 24 h of preincubation and changed the ligand amount to reach ratios of protein/ligand 2:1, 1:1, 1:2, 1:4, 1:6, 1:8, and 1:10 for all compounds but aged Z604; for aged Z604, we used 1 h of preincubation and changed the ligand amount to reach ratios of 2:1, 1:1, and 1:2. For the DTT-dependent activity data, we used 1 h of preincubation and the corresponding ligand amount to reach ratios of 1:2 and 2 mM DTT. These reactions were analyzed by 16/5% (w/v) tricine SDS-PAGE as described elsewhere.^64^

## Supporting Information Available

Methods (NMR screening, crystallization); CSP analysis; docking, modeling, activity data; synthesis and analytics (mass spec. and NMR spec., purity of the compounds was determined by NMR spectroscopy); and a table of molecular formula strings for each final compound (PDF)
