# Supplementary material for: Targeting the Main Protease (Mpro, nsp5) by Growth of Fragment Scaffolds Exploiting Structure-Based Methodologies
Source: ACS Chem Biol. 2024 Jan 17;19(2):563–74. doi: 10.1021/acschembio.3c00720 (PMC10877576; doi:10.1021/acschembio.3c00720)
Supplement: Supplementary file 1 — cb3c00720_si_001.pdf [file cb3c00720_si_001.pdf]

Supporting Information

Targeting main protease (M^{Pro}, nsp5) by growth of fragment scaffolds exploiting structure-based methodologies.

Nadide Altincekic[+,*,1,2], Nathalie Jores[+,1,2], Frank Löhr[2,3], Christian Richter[1,2], Claus Ehrhardt[4], Marcel J. J. Blommers[5], Hannes Berg[1,2], Sare Öztürk[1], Santosh L. Gande[1,2], Verena Linhard[1,2], Julien Orts[6], Marie Jose Abi Saad[6], Matthias Bütikofer[7], Janina Karderli[7], B. Göran Karlsson[8,9] Ulrika Brath[8], Mattias Hedenström[10], Gerhard Gröbner[10], Uwe H. Sauer[11], Anastassis Perrakis[12], Julian Langer[13], Lucia Banci[14,15], Francesca Cantini[14,15], Marco Fragai[14,15], Deborah Grifagni[14], Tatjana Barthel[16], Jan Wollenhaupt[16], Manfred S. Weiss[16], Angus Robertson[17], Adriaan Bax[17], Sridhar Sreeramulu[1,2], and Harald Schwalbe[*,1,2].

[1] Institute for Organic Chemistry and Chemical Biology, Goethe University Frankfurt am Main, D-60438 Frankfurt, Germany
[2] Center of Biomolecular Magnetic Resonance (BMRZ), Goethe University Frankfurt am Main, D-60438 Frankfurt, Germany
[3] Institute of Biophysical Chemistry, Goethe University Frankfurt am Main, D-60438 Frankfurt, Germany
[4] Department of Biochemistry, University of Zurich, 8093 Zurich, Switzerland
[5] SavernaTherapeutics, 4105 Biel-Benken, Switzerland
[6] Department of Pharmaceutical Sciences, University of Vienna, Josef-Holaubek-Platz 2, 1090 Vienna, Austria
[7] ETH Zurich, Swiss Federal Institute of Technology, Laboratory of Physical Chemistry, 8093 Zürich, Switzerland
[8] Swedish NMR Centre, Department of Chemistry and Molecular Biology, University of Gothenburg, SE40530, Göteborg, Sweden
[9] SciLifeLab, University of Gothenburg, SE40530, Göteborg, Sweden
[10] Swedish NMR Centre, Department of Chemistry, University of Umeå, SE-90187 Umeå, Sweden
[11] Protein Production Sweden, Department of Chemistry, University of Umeå, SE-90187 Umeå, Sweden
[12] Oncode Institute and Division of Biochemistry, the Netherlands Cancer Institute, 1066CX Amsterdam, Netherlands
[13] Max Planck Institute of Biophysics, D-60438 Frankfurt am Main, Germany
[14] Magnetic Resonance Center and Department of Chemistry, University of Florence, Via L. Sacconi 6, 50019, Sesto Fiorentino, Italy. B) Consorzio Interuniversitario Risonanze Magnetiche Metalloproteine, Via L. Sacconi 6, 50019, Sesto Fiorentino, Italy.
[15] Consorzio Interuniversitario Risonanze Magnetiche Metalloproteine, Via L. Sacconi 6, 50019, Sesto Fiorentino, Italy.
[16] Macromolecular Crystallography, Helmholtz-Zentrum Berlin, Albert-Einstein-Str. 15, D-12489 Berlin, Germany
[17] LCP NIDDK, NIH, United States of America

* Email: altincekic@nmr.uni-frankfurt.de

***** Email: schwalbe@nmr.uni-frankfurt.de

+ These authors contributed equally to this work.

Table of content

SImethods.......3
Nsp5 constructs.......3
Screening data.......3
Crystallization.......3
Protein expression and purification.......3
Crystallization conditions.......4
Docking of primary screening hits.......5
NMR spectra.......6
Primary NMR screening data on Z604.......6
Properties of screening hits.......7
Z604 sensitivity towards reducing agents.......8
Z604 aging.......9
Assignment of wt nsp5.......10
Overview of effects of the various ligands on nsp5 spectra.......11
Time dependence of Z604 binding.......12
Chemical shift perturbations (CSPs).......13
Plot of CSPs against the corresponding amino acid.......13
Exemplary chemical shift perturbations of amino acids near the active site.......14
Modeling of Z604 and derivatives.......14
Activity data.......15
Summary of interaction data.......17
Crystal structure details.......18
Second binding site of 35b.......19
Synthesis of Z604 and its derivatives.......20
Synthesis of secondary and tertiary amines based on the scaffold of Z604.......20
Synthesis of peptide-like tertiary structured ligands based on the scaffold of Z604.......21
Synthesis of X77-like compounds containing Uracil moiety.......22
Analytic of all synthesized compounds.......24
Analytic of secondary and tertiary amines 30a-d and 31a-d based on the scaffold of Z604.......24
Analytic of peptide-like tertiary structured ligands based on the scaffold of Z604.......32
References.......39

SImethods

Nsp5 constructs

Wt:
SGFRKMAFPSGKVEGCMVQVTCGTTTLNGLWLDDVVYCPRHVICTSEDMLNPNYEDLLIRK
SNHNFLVQAGNVQLRVIGHSMQNCVLKLKVDTANPKTPKYKFVRIQPGQTFSVLACYNGSP
SGVYQCAMRPNFTIKGSFLNGSCGSVGFNIDYDCVSFCYMHHMELPTGVHAGTDLEGNFY
GPFVDRQTAQAAGTDTTITVNVLAWLYAAVINGDRWFLNRFTTTLNDFNLVAMKYNYEPLT
QDHVDILGPLSAQTGIAVLDMCASLKELLQNGMNGRTILGSALLEDEFTPFDVVRQCSGVTF
Q

$_{GHM}$nsp5: N-terminally additional amino acids Gly-His-Met

$_{GS}$nsp5: N-terminally additional amino acids Gly-Ser

$_{GS}$nsp5$_{GPH6}$: N-terminally additional amino acids Gly-Ser, C-terminally Gly-Pro-His-His-His-His-His-His

Screening data

Screening data with the 768 compounds involving DSI-poised library purchased from Enamine were conducted as previously described.[1] NMR screening against the DSI-poised library purchased from Enamine with 896 compounds employed 10 mM phosphate buffer (pH 7.6) containing 50 mM NaCl in D_2O, 0.04% NaN_3 and 3.85% d_6-DMSO at 298 K. The protein concentration was 11.5 µM and mixtures of max. 6 compounds with 320 µM each were added. STD experiments were recorded, on a 600 MHz Bruker spectrometer with cryoprobe, with irradiation for 3 s at 200 Hz power using trains of 50 ms gaussian pulses, number of scans 256, acquisition time 0.98 s, relaxation delay 2 s. The protein signal was filtered using a spinlock for 20 ms at a 10 kHz power level. S/N > 10 was taken as a hit, everything below was considered a negative. The screening against the Bionet library containing 428 compounds and the Maybridge library containing 800 compounds were performed on a Bruker 600 MHz Avance III HD spectrometer equipped with a 5 mm BBO cryogenic probe. In addition, the Bionet library was also screened using a Bruker 700 MHz equipped with a cryogenic QCI-F probe. Screening conditions were very similar for both libraries, using a 10 mM phosphate buffer (pH 7.4) containing 130 mM NaCl, 2.4 mM KCl, 10% D_2O and 8% (Bionet) or 1% (Maybridge) d_6-DMSO. The protein concentration was 10 µM and 9-11 ligands were added to a final concentration of 100 µM for each ligand. Samples (180 µL final volume) were transferred to 3 mm tubes. STD experiments were recorded using a pulse-train of 40 gauss pulses (50 ms each) for the on- and off-resonance irradiation with a field strength of 200 Hz. The length of the CPMG spin-lock in the T_2-experiments was 200 ms and the spin-lock in the pseudo 2D T_{1rho}-experiments were 10 ms and 200 ms with a field strength of 5 kHz. For the Bionet, ^{19}F T_2-experiments were recorded library was also screened using ^{19}F NMR spin-echo experiments with a total spin-echo time of 100 ms and with a 70 kHz Chirp inversion pulse.[2]

Crystallization

Protein expression and purification

Nsp5 for crystallization was prepared as follows: The nsp5 gene was cloned into a pGEX-6-1 vsector with a C-terminal His_6-tag by the Hilgenfeld group.[3] The provided plasmid was transformed into *E. coli* strain BL21-Star (DE3). An overnight culture was inoculated from transformed clones and transferred the next day into auto-induction medium.[4] The cells were grown at 37°C until OD = 0.6 and then temperature was lowered to 18°C for overnight expression. Cells were harvested the next day through centrifugation at 10.000xg for 10 min. Cell pellets were flash-frozen in liquid nitrogen until purification. For purification the cells were thawed and resuspended in 20 mM Tris (pH 7.8), 150 mM NaCl, 5 mM imidazole, 0.05% Tween. The cells were lysed via sonication and centrifuged at 40.000x g for 45 min at 4°C.

The supernatant was loaded onto a Ni-NTA affinity chromatography column, and the protein was eluted with a stepwise imidazole gradient up to 500 mM imidazole. The protein was dialyzed overnight with addition of 1:10 w/w PreScission into 20 mM Tris (pH 7.8), 150 mM NaCl, 1 mM DTT. The next day the cleaved protein was subjected to a Ni-NTA affinity chromatography column to remove the cleaved His_6-tag. The final purification step was size exclusion chromatography using a Superdex 75 column equilibrated in 20 mM Tris (pH 7.8), 150 mM NaCl, 1 mM TCEP, 1 mM EDTA. The fractions with the purified protein were pooled, concentrated to 5 mg/ml and flash-frozen in liquid nitrogen before storage at -80°C.

Crystallization conditions

Nsp5 was crystallized in 23.5% PEG 1.500, 0.2 M MIB (pH 7.4), 5% DMSO, 0.025 mM EDTA (pH 7.0) using the NT8 pipetting robot (Formulatrix) and MRC 3-lens 96-well low-profile plates. The final drop consisted of 200 nl protein, 100 nl reservoir and 50 nl 1:50 seed stock dilution and was equilibrated at 20°C against 40 µl reservoir. Initial seeds were kindly provided by Deniz Eris (PSI, Villigen, Switzerland) and predetermined the crystallization in the orthorhombic space group. Based on the crystals grown from the initial seeds, new seeds were prepared in the following way. Crystals from one drop were crushed and transferred into 50 µl reservoir solution. The mixture was vortexed four times for 30 s with 30 s on ice in between with a Seed Bead™ (Hampton Research). The final seed stock was diluted to 1:50, aliquoted and flash-frozen in liquid nitrogen. Crystals grew within 2 days. The soaking solution was the same as the crystallization solution except for an additional 5 mM of the respective compound. A soaking plate was prepared with 40 µl crystallization solution as reservoir and a 0.4 µl drop of the respective soaking solution. The crystals were transferred from the crystallization plate into the soaking drops. After the transfer the plate was sealed with crystallization foil and incubated overnight at 20°C. The next day the crystals from each soaking drop were harvested, flash-frozen in liquid nitrogen and stored until measurement.

Data collection was performed at BL14.1 (BESSY II, Berlin, Germany).[5] The data was collected with 2000 images in 0.1° increments, an exposure time of 0.08 s, a 100 µm aperture and at 13.5 keV. The collected data was then processed automatically via XDSAPP[6] and afterwards automatically refined with fspipeline.[7] The input model for fspipeline was an inhouse refined MPro model, based on a molecular replacement with the PDB model 7BB2. For the dataset of 37b the binding could be confirmed in the electron density and the ligand could be fitted into the electron density manually after the fspipeline run. The resulting model was then further subjected to iterative cycles of manual building in coot[8] and refinement using phenix.refine.[9] The nsp5-37b co-crystal structure is deposited at the PDB with the ID 8PH4. The table with data collection and refinements statistics are given in table S3.

Docking of primary screening hits

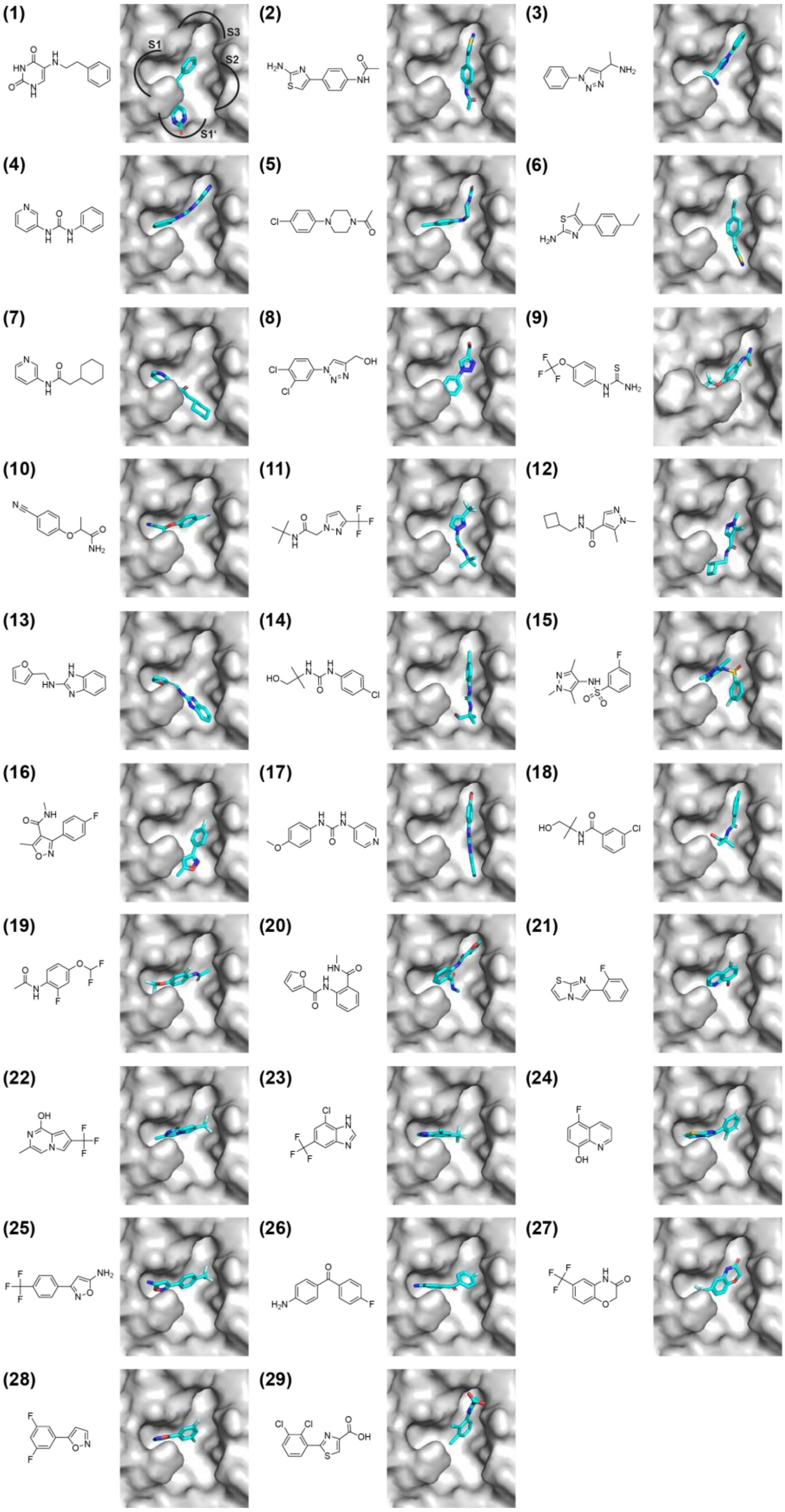

Figure S1: Docking poses of primary screening hits 1-29 generated by SwissDock[10, 11] using the nsp5 crystal structure PDB 5R83 (monomeric) showing the active site pocket (s. Table S1 for all fragments screened). The numbers in the structures represent the manuscript IDs of the 29 primary screening hits of four libraries.

NMR spectra

Primary NMR screening data on Z604

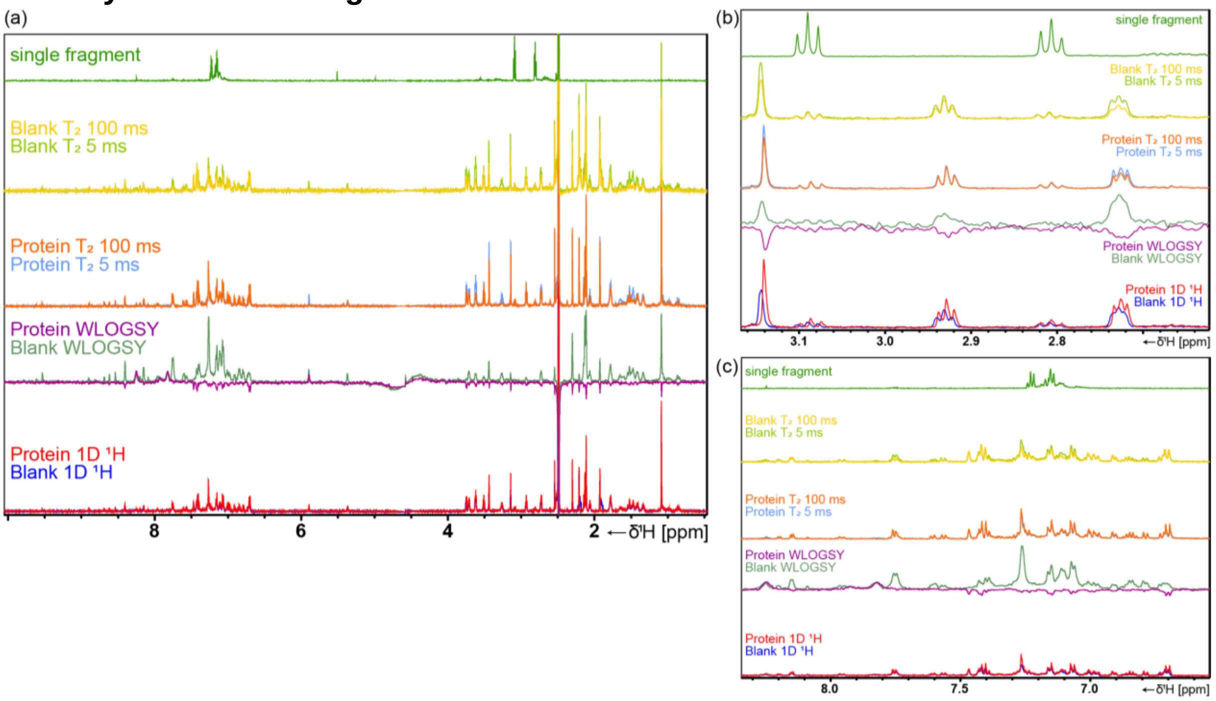

Figure S2: NMR spectra (1D ^{1}H, waterLOGSY, and T_2-CPMG (5 ms and 100 ms)) of commercially acquired Z604 in a mixture of 12 fragments in presence of GHMnsp5. A single fragment spectrum is used for chemical shift deconvolution in the mixture. Z604 shows sign changes in the waterLOGSY in presence of GHMnsp5. At the start of this project, we classified this compound as a potential hit after confirmation of its binding properties by other biophysical methods (SPR, TSA). The sample was prepared with 10 µM GHMnsp5, 200 µM of each ligand in 25 mM NaPi (pH 7.5), 150 mM NaCl, 5% d_6-DMSO.

Properties of screening hits

	No	M [g/mol]	cLogP	cLogS	Water solubility [mM]	H-Acceptors	H-Donors	Total Surface Area	Relative PSA	Polar Surface Area	Drug likeness	Molecular Flexibility	Molecular Complexity	Electro-negative Atoms	Amides	Amines	Alkyl amines	Aromatic Amines	Aromatic Nitrogens
a) DSI-poised	1	231.25	0.261	-2.610	2.454709	5	3	181.87	0.332	70.23	3.36	0.518	0.611	5	2	0	0	0	0
	2	233.29	2.062	-3.545	0.285102	4	2	177.71	0.400	96.25	3.66	0.413	0.630	5	1	1	0	1	1
	3	188.23	-0.180	-1.395	40.271703	4	1	153.02	0.288	56.73	-2.91	0.419	0.624	4	0	1	1	0	3
	4	213.24	1.800	-2.849	1.415794	4	2	172.45	0.272	54.02	-0.02	0.465	0.527	4	2	0	0	0	1
	5	238.72	2.101	-2.239	5.767665	3	0	180.83	0.111	23.55	6.02	0.473	0.605	4	1	1	0	1	0
	6	218.32	3.543	-4.160	0.069183	2	1	174.52	0.267	67.15	1.05	0.456	0.652	3	0	1	0	1	1
	7	218.30	2.161	-2.759	1.741807	3	1	182.79	0.194	41.99	-3.57	0.476	0.559	3	1	0	0	0	1
	8	244.08	1.143	-2.720	1.905461	4	1	170.69	0.246	50.94	-3.03	0.438	0.721	6	0	0	0	0	3
b) DSI-poised (extended)	9	236.22	2.237	-3.328	0.469894	3	2	165.41	0.391	79.37	-8.98	0.634	0.523	7	0	0	0	0	0
	10	190.20	0.517	-2.636	2.312065	4	1	156	0.333	76.11	-5.47	0.456	0.520	4	1	0	0	0	0
	11	249.24	0.704	-1.444	35.974934	4	1	180.84	0.234	46.92	-7.33	0.677	0.656	7	1	0	0	0	2
	12	207.28	0.625	-1.842	14.387986	4	1	168.99	0.251	46.92	5.89	0.405	0.644	4	1	0	0	0	2
	13	213.24	1.883	-3.163	0.687068	4	2	170.86	0.296	53.85	1.00	0.416	0.673	4	0	1	0	1	2
	14	242.71	1.918	-3.153	0.703072	4	3	184.39	0.266	61.36	1.01	0.534	0.556	5	2	0	0	0	0
	15	283.33	0.918	-2.766	1.713957	5	1	204.15	0.284	72.37	-1.38	0.479	0.777	7	1	0	0	0	2
	16	234.23	1.758	-3.178	0.663743	4	1	181.55	0.273	55.13	1.30	0.450	0.786	5	1	0	0	0	1
	17	243.27	1.730	-2.867	1.358313	5	2	194.71	0.292	63.25	0.05	0.485	0.541	5	2	0	0	0	1
	18	227.69	1.924	-2.637	2.306747	3	2	172.93	0.217	49.33	1.42	0.573	0.613	4	1	0	0	0	0
	19	219.16	1.634	-2.894	1.276439	3	1	156.28	0.221	38.33	-2.28	0.465	0.652	6	1	0	0	0	0
	20	244.25	1.435	-2.815	1.531087	5	2	193.39	0.326	71.34	2.40	0.390	0.726	5	2	0	0	0	0
c) Bionet	21	218.26	2.584	-2.459	3.475362	2	0	159.32	0.247	45.54	0.12	0.341	0.740	4	0	0	0	0	2
	22	203.16	2.212	-2.736	1.836538	2	1	144.19	0.170	29.1	-6.72	0.526	0.567	5	1	0	0	0	0
	23	216.16	1.571	-3.427	0.374111	3	1	145.34	0.220	37.53	-5.43	0.317	0.798	6	0	0	0	0	2
	24	220.58	2.714	-3.341	0.456037	2	1	141.07	0.177	28.68	-7.08	0.336	0.732	6	0	0	0	0	2
	25	163.15	1.731	-2.340	4.570882	2	1	119.32	0.202	33.12	-3.22	0.000	0.762	3	0	0	0	0	1
	26	228.17	2.343	-3.870	0.134896	3	1	159.45	0.253	52.05	-6.60	0.439	0.684	6	0	1	0	1	1
	27	215.23	2.213	-4.106	0.078343	2	1	165.64	0.171	43.09	-2.22	0.512	0.630	3	0	1	0	1	0
	28	217.15	1.370	-2.600	2.511886	3	1	142.19	0.243	38.33	-6.51	0.326	0.740	6	1	0	0	0	0
	29	181.14	2.022	-3.119	0.760326	2	0	134.17	0.187	26.03	-1.07	0.303	0.674	4	0	0	0	0	1
d) Maybridge	30	274.13	3.066	-3.452	0.353183	3	1	182.66	0.315	78.43	3.66	0.383	0.763	6	0	0	0	0	1
	31	209.27	0.654	-2.620	2.398833	3	1	152.04	0.367	77.62	-0.07	0.240	0.720	4	0	1	0	1	0

Figure S3: Properties of the primary screening hits of all four libraries.

Z604 sensitivity towards reducing agents

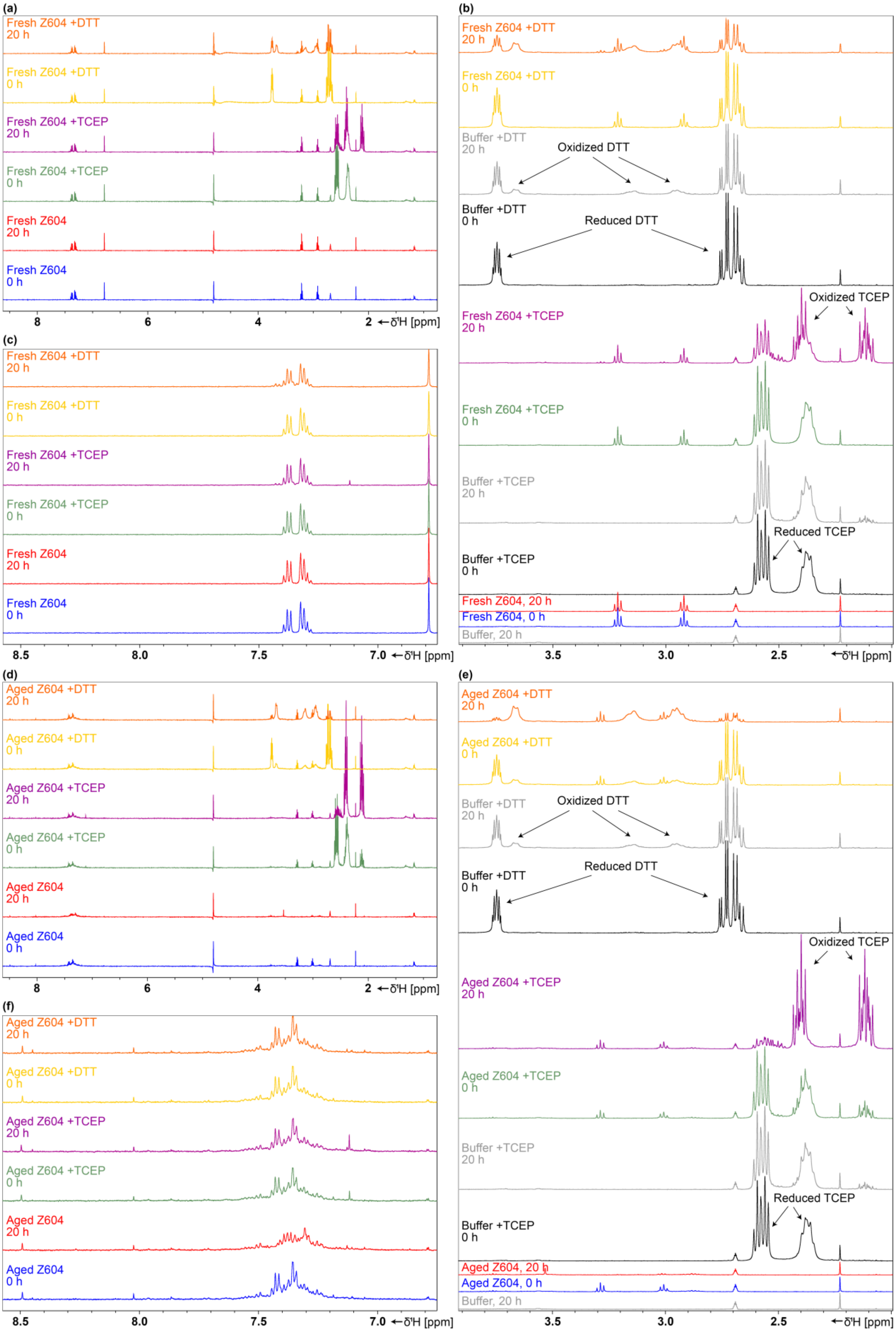

Figure S4: 1D-^{1}H spectra of 0.25 mM fresh (a-c) or aged (d-f) Z604 in 50 mM NaPi (pH 7.0), 10% D_2O, 0.25% d_6-DMSO in absence of reducing agent, in presence of 1 mM TCEP or 1 mM DTT. Both TCEP and DTT are faster oxidized in presence of Z604. In case of aged Z604 this process is accelerated. A new signal in the aromatic region (7.12 ppm) appears in presence of TCEP. The solubility/stability of aged Z604 is better in presence of reducing agent.

Z604 aging

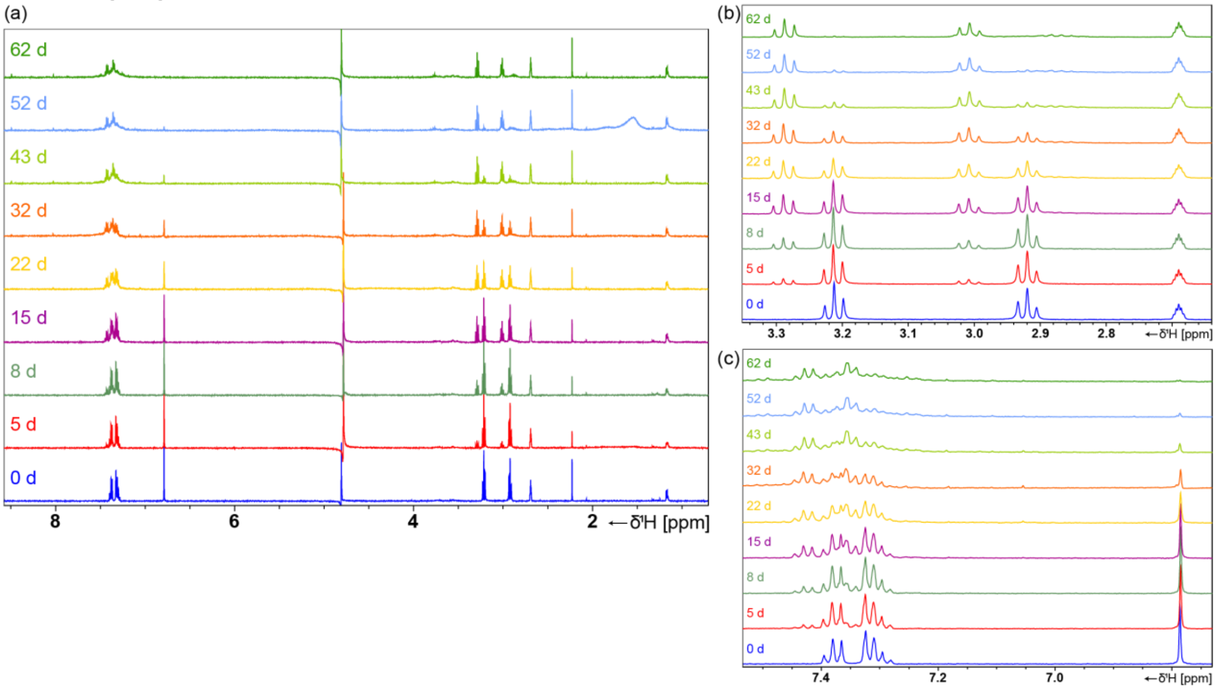

Figure S5: 1D-^{1}H spectra of Z604 (a), and sections of the aliphatic (b), and aromatic regions (c). A stock solution of 100 mM Z604 in DMSO is prepared and stored at rt to capture the aging. For each time point (0-62 d) a new NMR sample of 0.25 mM Z604 in 50 mM NaPi (pH 7.0), 10% D_2O, 0.25% d_6-DMSO is prepared and directly measured. During the aging at rt the Z604 stock changes its color from colorless to pink/red/brown. A second signal set appears with time and gets more intense. After 62 d the second signal set yields ~92%. The same NMR samples are measured after a few days and the second set of signals disappears hinting at instability or limited solubility in buffer. Data not shown but visible in SIfig. 4(e). The second signal set disappears after 20 h in absence of reducing agent (red and blue spectra).

Assignment of wt nsp5

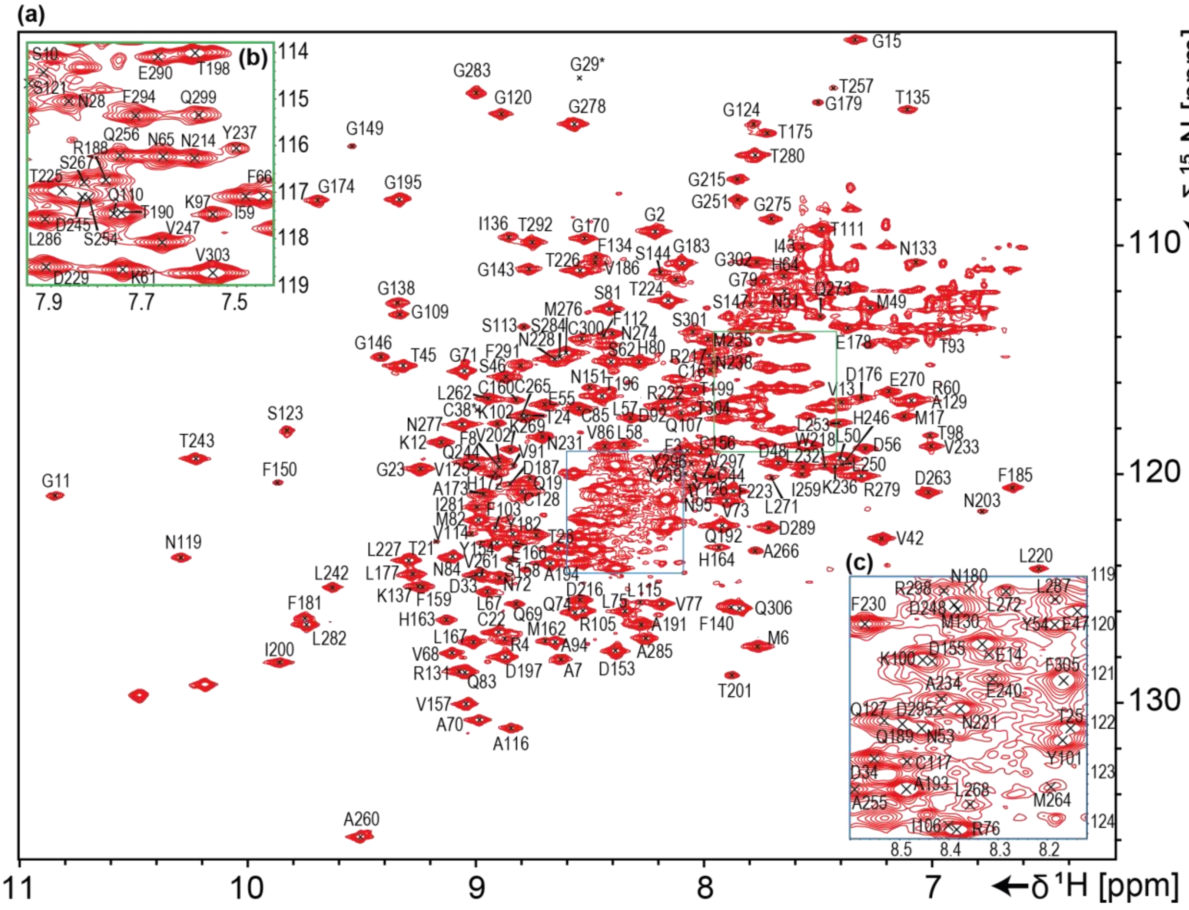

Figure S6: [^{15}N, ^{1}H]-BEST-TROSY of wt nsp5 with backbone assignment to a degree of 83% (86% when leaving out proline residues and the N-terminal Ser). (a) shows the whole spectrum, (b) and (c) show sections, which are marked by color in the full spectrum.

Overview of effects of the various ligands on nsp5 spectra

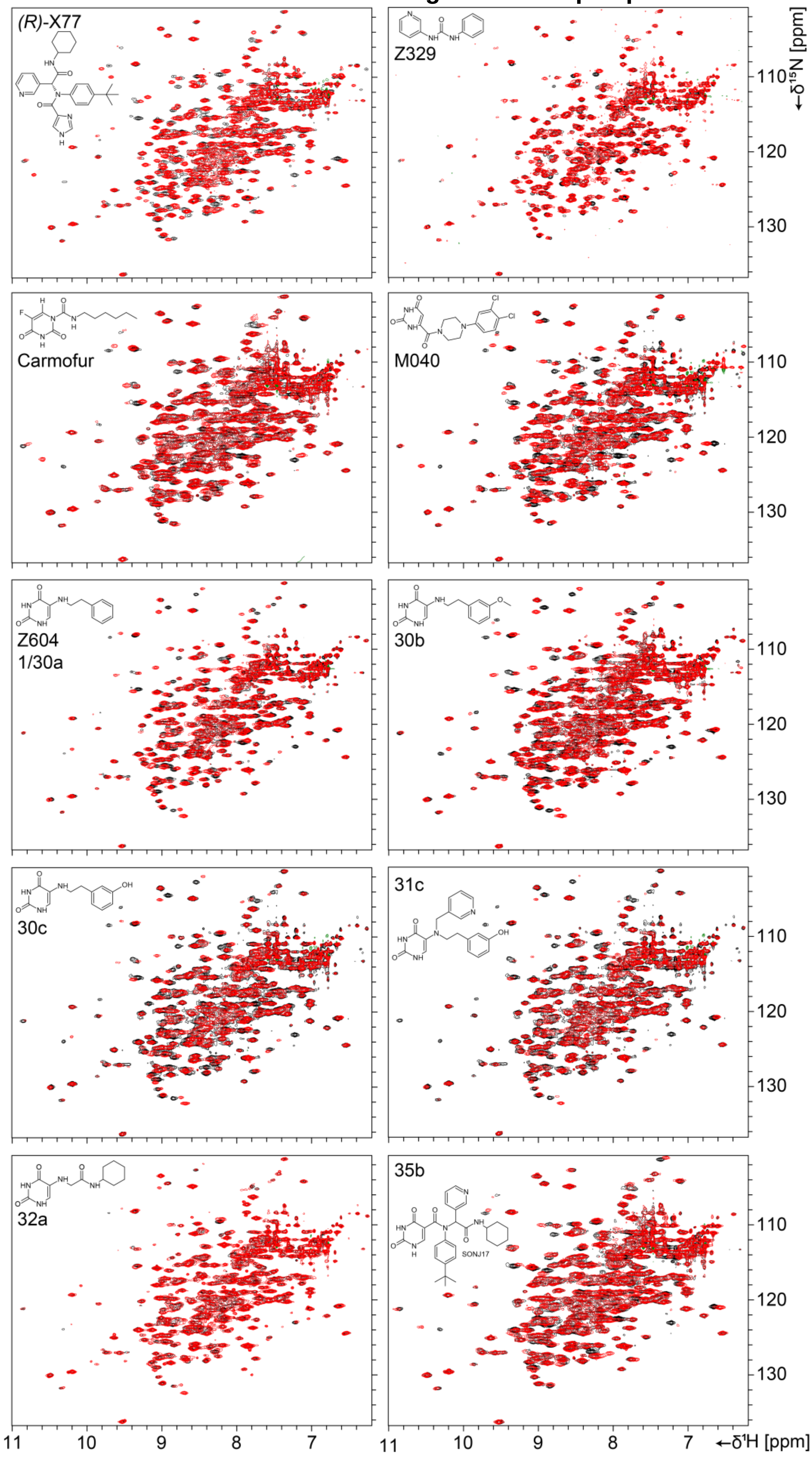

Figure S7: 2D [^{15}N, ^{1}H]-NMR-spectra of apo nsp5 (black), overlayed with nsp5 and the ligands *(R)*-X77, Z329, Carmofur, M040, and Z604 (**1/30a**), **30b**, **30c**, **31c**, **32a**, and **35b** (red). All spectra are of the [^{15}N, ^{1}H]-BEST-TROSY type. The protein concentration ranges from 100 to 200 µM with a 20-fold excess of ligand in case of Z329, 10-fold in case of M040, Z604, **30b**, **30c**, **31c**, **32a**, and **35b**, 2-fold for Carmofur and 1.5-fold for *(R)*-X77. The reference apo spectra are measured with the corresponding DMSO amount. *(R)*-X77, Z329, Carmofur, and M040 experiments are conducted directly after ligand addition. Z604, **30b**, **30c**, **31c**, **32a**, and **35b** experiments are measured after storage at rt for 10-44 h due to their time dependent binding (s. Figure S2). Detailed sections of two affected amino acids by ligand addition are shown in Figure S4.

Time dependence of Z604 binding

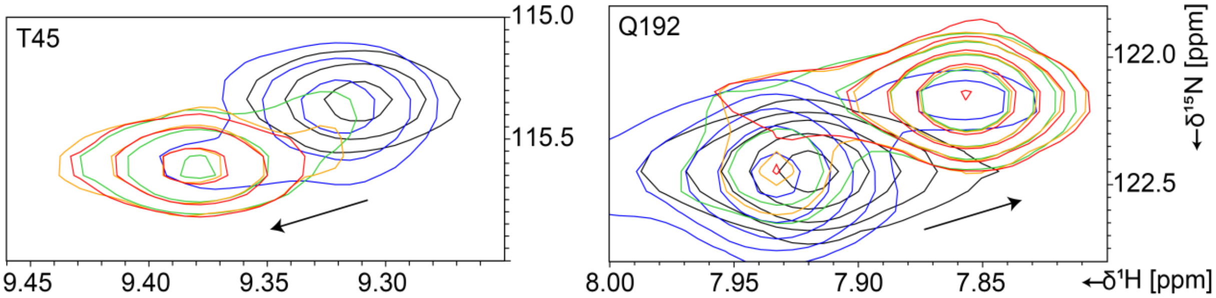

Figure S8: Time dependence of Z604 binding shown exemplary by sections of T45 and Q192. BEST-TROSY spectra of apo nsp5 (black), overlayed with Z604 spectra after 0 h (blue), 5.5 h (green), 11 h (orange), and 16.5 h (red) after ligand addition (same samples as in SIfig. 1). The arrow shows the direction of the CSP.

Chemical shift perturbations (CSPs)

Plot of CSPs against the corresponding amino acid

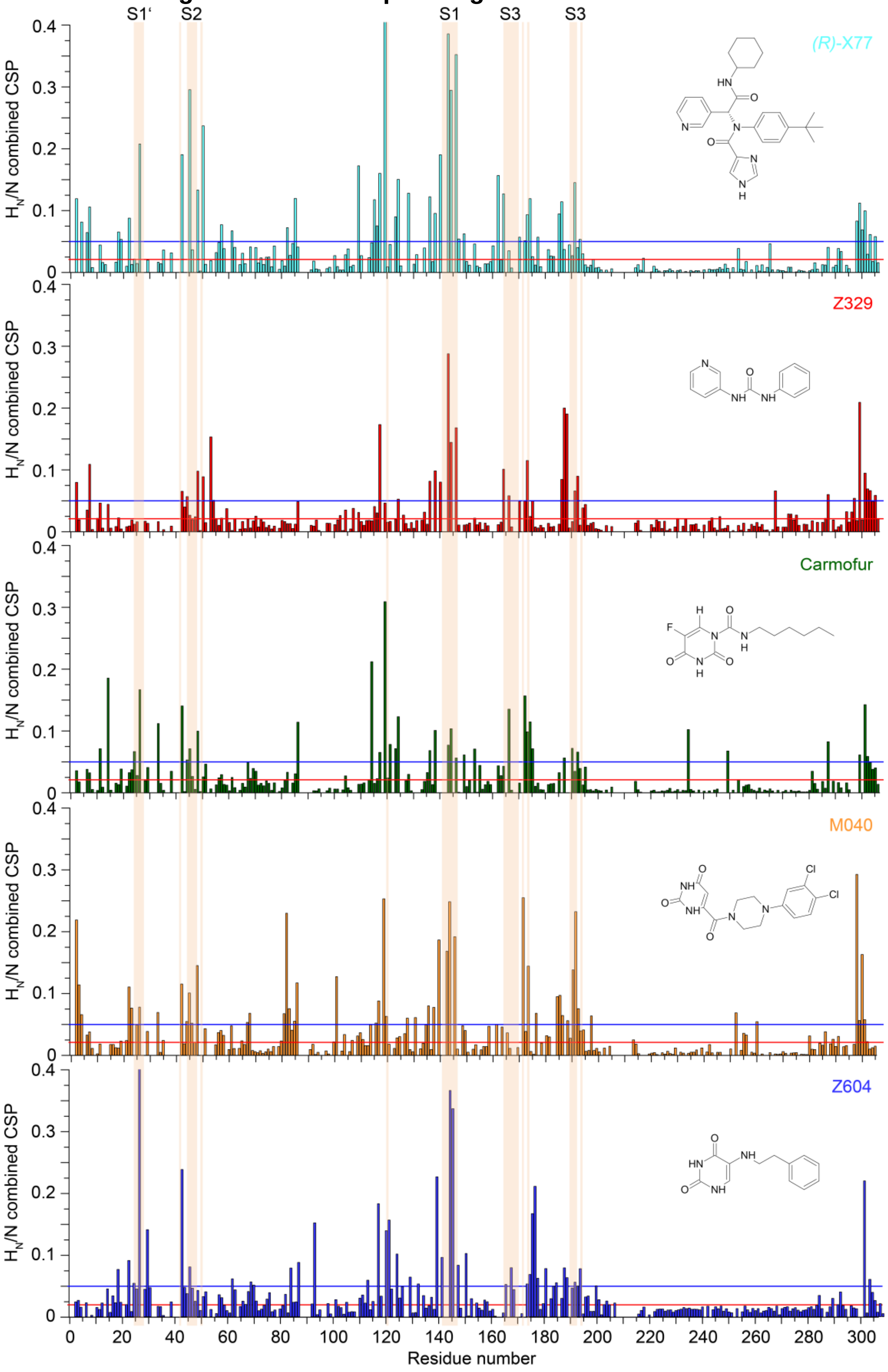

Figure S9: CSP plots of *(R)*-X77, Z329, Carmofur, M040, and Z604 determined from spectra of FigureS1 are plotted against the corresponding amino acid by use of $\Delta\delta = \sqrt{(\frac{\Delta\delta_N}{6.5})^2 + (\Delta\delta_H)^2}$.[12] Light pink shade shows the active site amino acids.

Missing values represent either prolines or unassigned residues (S1, K5, V20, L27, L30, W31, L32, V36, Y37, R40, H41, N63, I78, L87, K88, L89, K90, Y118, S139, L141, N142, C145, V148, I152, Y161, M165, T169, V171, V204, A206, W207, L208, Y209, A210, A211, V212, I213, F219, E288).

Exemplary chemical shift perturbations of amino acids near the active site

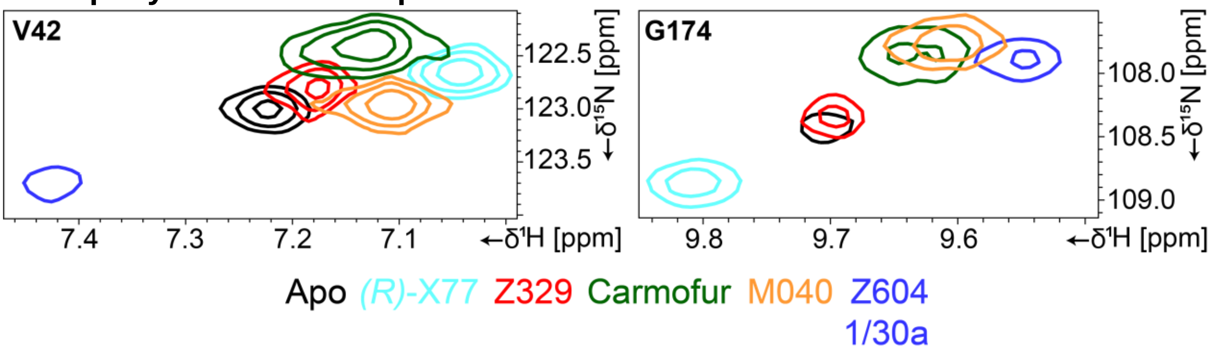

Figure S10: Sections of overlayed [^{15}N, ^{1}H]-BEST-TROSY spectra of apo nsp5 (black) and nsp5 with ligands (color coding on the bottom of figure) showing amino acids V42 and G174 that are affected by the ligand presence (full spectra in Figure S1).

Modeling of Z604 and derivatives

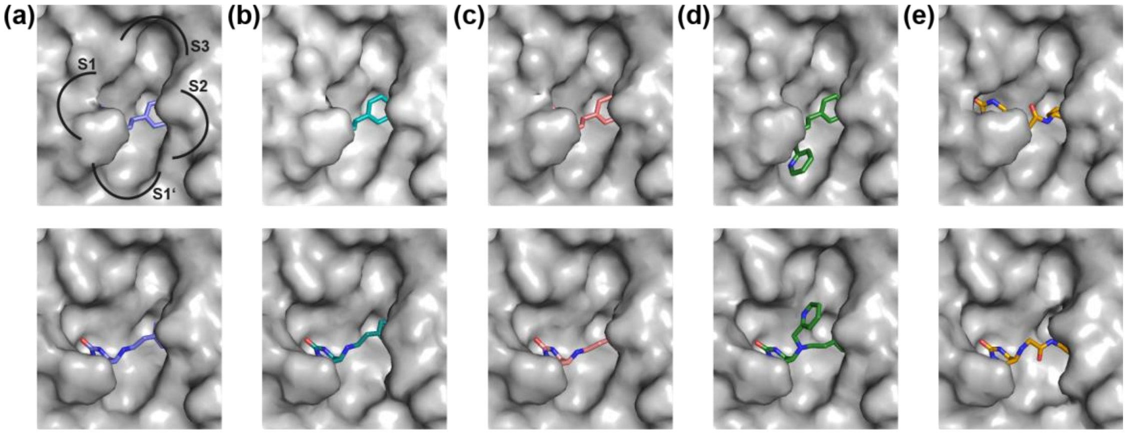

Figure S11: Modeling of each ligand within the active site pocket of nsp5 including amino acids N142 and Q189 (upper row: non-covalent, lower row: covalent binding of ligand)

To generate the docking poses the PDB structure 6LU7 was used with a covalent ligand placed the uracil close to the cyclic Gln of the covalent ligand (S-1) and the phenyl ring close to the Leu of the ligand (S-2). After several attempts it was found that a trans-arrangement looked best for the sulfur attack of Cys 145, since then hydrogen bonds could be modeled for all polar groups of the uracil, namely to the backbone NH of Gly 143, the backbone C=O of Leu 141 and His 146 and to the side chain N(ε)H of His 163. This model was created manually. All structures have been finally minimised within the CHARMM22 forcefield.

Activity data

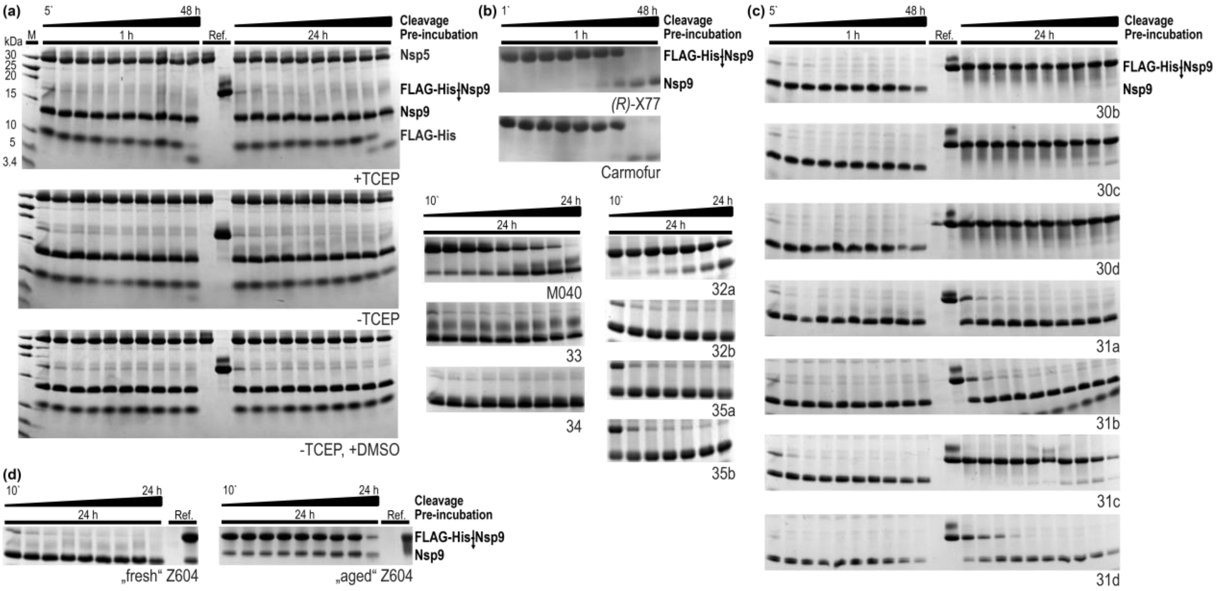

Figure S12: Activity assays of nsp5[13] in presence of ligands Z604, **30b**, **30c**, **30d**, **31a**, **31b**, **31c**, **31d**, **33**, **34**, **32a**, **32b**, **35a**, **35b** and the reference compounds *(R)-X*77, *Z*329, Carmofur, and M040. The controls include apo nsp5 without reducing agent (as for all samples with ligands), with TCEP, and with DMSO (as for all samples with ligands). All samples were pre-incubated to allow binding prior to substrate addition that starts the cleavage reaction.

Pre-incubation with 200 µM nsp5 in 50 mM NaPi (pH 7.0), without/with 0.1-2 mM ligand, without/with 4% DMSO (present for all ligands and the DMSO reference), without/with 1 mM TCEP (present only for the TCEP reference) was at 22°C for 1 h or 24 h. The pre-incubation time is indicated in the figure if both times are tested.

Cleavage reactions are started by the addition of substrate FLAG$_3$-His$_6$-SAVLQ-nsp9 (235-296 µM) diluting nsp5 to 100 µM, ligand to 0.05-1 mM, DMSO to 2%, while the buffer stayed the same. The cleavage time is indicated in the figure by a bar with the starting and end point.

All cleavage reactions were analyzed by SDS-PAGE and for all a protein marker (M, PageRuler™ Unstained Low Range Protein Ladder (Thermo Scientific™)), nsp5 (Ref.), and FLAG$_3$-His$_6$-SAVLQ-nsp9 (Ref.) were used. The gels in (a) are exemplary shown in full showing the marker and nsp5. All other gels are cut to only show full-length substrate and cleaved nsp9. All protein/peptide migration levels are indicated on the right of the gels. Gels are labeled as +TCEP for nsp5 with TCEP; -TCEP, +DMSO for nsp5 with DMSO; and -TCEP for nsp5 without reducing agent; or with the corresponding ligand.

(a) Control experiments with apo nsp5. The substrate concentration is 235 µM, and the cleavage reaction is stopped after 5‘, 15‘, 30‘, 1 h, 2 h, 4 h, 6 h, 8 h, 24 h, 48 h. Both pre-incubation times of 1 h and 24 h are tested.

(b) Activity assay for references *(R)-X*77, Carmofur, M040 and the third generation ligands. For *(R)*-X77, and Carmofur the substrate concentration is 291 µM, and the cleavage reaction is stopped after 1‘, 5‘, 10‘, 20‘, 45‘, 2 h, 4.5 h, 24 h, 48 h. For M040, **33**, **34** the substrate concentration is 284 µM, and the cleavage reaction is stopped after 10‘, 20‘, 40‘, 1 h, 2 h, 4 h, 6 h, 8 h, 24 h. For **32a**, **32b**, **35a**, **35b** the substrate concentration is 275 µM, and the cleavage reaction is stopped after 10‘, 30‘, 1 h, 2 h, 4 h, 6 h, 24 h.

(c) Activity assay for all first and second generation ligands. Substrate concentration is 235 µM, and the cleavage reaction is stopped after 5‘, 15‘, 30‘, 1 h, 2 h, 4 h, 6 h, 8 h, 24 h, 48 h. Both pre-incubation times of 1 h and 24 h are tested.

(d) Activity assay for freshly prepared Z604 (only first signal set in NMR visible) and aged Z604 (ratio of first to second signal set is 2:1). Substrate concentration is 284 µM, and the cleavage reaction is stopped after 10‘, 20‘, 40‘, 1 h, 2 h, 4 h, 6 h, 8 h, 24 h.

Summary of interaction data

Table S2: Summary of most promising compounds Z604, **30b**, **30c**, **31c**, and the references *(R)*-X77, Z329, Carmofur, M040.

Cov. = covalent, rev./irrev. = reversible/irreversible; * = 1-best, 6-worst; cdnt = conc. dependence not tested.

	(R)-X77	Z329	Carmofur	M040	Z604	30b	30c	31c	32a	35b
PDB	6W63	5R83	7BUY	7N8C						8PH4
Binding mode	Non-cov.	Non-cov.	Irrev. cov.	Non-cov.	Cov.					Non-cov.
K_D / µM	0.057[14]	461[1]		1.3 ± 0.18[15]						
CSP for										
V42	0.190	0.065	0.140	0.115	0.238	0.231	0.247	0.232	0.021	0.103
V86	0.041	0.049	0.114	0.117	0.088	0.084	0.094	0.082	0.063	0.041
G143	0.386	0.288	0.077	0.169	0.366	0.343	0.339	0.369	0.384	0.022
S144	0.295	0.145	0.104	0.248	0.336	0.319	0.311	0.326	0.326	0.212
E166	0.035	0.058	0.135	0.036	0.079	0.086	0.085	0.083	0.030	0.046
H172	0.051	0.050	0.157	0.255	0.053	0.056	0.056	0.053	0.052	0.066
G174	0.119	0.025	0.115	0.144	0.167	0.184	0.184	0.201	0.193	0.072
Q192	0.040	0.090	0.066	0.232	0.077	0.089	0.089	0.081	0.074	0.129
Relative inhibitory effect*	cdnt	6	3	cdnt	5	2	1	4	cdnt	cdnt

Crystal structure details

Table S3: Crystal structure data collection and refinement statistics.

Protein	Nsp5
Ligand	**35b**
(PDB ligand ID)	(YQN)
**Data collection**	
Space group	$P2_12_12_1$
a, b, c (Å)	67.69 100.04 103.37
α, β, γ (°)	90.0 90.0 90.0
Resolution limit (Å)	49.28-1.69 (1.79-1.69)
Unique reflections	78838 (12568)
Multiplicity	7.21 (7.1)
Mean I/σ(I)	10.76 (0.93)
ISa	20.23
R_{meas} (%)	12.6 (203.1)
Completeness (%)	99.5 (99.4)
CC (1/2)	99.8 (38.1)
**Refinement**	
R_{work} / R_{free} (%)	22,3 / 24,9
No. of atoms	5138
Protein	4728
Ligands	161
Water	249
**Ramachandran Plot**	
Favored / Allowed (%)	98 / 2
RMSZ bonds	0.37
RMSZ angles	0.60
Average *B*-factor	28,0
**PDB ID**	**8PH4**

Second binding site of 35b

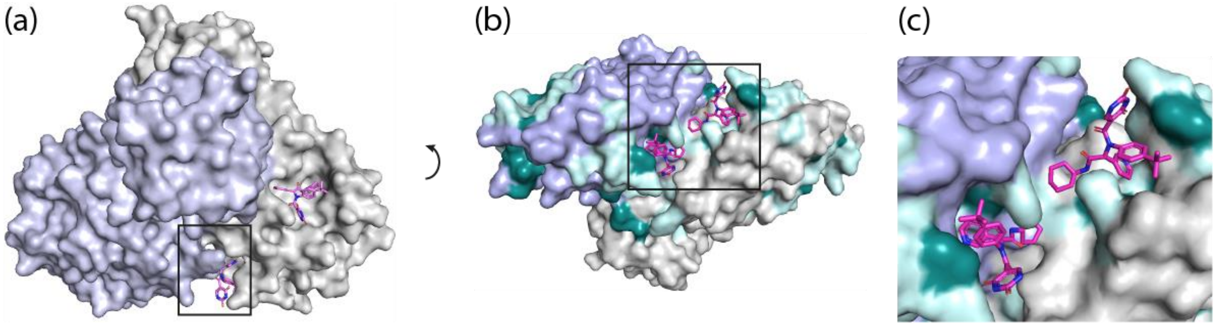

Figure S13: The co-crystal structure of nsp5 with **35b** as a dimer (a). Tilted view to show the second binding site (b) and a section (c). The light tale colored amino acids show CSPs > 0.02, and tale colored amino acids show CSPs > 0.05 at a 10-fold excess of ligand over protein (s. Figure S7) supporting the allosteric binding site.

Synthesis of Z604 and its derivatives

Synthesis of secondary and tertiary amines based on the scaffold of Z604

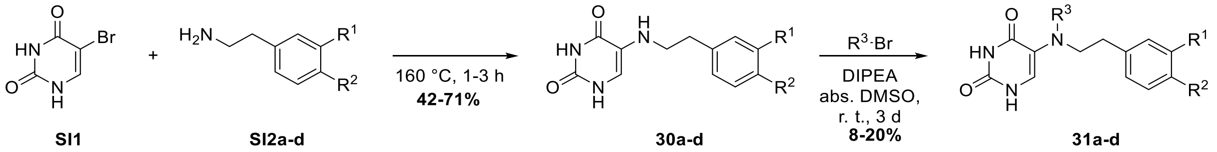

[1] [2]

Scheme S1: Reaction to synthesize secondary [1] and their corresponding tertiary amines [2].

Compound	R^1	R^2	R^3	Reaction time	Yield [%]
**Z604/30a**	H	H	H	1 h	51
**30b**	O-CH_3	H	H	1 h	71
**30c**	OH	H	H	3 h	43
**30d**	H	OH	H	2 h	42
**31a**	H	H	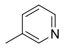	3 d	15
**31b**	O-CH_3	H	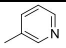	3 d	8
**31c**	OH	H	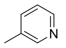	3 d	11
**31d**	H	OH	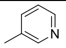	1 d	20

[1] The general reaction procedure was based on a synthesis known from literature.[16, 17]

Bromuracil **SI1** (1 eq) and amine **SI2a-d** (1 eq) reacted without solvent at 160 °C in the melt for 1-3 h. Dest. water was added to the reaction mixture and the pH was adjusted to neutral with 10% HCl. In **30a** and **30b** solids precipitated, these were filtered off and characterized as clean product. For product **30c**, the whole reaction mixture was adsorbed on Celite and purified by reversed phase flash chromatography (H_2O→MeCN). Products **30a-d** were clearly identified by NMR spectroscopy and mass spectrometry. The spectra and chromatograms can be found in the appendix.

[2] The general reaction procedure was based on a synthesis known from literature.[18]

The secondary amine **30a-d** (1.0 eq) was dissolved in 5 mL dry DMSO and Bromomethylpyridine (1.1 eq) and DIPEA (5.0 eq) were added under *Schlenk* conditions. The reaction mixture was stirred for 1-3 days at room temperature. The solvent was removed under reduced pressure and the purification was done via reversed phase flash chromatography (H_2O→MeCN) following HPLC. Products **31a-d** were clearly identified by NMR spectroscopy and mass spectrometry. The spectra and chromatograms can be found in the appendix.

Synthesis of peptide-like tertiary structured ligands based on the scaffold of Z604

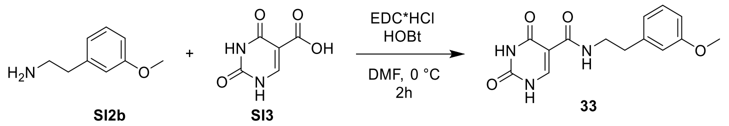

Scheme S2: Reaction to synthesize compound **33**.

To obtain product **33**, **SI2b** (1.0 eq), **SI3** (1.0 eq) and HOBt (0.1 eq) were dissolved in 4 mL DMF and cooled to 0 °C. EDC (1.5 eq) was added and the mixture was stirred for 10 min at 0 °C, heated up to room temperature and stirred for 8 h at room temperature. The solvent was removed under reduced pressure and the purification was done via reversed phase flash chromatography (H_2O→MeCN). Product **35** was clearly identified by NMR spectroscopy and mass spectrometry. The spectra and chromatograms can be found in the SI/appendix.

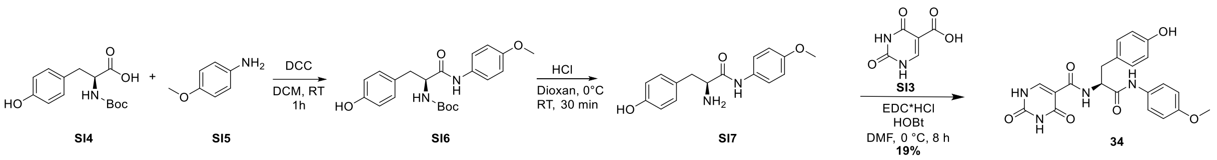

Scheme S3: Reaction to synthesize compound **34**.

Boc-*L*-tyrosine **SI4** (1.0 eq), *p*-Anisidine **SI5** (1.0 eq) and DCC (1.2 eq) were dissolved in 5 mL DCM. The reaction mixture was stirred for 1 h at room temperature. A colorless precipitate was filtered and identified as the urea by-product. The filtrate was washed with sat. $NaHCO_3$ solution and extracted with DCM (3x). The solvent was removed under reduced pressure. For deprotection of the raw product **SI6**, the boc group was directly eliminated by dissolving in 1,4-dioxane/HCl (4 M) at 0 °C, the mixture was then stirred for 1 h at room temperature. The solvent was removed under reduced pressure and the purification was done via normal phase flash chromatography (DCM:MeOH, 10:1). Product **SI7** was clearly identified by NMR spectroscopy and mass spectrometry. The spectra and chromatograms can be found in the appendix.

To obtain product **34**, **SI7** (1.0 eq),**SI3** (1.0 eq) and HOBt (0.5 eq) were dissolved in 4 mL DMF and cooled to 0 °C. EDC (2.0 eq) was added and the mixture was stirred for 10 min at 0 °C, heated up to room temperature and stirred for 8 h at room temperature. The solvent was removed under reduced pressure and the purification was done via normal phase flash chromatography (DCM:MeOH, 10:1) following HPLC. Product **34** was clearly identified by NMR spectroscopy and mass spectrometry. The spectra and chromatograms can be found in the appendix.

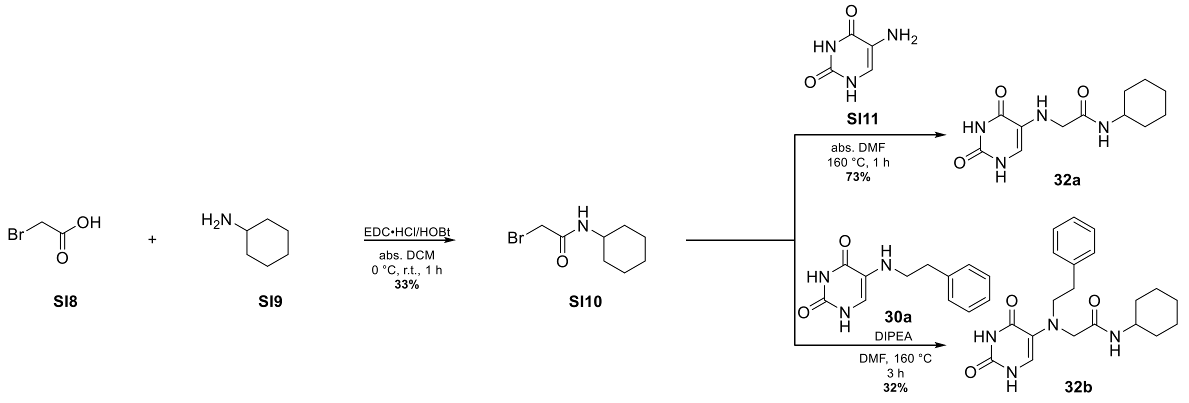

Sscheme S4: Reaction to synthesize compounds **32a-b**.

To obtain **SI10**, **SI8** (1 eq), EDC·HCl (1 eq) and HOBt (1 eq) were dissolved in 5 mL abs. DMF at 0 °C. **SI9** (1 eq) was added and the reaction mixture was stirred for 15 min at 0 °C, heated up to room temperature and stirred for 1 h. For purification, the reaction mixture was diluted with EtOAc, then washed three times each with 20 mL of 1 M HCl, $NaHCO_3$ solution and NaCl solution. The organic layers were combined and the solvent was removed under reduced pressure and purified by reverse-phase flash chromatography (Celite, H_2O→ACN). Product **SI10** was clearly identified by NMR spectroscopy and mass spectrometry.

To obtain products **32a-b,** compound **SI10** (1 eq) and **SI11/30a** (3 eq) were dissolved in 2-3 mL abs. DMF. The reaction mixture was stirred at 160 °C for 3 h, cooled to room temperature and stirred for 1 h. The solvent was removed under reduced pressure and the crude product purified by reversed-phase flash chromatography (Celite, H_2O→ACN). Products **32a-b** were clearly identified by NMR spectroscopy and mass spectrometry. The spectra and chromatograms can be found in the appendix.

Synthesis of X77-like compounds containing Uracil moiety

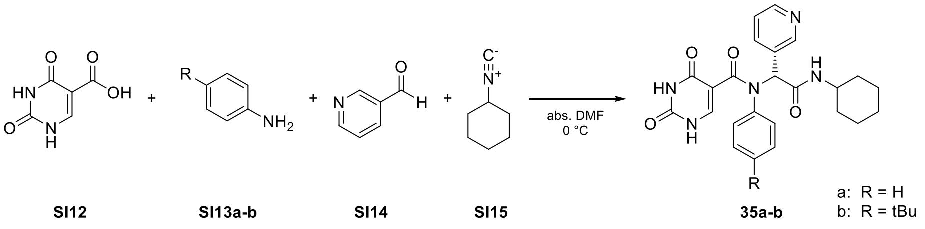

Scheme S5: Reaction to synthesize compounds **35a-b**.

Reactants **SI12** (1 eq), **SI13a-b** (1 eq) and **SI14** (1 eq) were dissolved in 6.5 mL abs. DMF. The mixture was cooled to 0 °C in an ice bath, reactant **SI15** (1 eq) was added and stirred for 1 h. Additionally the reaction mixture was stirred overnight at room temperature. For purification, the reaction mixture was extracted with DCM and washed with water and brine.

After combining the organic phases, the solvent was removed under reduced pressure. The oily crude product was purified by reversed-phase flash chromatography (Celite, H_2O→MeCN). The selected isolated fractions were combined and the solvent was removed under reduced pressure. A yellow solid was obtained, which was additionally purified by normal phase flash chromatography (silica gel, DCM → DCM/MeOH 4:1). Products **35a-b** were clearly identified by NMR spectroscopy and mass spectrometry. The spectra and chromatograms can be found in the appendix.

Analytic of all synthesized compounds

Analytic of secondary and tertiary amines 30a-d and 31a-d based on the scaffold of Z604

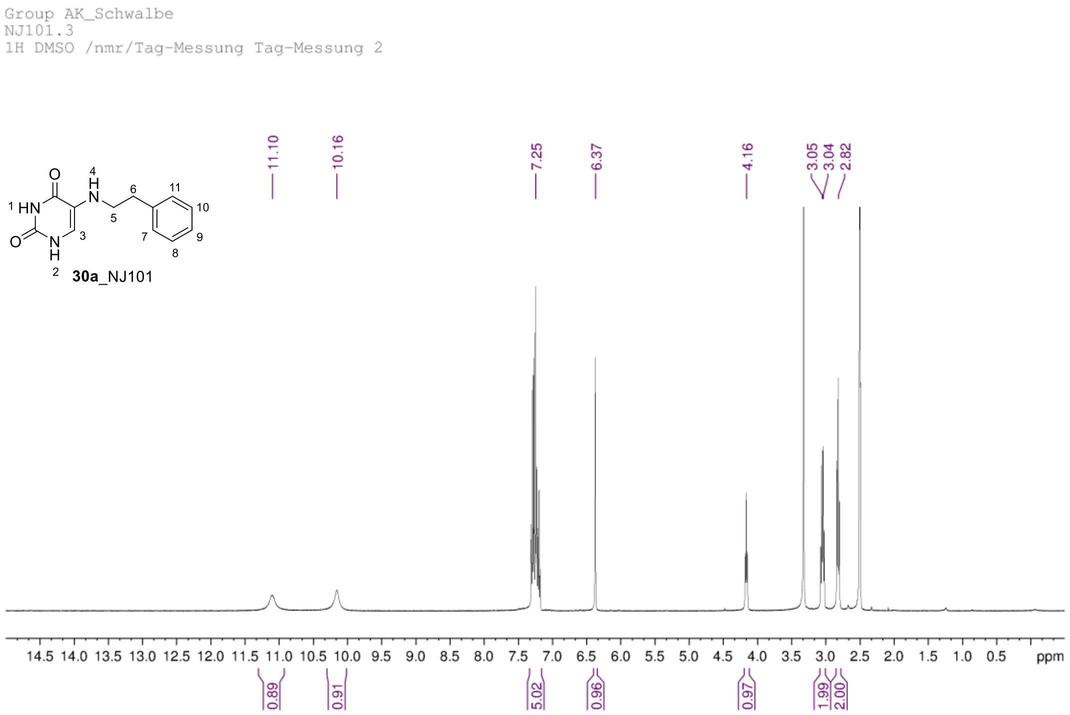

^{1}H-NMR (400.26 MHz, DMSO-*d*$_6$, 25 °C) δ [ppm] = 11.10 (bs, 1 H, amide-N*H*1), 10.16 (bs, 1 H, amide-N*H*2) 7.25 (m, 5 H, H7-11), 6.37 (s, 1 H, H3), 4.16 (t, 1 H, $^{3}J_{H,H}$ = 6.1 Hz, N*H*4), 3.05 (q, 2 H, $^{3}J_{H,H}$ = 6.8 Hz, H5), 2.82 (t, 2 H, $^{3}J_{H,H}$ = 7.3 Hz, H6).

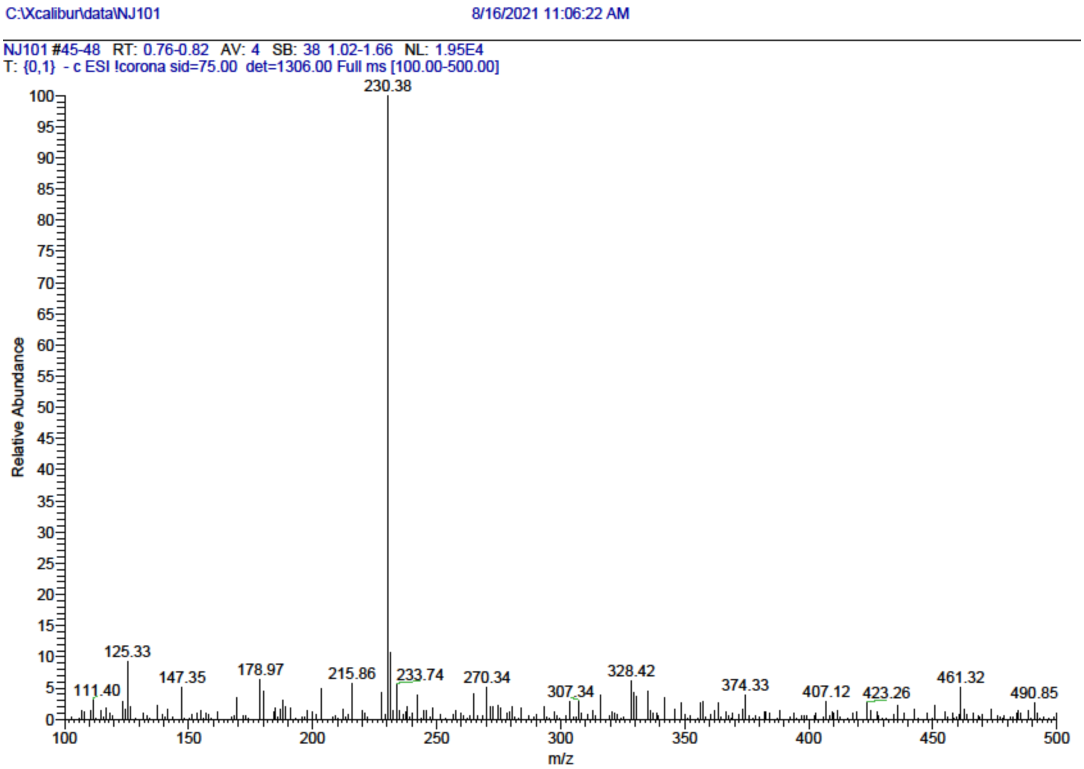

MS (ESI$^-$): *m/z* (%) = 230.38 [M-H]$^-$.

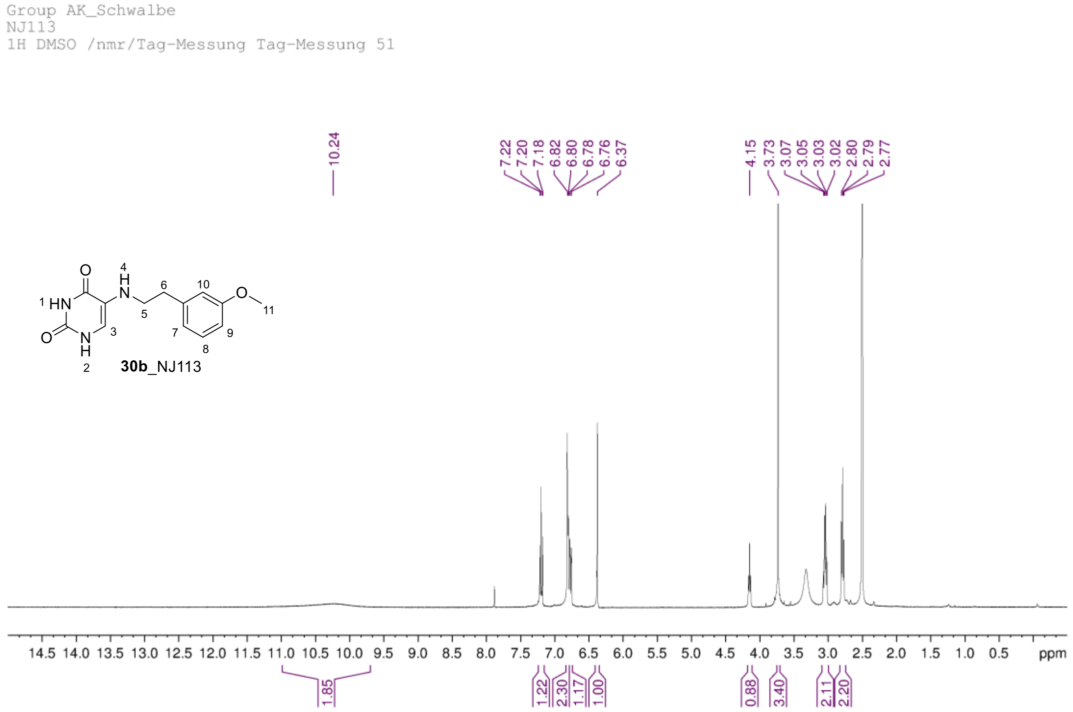

^{1}H-NMR (400.26 MHz, DMSO-*d*6, 25 °C) δ [ppm] = 10.24 (bs, 2 H, amide-N*H*1/2), 7.20 (t, 1 H, $^3J_{H,H}$ = 8.0 Hz, H8), 6.82-6.80 (m, 2 H, H7 & H9), 6.76 (dd, 1 H, $^3J_{H,H}$ = 8.5 Hz, $^4J_{H,H}$ = 2.6 Hz, H10), 6.37 (s, 1 H, H3), 4.15 (t, 1 H, $^3J_{H,H}$ = 6.2 Hz, N*H*4), 3.73 (s, 3 H, C*H*311), 3.04 (q, 2 H, $^3J_{H,H}$ = 6.8 Hz, H5), 2.79 (t, 2 H, $^3J_{H,H}$ = 6.8 Hz, H6).

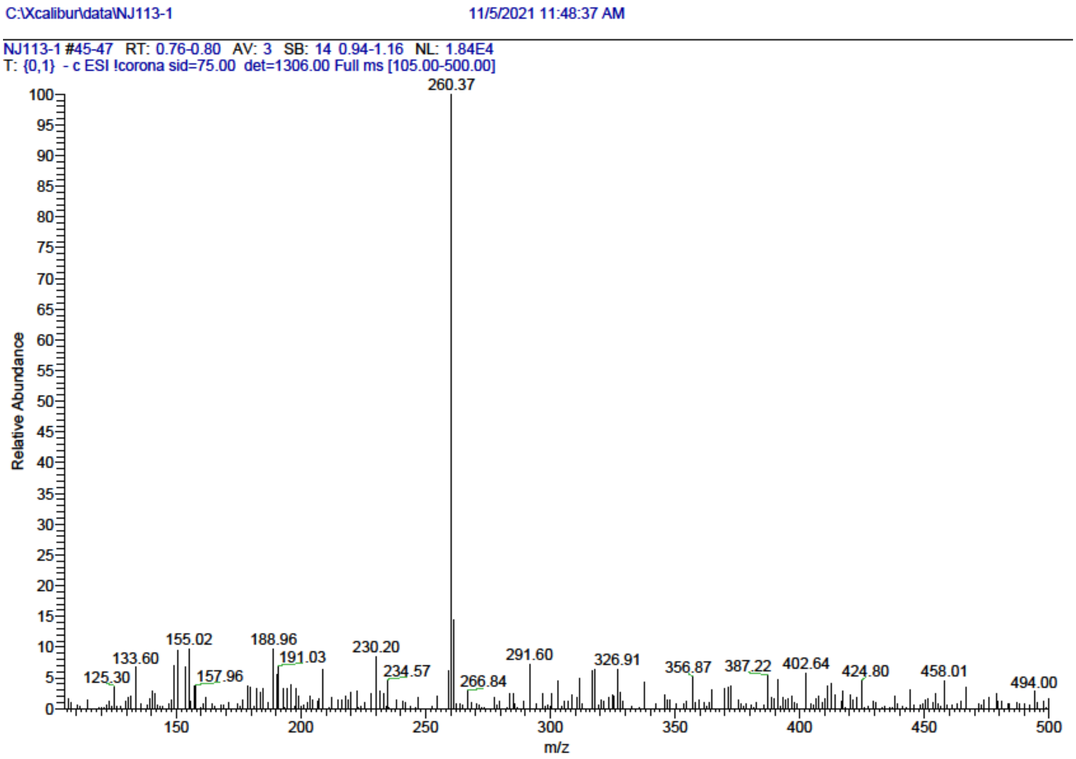

MS (ESI$^-$): *m/z* (%) = 260.37 [M-H]$^-$.

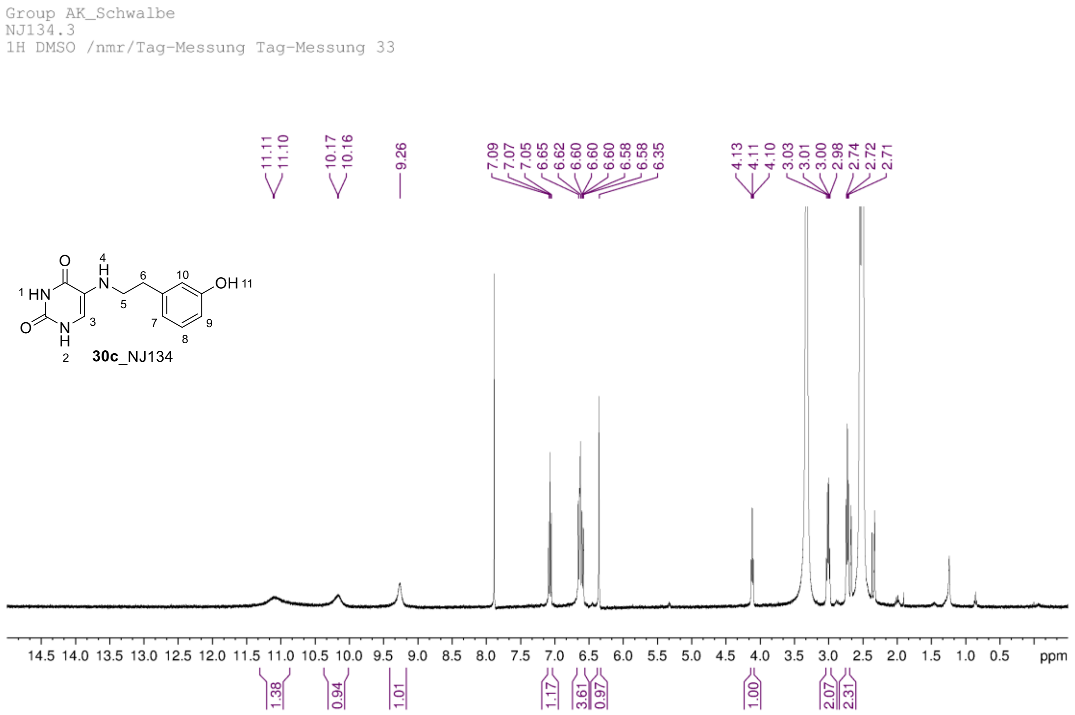

^{1}H-NMR (400.26 MHz, DMSO-d_6, 25 °C) δ [ppm] = 11.11 (bs, 1 H, amide-N*H*1), 10.17 (bs, 1 H, amide-N*H*2), 9.26 (bs, 1 H, *OH*11), 7.07 (t, 1 H, $^3J_{H,H}$ = 7.6 Hz, H8), 6.66-6.58 (m, 4 H, H7, H9, H10), 6.35 (s, 1 H, H3), 4.11 (t, 1 H, $^3J_{H,H}$ = 6.2 Hz, N*H*4), 3.00 (q, 2 H, $^3J_{H,H}$ = 6.7 Hz, H5), 2.72 (t, 2 H, $^3J_{H,H}$ = 6.7 Hz, H6).

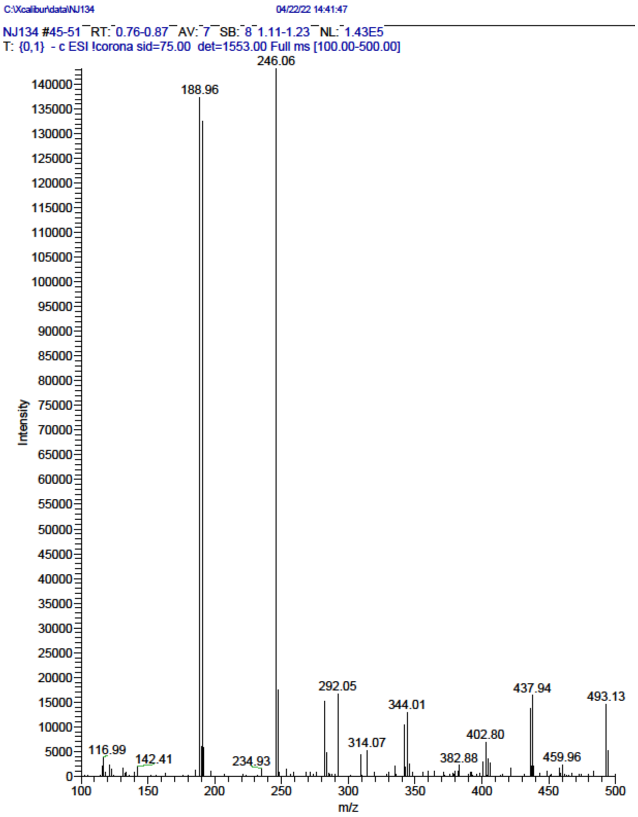

MS (ESI$^-$): *m/z* (%) = 246.06 [M-H]$^-$.

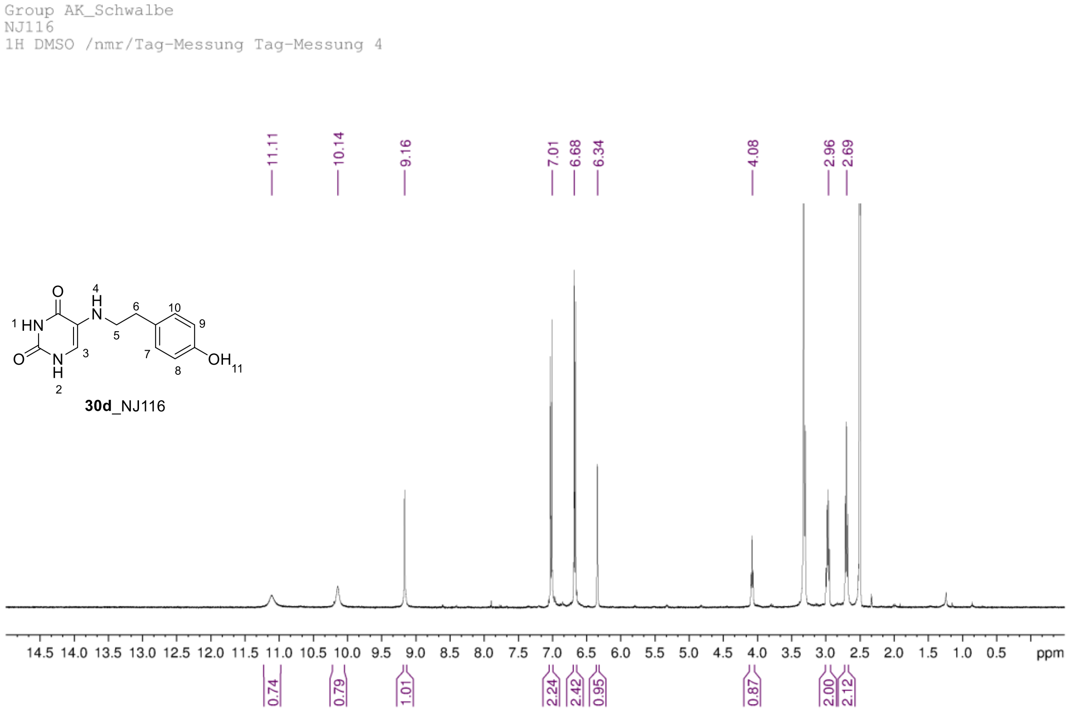

^{1}**H-NMR** (400.26 MHz, DMSO-d_6, 25 °C) δ [ppm] = 11.11 (bs, 1 H, amide-N*H*1), 10.14 (bs, 1 H, amide-N*H*2), 9.16 (s, 1 H, *OH*11), 7.01 (d, 2 H, $^{3}J_{H,H}$ = 8.4 Hz, H7 & H10), 6.68 (d, 2 H, $^{3}J_{H,H}$ = 8.4 Hz, H8 & H9), 6.34 (s, 1 H, H3), 4.08 (t, 1 H, $^{3}J_{H,H}$ = 6.1 Hz, N*H*4), 2.97 (q, 2 H, $^{3}J_{H,H}$ = 6.5 Hz, H5), 2.69 (t, 2 H, $^{3}J_{H,H}$ = 7.4 Hz, H6).

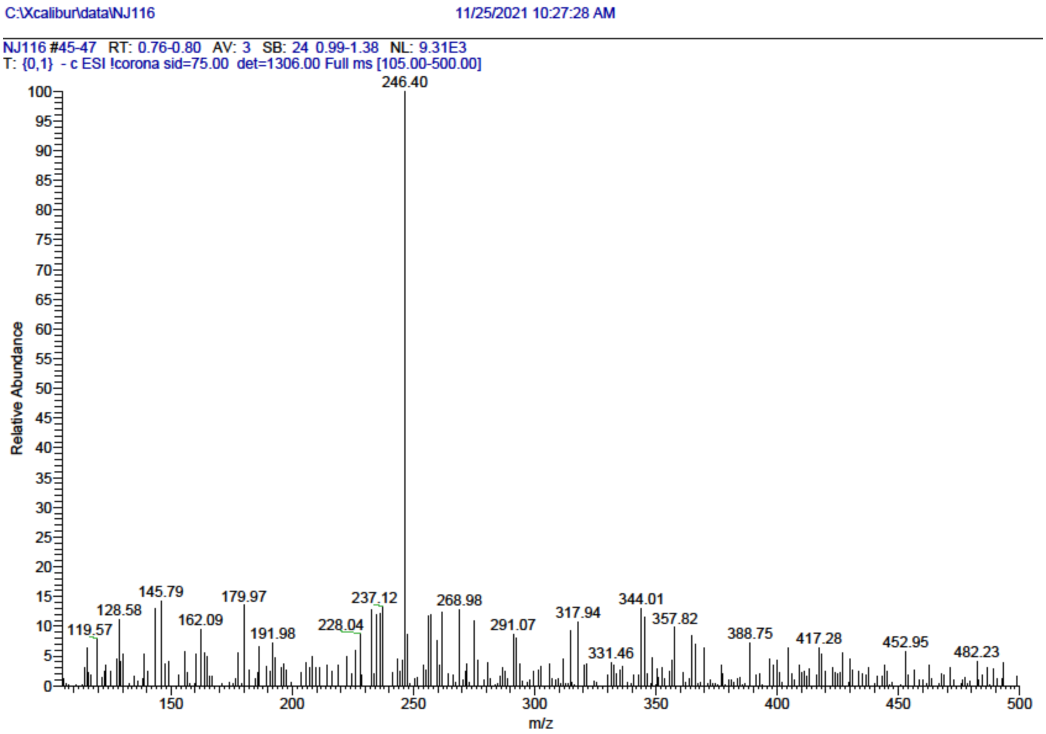

MS (ESI$^-$): *m/z* (%) = 246.40 [M-H]$^-$.

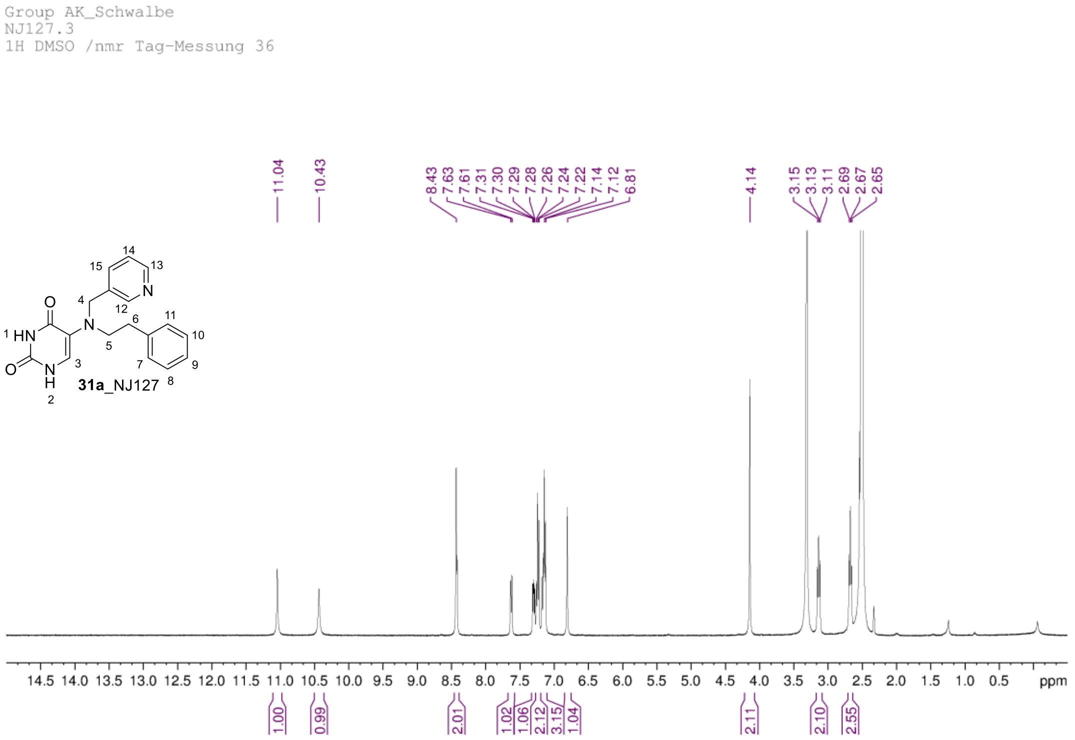

^{1}H-NMR (400.26 MHz, DMSO-d_6, 25 °C) δ [ppm] = 11.04 (bs, 1 H, amide-N*H*1), 10.43 (bs, 1 H, amide-N*H*2), 8.43-8.41 (m, 2 H, H13 & H12), 7.62 (d, 2 H, $^{3}J_{H,H}$ = 8.0 Hz, H14), 7.30 (dd, 1 H, $^{3}J_{H,H}$ = 7.7 Hz, $^{4}J_{H,H}$ = 4.7 Hz, H15), 7.26-7.22 (m, 2 H, H8 & H10), 7.17-7.12 (m, 3 H, H7, H9, H11), 6.81 (s, 1 H, H3) 4.14 (s, 2 H, H4), 3.14 (t, 2 H, $^{3}J_{H,H}$ = 7.8 Hz, H5), 2.67 (t, 2 H, $^{3}J_{H,H}$ = 7.1 Hz, H6).

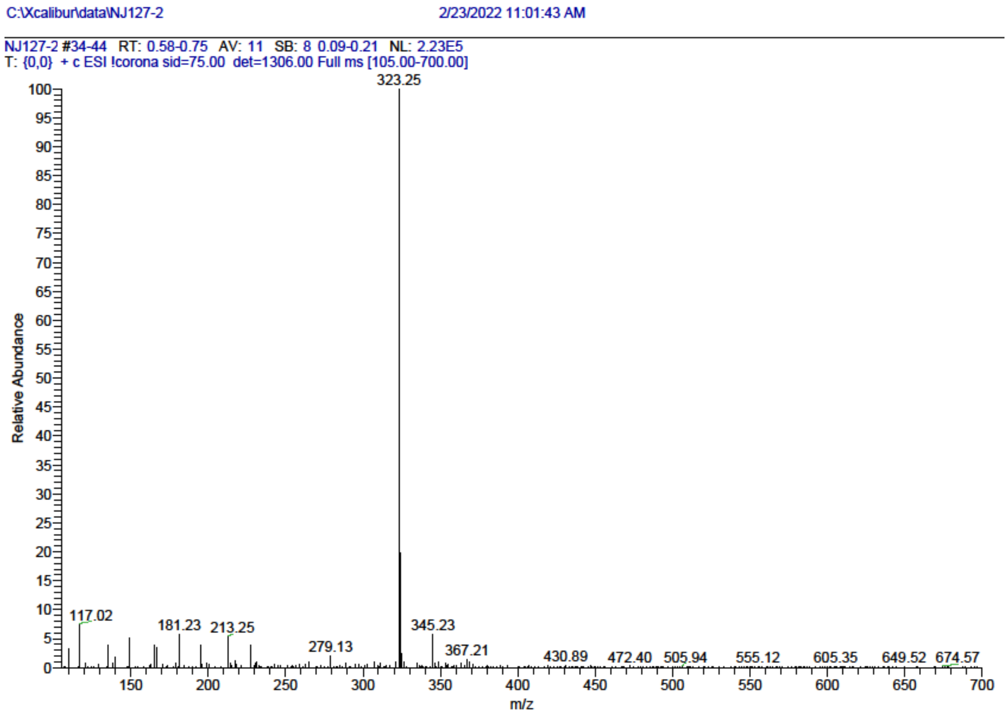

MS (ESI$^-$): *m/z* (%) = 323.25 [M-H]$^-$.

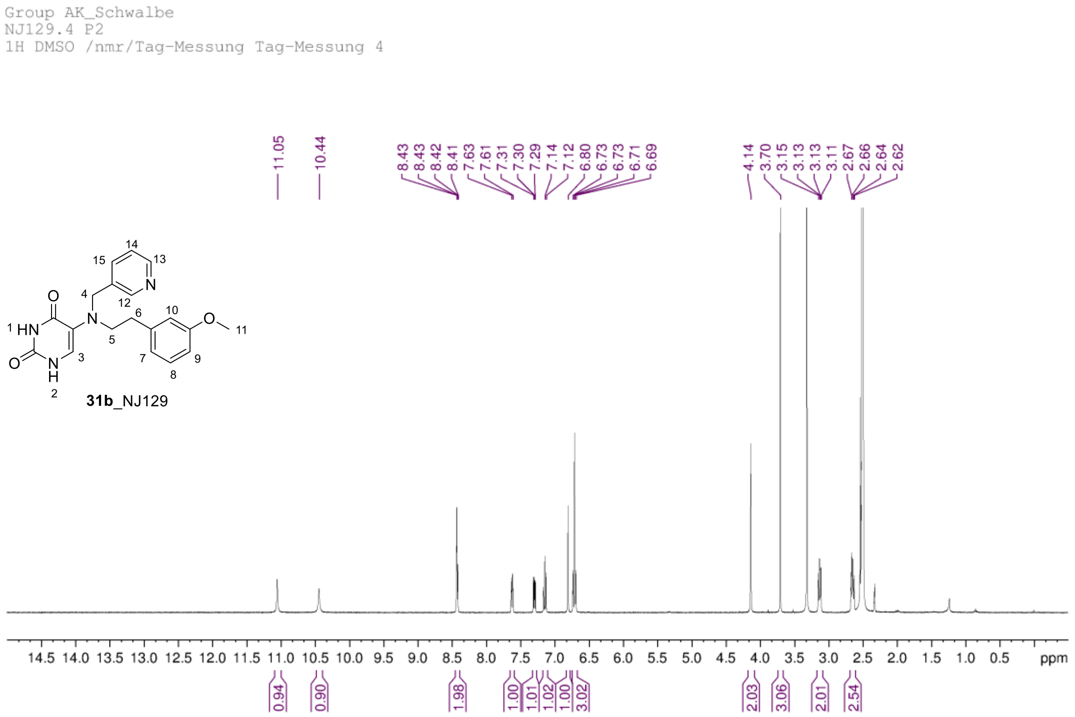

^{1}H-NMR (400.26 MHz, DMSO-*d*$_6$, 25 °C) δ [ppm] = 11.05 (bs, 1 H, amide-N*H*1), 10.44 (bs, 1 H, amide-N*H*2), 8.43-8.41 (m, 2 H, H13 & H12), 7.62 (dt, 2 H, $^3J_{H,H}$ = 8.0 Hz, $^4J_{H,H}$ = 2.0 Hz, H14), 7.30 (dd, 1 H, $^3J_{H,H}$ = 7.7 Hz, $^4J_{H,H}$ = 4.7 Hz, H15), 7.14 (t, $^3J_{H,H}$ = 7.9 Hz, 1 H, H8), 6.80 (s, 1 H, H10), 6.74-6.69 (m, 3 H, H3, H7, H9) 4.14 (s, 2 H, H4), 3.70 (s, 3 H, C*H*$_3$11), 3.13 (t, 2 H, $^3J_{H,H}$ = 8.3 Hz, H5), 2.64 (t, 2 H, $^3J_{H,H}$ = 7.7 Hz, H6).

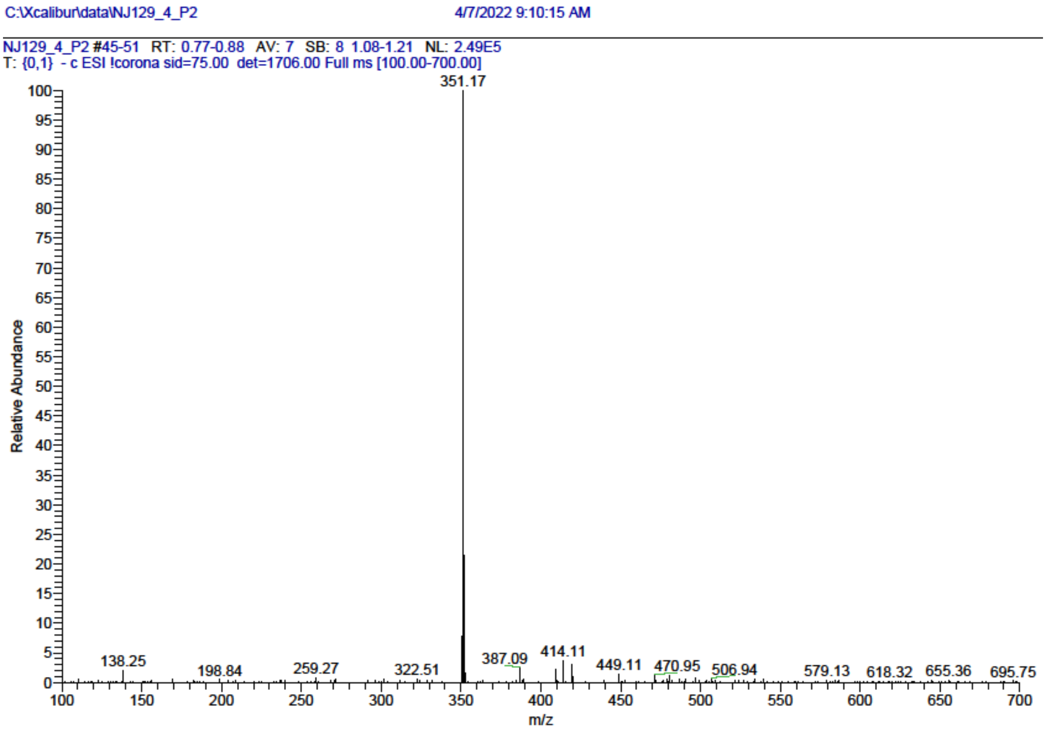

MS (ESI$^-$): *m/z* (%) = 351.17 [M-H]$^-$.

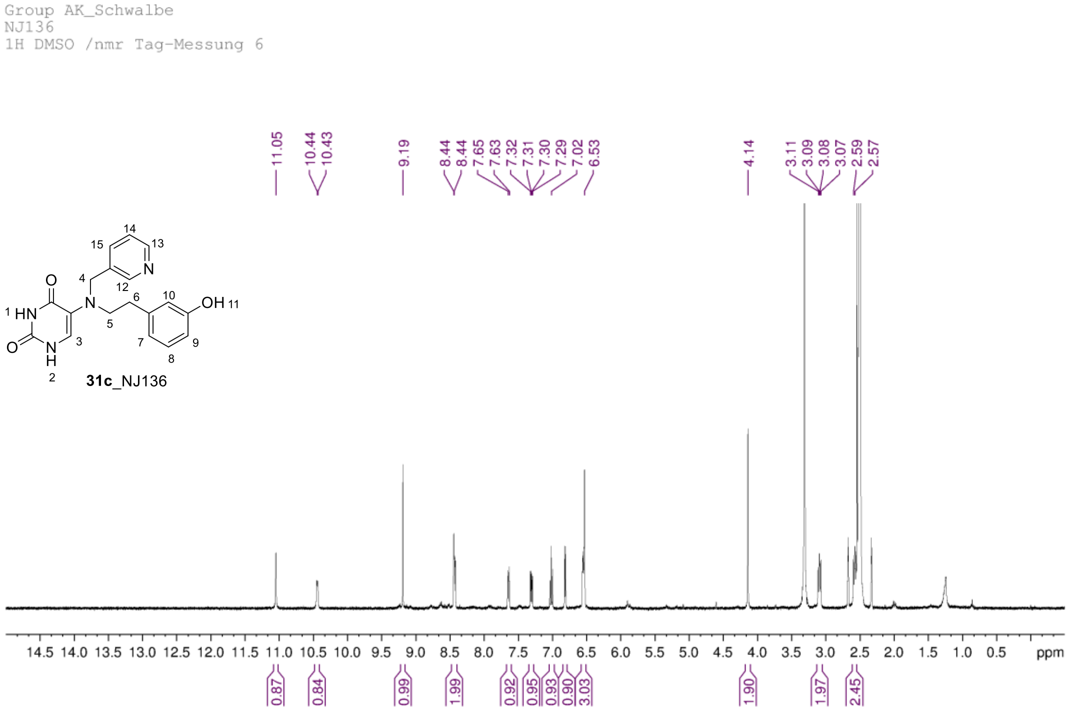

^{1}H-NMR (400.26 MHz, DMSO-*d*$_6$, 25 °C) δ [ppm] = 11.05 (bs, 1 H, amide-N*H*1), 10.44 (bs, 1 H, amide-N*H*2), 9.19 (s, 1 H, *OH*11), *8*.44-8.42 (m, 2 H, H13 & H12), 7.64 (dt, 2 H, $^3J_{H,H}$ = 7.8 Hz, $^4J_{H,H}$ = 1.8 Hz, H14), 7.31 (dd, 1 H, $^3J_{H,H}$ = 7.9 Hz, $^4J_{H,H}$ = 4.9 Hz, H15), 7.02 (t, $^3J_{H,H}$ = 7.7 Hz, 1 H, H8), 6.80 (d, 1 H, $^4J_{H,H}$ = 5.0 Hz, H10), 6.56-6.53 (m, 3 H, H3, H7, H9) 4.14 (s, 2 H, H4), 3.09 (t, 2 H, $^3J_{H,H}$ = 7.5 Hz, H5), 2.60 (t, 2 H, $^3J_{H,H}$ = 7.9 Hz, H6).

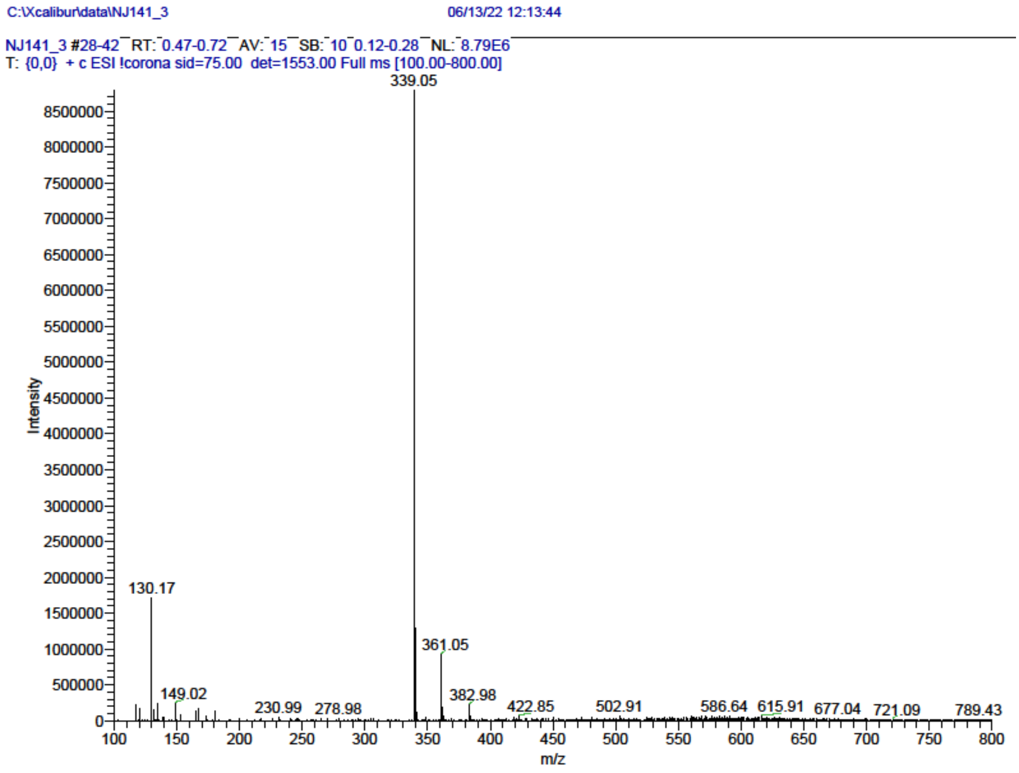

MS (ESI$^+$): *m/z* (%) = 339.05 [M+H]$^+$.

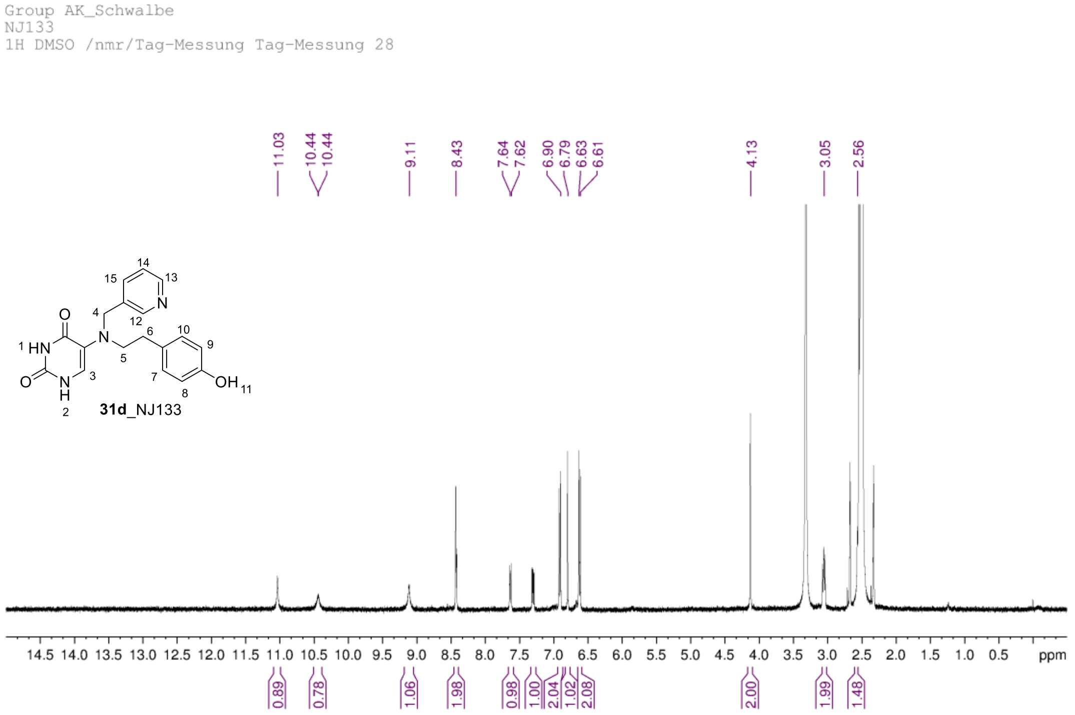

^{1}**H-NMR** (400.26 MHz, DMSO-*d*$_6$, 25 °C) δ [ppm] = 11.03 (bs, 1 H, amide-N*H*1), 10.44 (bs, 1 H, amide-N*H*2), 9.19 (bs, 1 H, *OH*11), *8*.43-8.41 (m, 2 H, H13 & H12), 7.63 (dt, 2 H, $^3J_{H,H}$ = 7.8 Hz, $^4J_{H,H}$ = 1.8 Hz, H14), 7.30 (dd, 1 H, $^3J_{H,H}$ = 7.9 Hz, $^4J_{H,H}$ = 4.9 Hz, H15), 6.91 (d, $^3J_{H,H}$ = 8.2 Hz, 2 H, H7 & H10), 6.79 (s, 1 H, H3), 6.62 (d, 2 H, H8 &, H9) 4.13 (s, 2 H, H4), 3.05 (t, 2 H, $^3J_{H,H}$ = 8.0 Hz, H5), 2.60 (t, 2 H, $^3J_{H,H}$ = 7.9 Hz, H6).

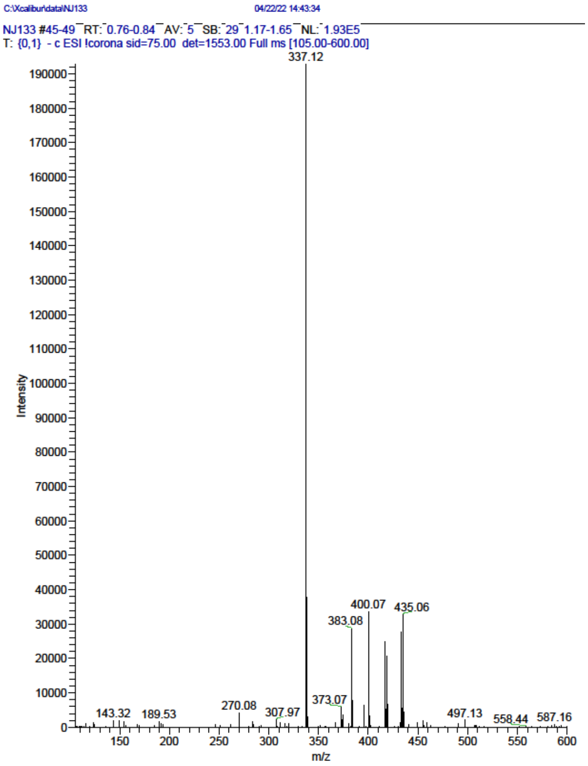

MS (ESI$^-$): *m/z* (%) = 337.12 [M-H]$^-$.

Analytic of peptide-like tertiary structured ligands based on the scaffold of Z604

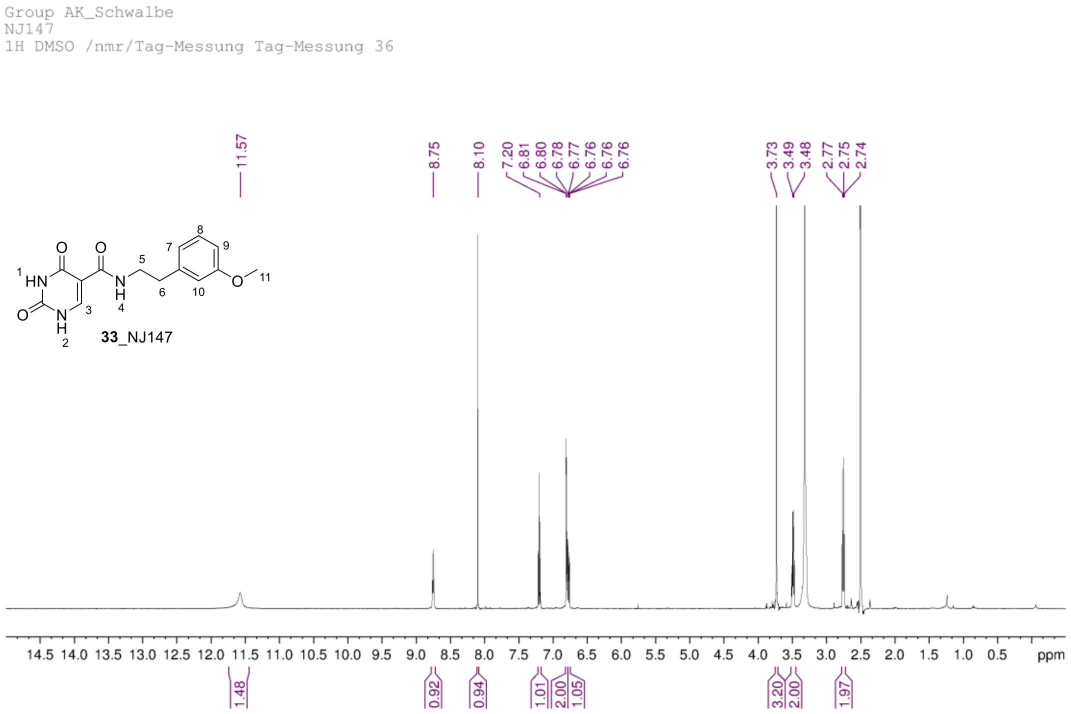

^{1}H-NMR (500.18 MHz, DMSO-*d*$_6$, 25 °C) δ [ppm] = 11.57 (bs, 2 H, amide-N*H*1 & amide-N*H*2), 8.75 (t,

$^{3}J_{H,H}$ = 6.1 Hz, 1 H, amide-N*H*4), 8.10 (s, 1 H, H3), 7.20 (t, 1 H, $^{3}J_{H,H}$ = 8.1 Hz, H8), 6.80-6.79 (m, 2 H, H7 & H9), 6.78-6.76 (m, 1 H, H10), 3.73 (s, 3 H, C*H*3 11), 3.49 (q, 2 H, $^{3}J_{H,H}$ = 6.1 Hz, H5), 2.75 (t, 2 H, $^{3}J_{H,H}$ = 7.1 Hz, H6).

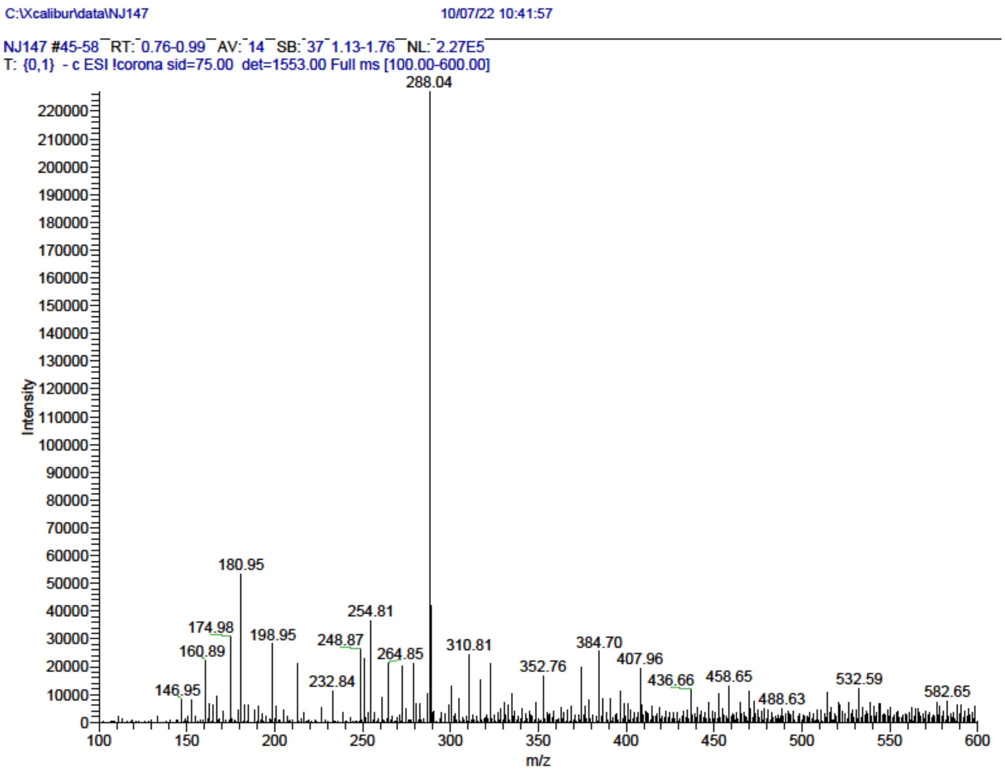

MS (ESI⁻): *m/z* (%) = 288.04 [M-H]⁻.

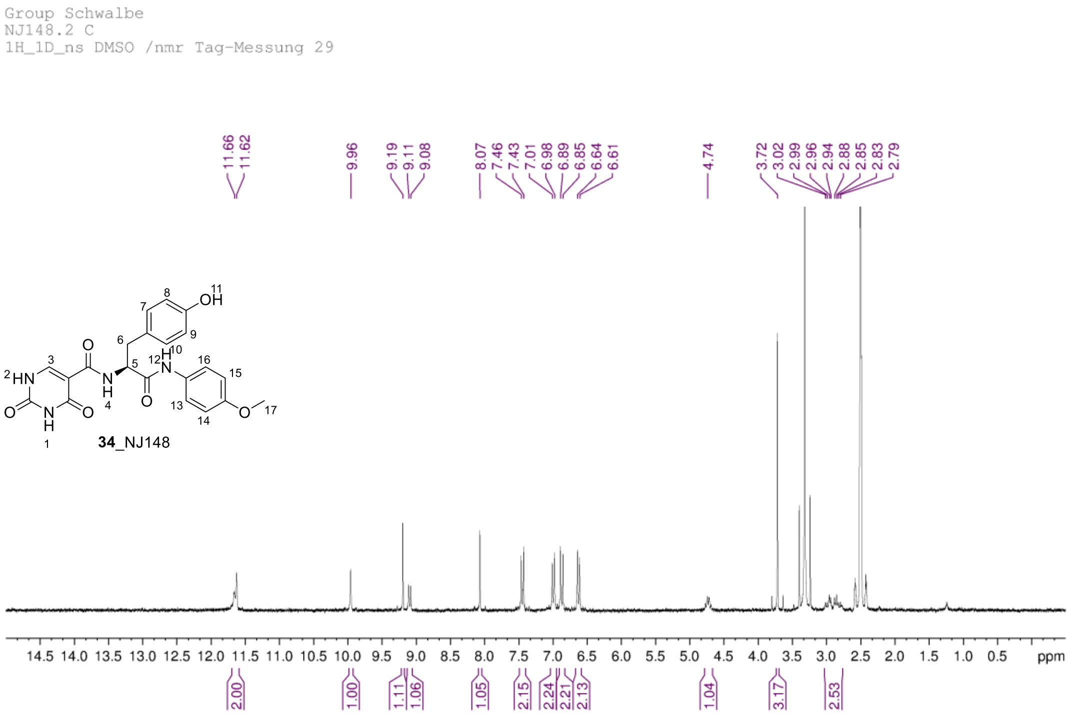

^{1}H-NMR (500.18 MHz, DMSO-d_6, 25 °C) δ [ppm] = 11.66-11.62 (m, 2 H, amide-N*H*1 & amide-N*H*2), 9.96 (s, 1 H, amide-N*H*10), 9.19 (s, 1 H, *OH*11), 9.10 (d, $^3J_{H,H}$ = 7.8 Hz, 1 H, amide-N*H*4), 8.07 (s, 1 H, H3), 7.45 (d, 2 H, $^3J_{H,H}$ = 9.0 Hz, H13 & H16), 7.00 (d, 2 H, $^3J_{H,H}$ = 8.3 Hz, H7 & H10), 6.87 (d, $^3J_{H,H}$ = 9.1 Hz, 2 H, H14 & H15), 6.63 (d, 2 H, $^3J_{H,H}$ = 8.4 Hz, H8 & H9), 4.77-4.69 (m, 1 H, H5), 3.72 (s, 3 H, C*H*$_3$17), 3.02-2.78 (m, 2 H, H6).

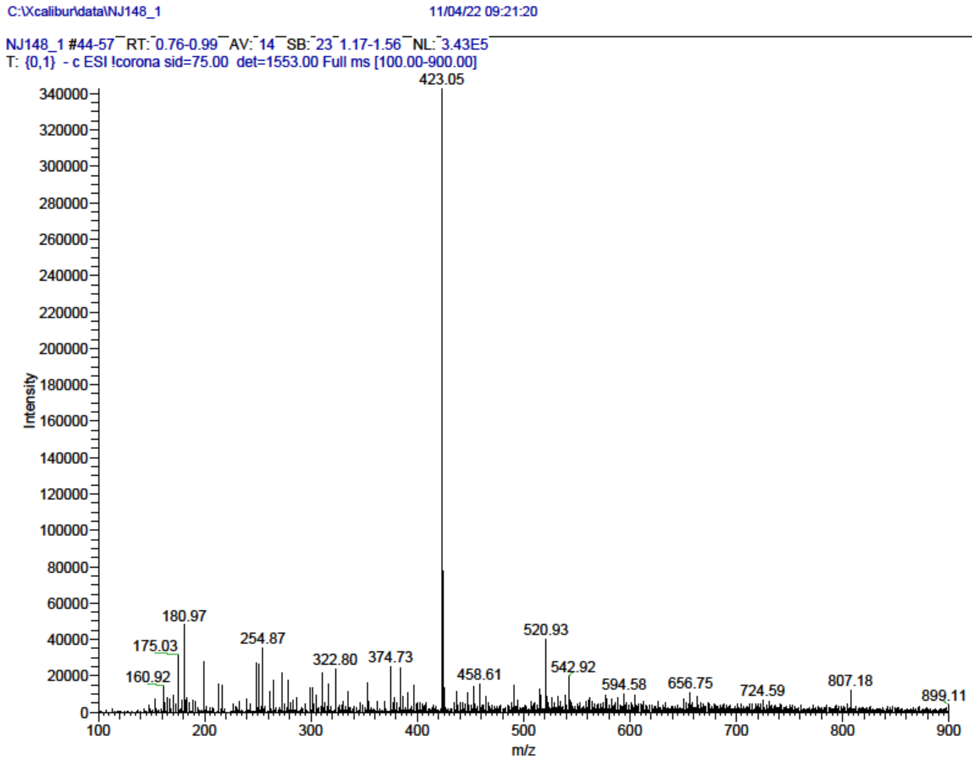

MS (ESI$^-$): *m/z* (%) = 423.05 [M-H]$^-$.

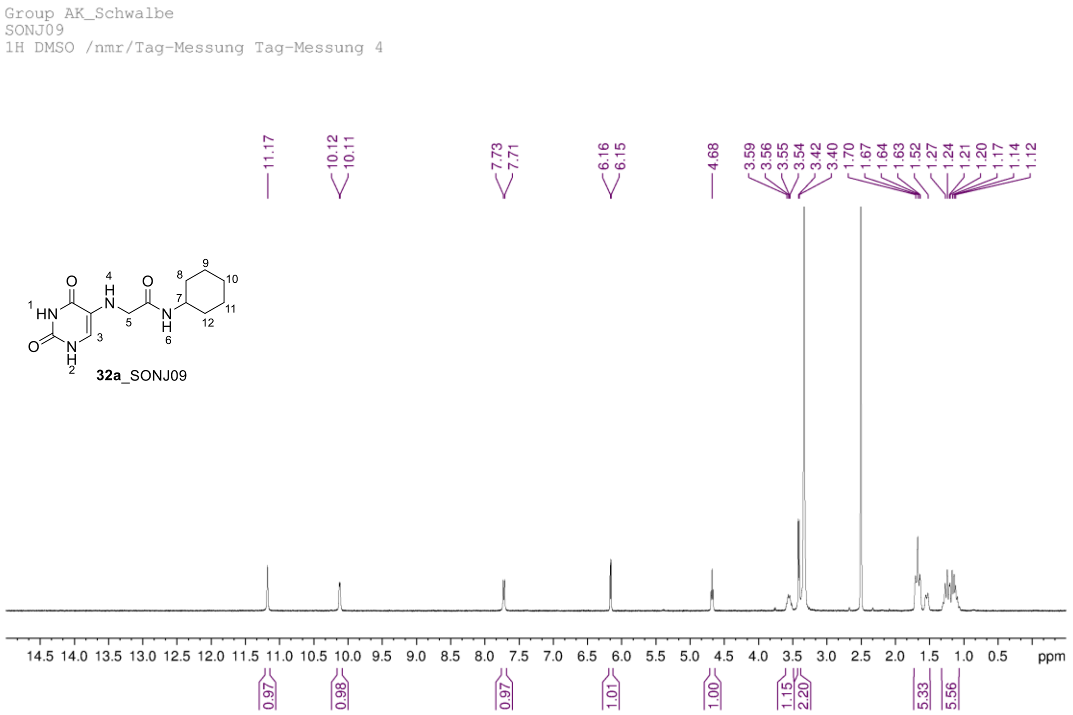

^{1}H-NMR (400.26 MHz, DMSO-d_6, 25 °C) δ [ppm] = 11.17 (s, 1 H, amide-N*H*2), 10.12 (d, $^4J_{H,H}$ = 5.0 Hz, 1 H, amide-N*H*1), 7.72 (d, $^3J_{H,H}$ = 8.4 Hz, 1 H, N*H*6), 6.16 (d, $^3J_{H,H}$ = 5.9 Hz, 1 H, H3), 4.68 (t, $^3J_{H,H}$ = 5.9 Hz, 2 H, N*H*4), 3.59-3.52 (m, 1 H, H7), 3.41 (d, $^3J_{H,H}$ = 5.9 Hz, 2 H, H5), 1.70-1.52 (m, 5 H, H8-12), 1.29-1.06 (m, 5 H, H8-12).

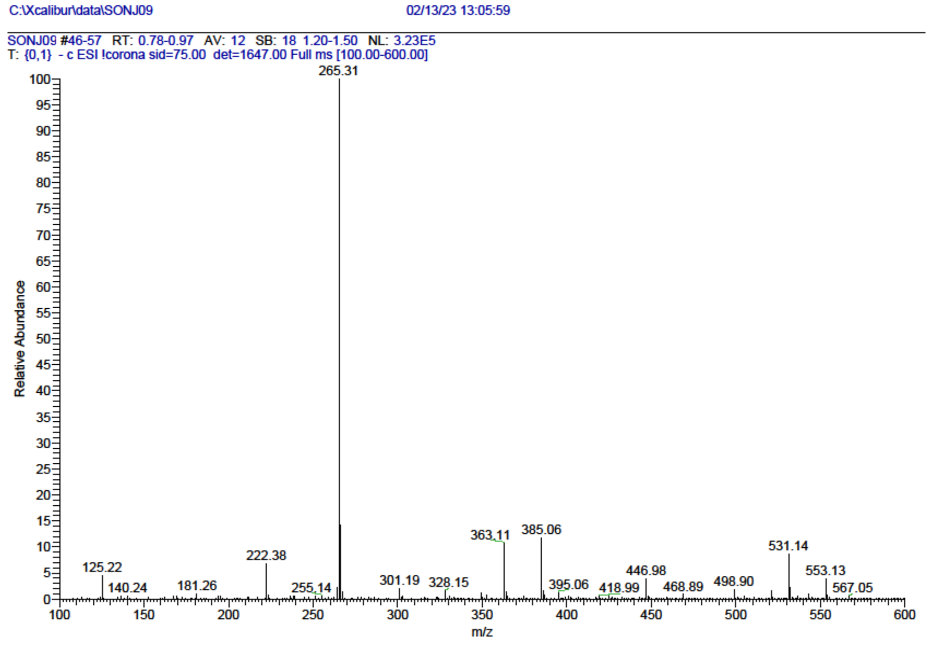

MS (ESI$^-$): *m/z* (%) = 265.31 [M-H]$^-$.

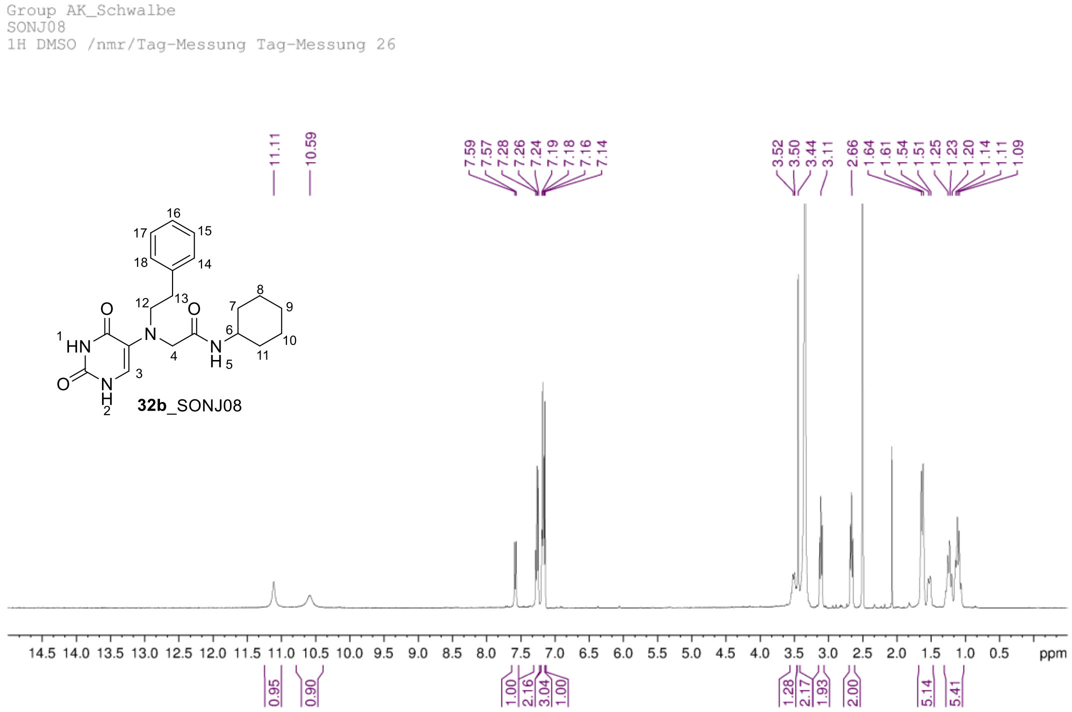

^{1}**H-NMR** (400.26 MHz, DMSO-d_6, 25 °C) δ [ppm] = 11.11 (bs, 1 H, amide-N*H*2), 10.59 (bs, 1 H, amide-N*H*1), 7.58 (d, $^{3}J_{H,H}$ = 8.2 Hz, 1 H, N*H*5), 7.82-7.16 (m, 5 H, H14-H18), 7.15 (s, 1 H, H3), 3.54-3.47 (m, 1 H, H6), 3.44 (s, 2 H, H4), 3.11 (t, $^{3}J_{H,H}$ = 8.2 Hz, 1 H, H12), 2.66 (t, $^{3}J_{H,H}$ = 8.2 Hz, 1 H, H13), 1.64-1.51 (m, 5 H, H7-11), 1.29-1.06 (m, 5 H, H7-11).

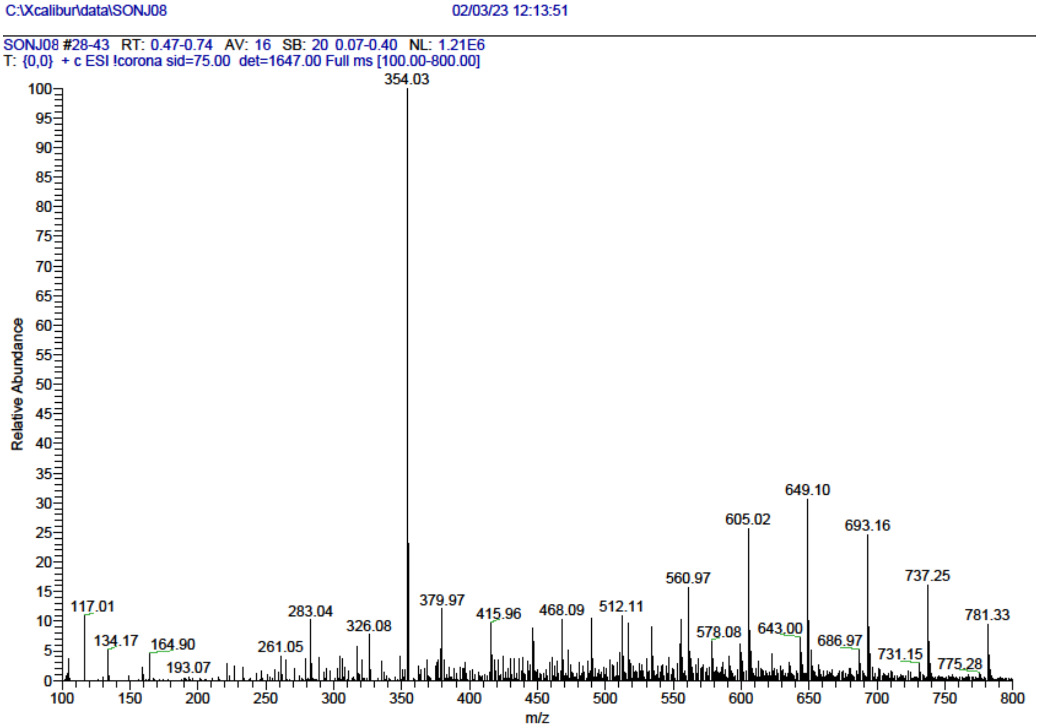

MS (ESI^{-}): *m/z* (%) = 354.03 [M-O]$^{-}$.

Analytic of X77-like compounds **35a-b** containing Uracil moiety

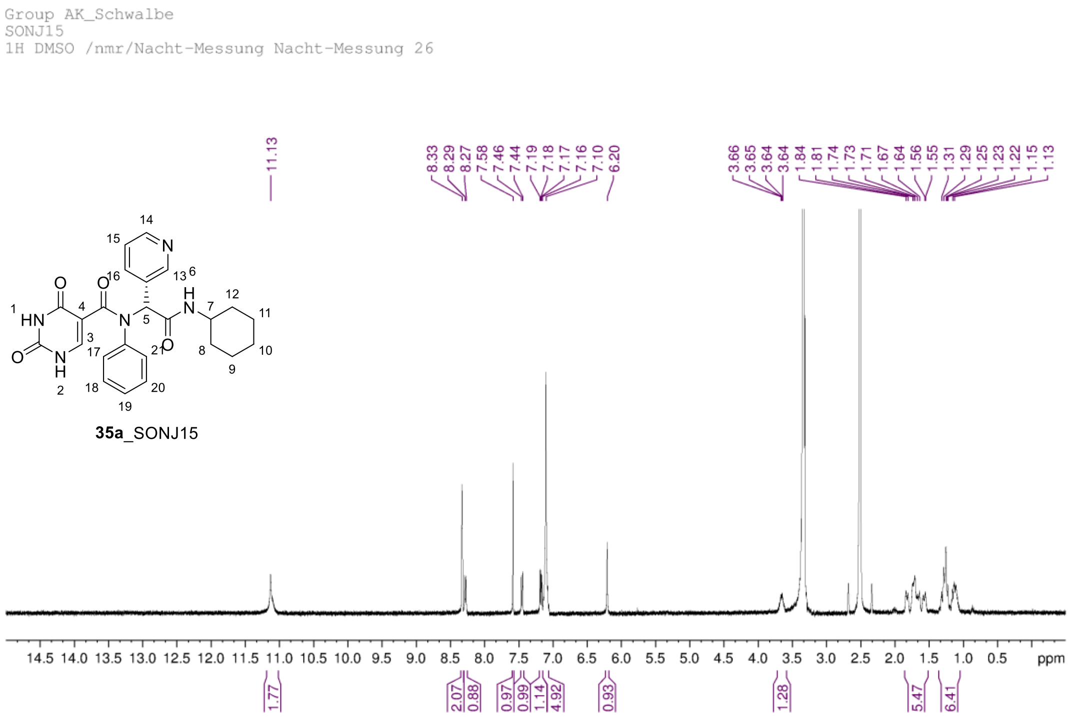

^{1}H-NMR (400.26 MHz, DMSO-d_6, 25 °C) δ [ppm] = 11.13 (bs, 2 H, amide-N*H*1&2), 8.33-8.32 (m, 2 H, H13 & H14), 8.28 (d, $^3J_{H,H}$ = 7.9 Hz, 1 H, amide-N*H*6), 7.58 (s, 1 H, H3), 7.45 (d, $^3J_{H,H}$ = 8.1 Hz, 1 H, H15), 7.17 (dd, 1 H, $^3J_{H,H}$ = 8.0 Hz, $^4J_{H,H}$ = 4.8 Hz, H16), 7.10 (s, 5 H, H17-21), 6.20 (s, 1 H, H5), 3.71-3.61 (m, 1 H, H7), 1.83-1.55 (m, 5 H, H8-12), 1.31-1.08 (m, 5 H, H8-12).

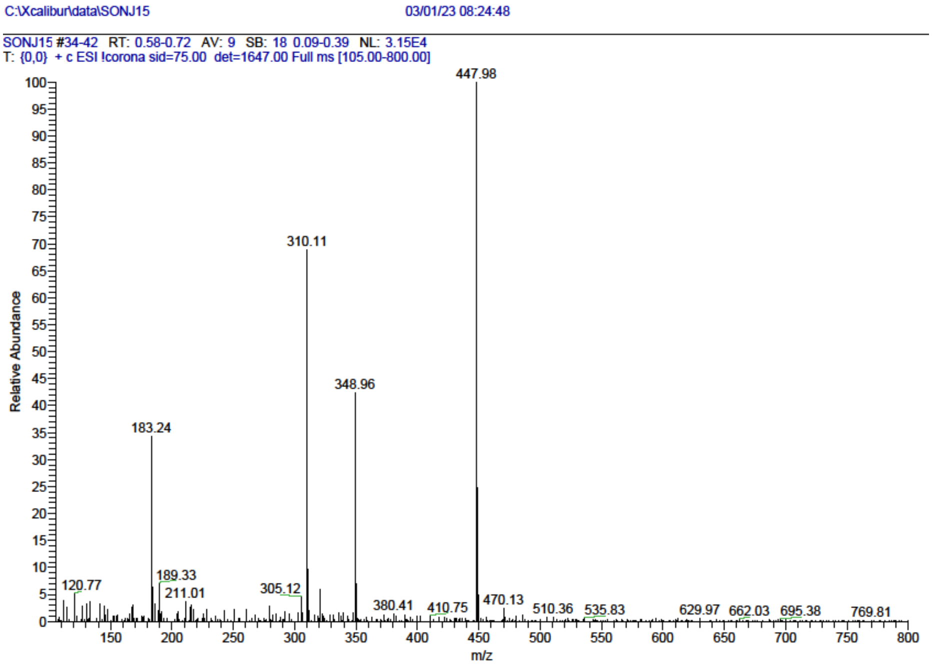

MS (ESI$^+$): *m/z* (%) = 447.98 [M+H]$^+$.

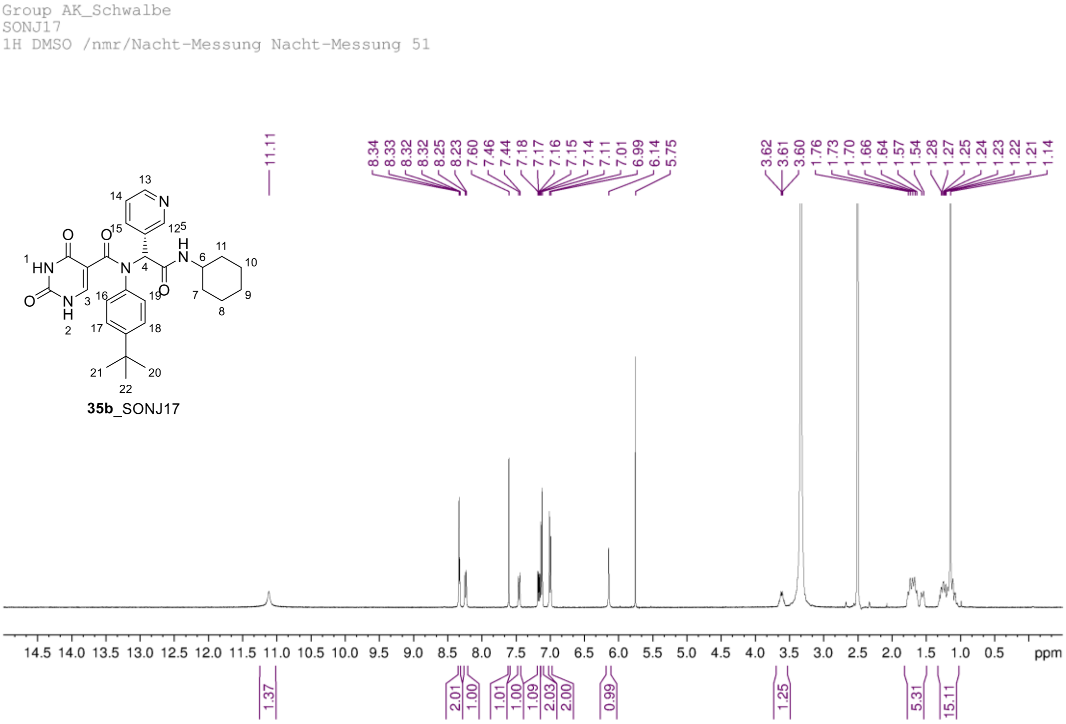

^{1}H-NMR (400.26 MHz, DMSO-d_6, 25 °C) δ [ppm] = 11.11 (bs, 2 H, amide-N*H*1&2), 8.34-8.32 (m, 2 H, H12 & H13), 8.24 (d, $^{3}J_{H,H}$ = 7.9 Hz, 1 H, amide-N*H*6), 7.60 (s, 1 H, H3), 7.45 (d, $^{3}J_{H,H}$ = 8.0 Hz, 1 H, H14), 7.16 (dd, 1 H, $^{3}J_{H,H}$ = 8.0 Hz, $^{4}J_{H,H}$ = 3.2 Hz, H15), 7.12 (d, 2 H, $^{3}J_{H,H}$ = 8.6 Hz, H16 & H19), 7.00 (d, 2 H, $^{3}J_{H,H}$ = 8.6 Hz, H17 & H18), 6.14 (s, 1 H, H4), 3.66-3.57 (m, 1 H, H6), 1.76-1.54 (m, 5 H, H7-11), 1.30-1.06 (m, 5 H, H7-11), 1.14 (s, 9 H, tBu).

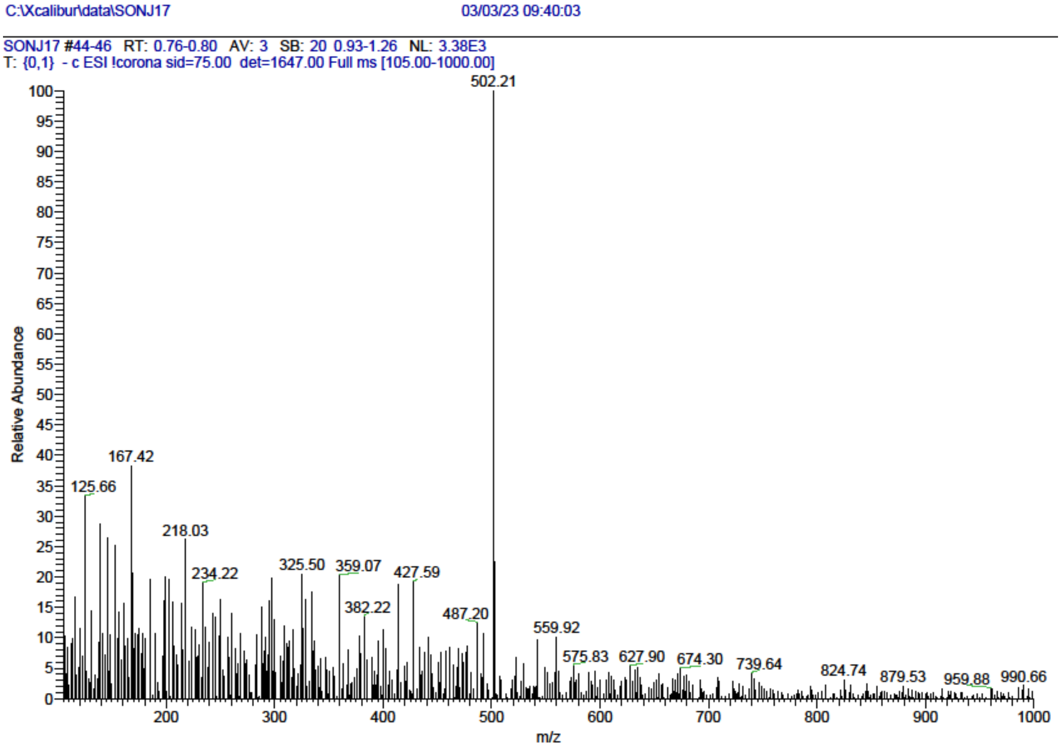

MS (ESI^{-}): *m/z* (%) = 502.21 [M-H]$^{-}$.

References

1. Berg, H.; Wirtz Martin, M. A.; Altincekic, N.; Alshamleh, I.; Kaur Bains, J.; Blechar, J.*; et al.*, Comprehensive Fragment Screening of the SARS-CoV-2 Proteome Explores Novel Chemical Space for Drug Development. *Angewandte Chemie International Edition* **2022,** *61* (46), e202205858.

2. Vulpetti, A.; Dalvit, C., Design and generation of highly diverse fluorinated fragment libraries and their efficient screening with improved (19) F NMR methodology. *ChemMedChem* **2013,** *8* (12), 2057-69.

3. Zhang, L.; Lin, D.; Sun, X.; Curth, U.; Drosten, C.; Sauerhering, L.; Becker, S.; Rox, K.; Hilgenfeld, R., Crystal structure of SARS-CoV-2 main protease provides a basis for design of improved α-ketoamide inhibitors. *Science* **2020,** *368* (6489), 409-412.

4. Studier, F. W., Protein production by auto-induction in high density shaking cultures. *Protein expression and purification* **2005,** *41* (1), 207-34.

5. Mueller, U.; Förster, R.; Hellmig, M.; Huschmann, F. U.; Kastner, A.; Malecki, P.*; et al.*, The macromolecular crystallography beamlines at BESSY II of the Helmholtz-Zentrum Berlin: Current status and perspectives. *The European Physical Journal Plus* **2015,** *130* (7), 141.

6. Sparta, K. M.; Krug, M.; Heinemann, U.; Mueller, U.; Weiss, M. S., XDSAPP2.0. *Journal of Applied Crystallography* **2016,** *49* (3), 1085-1092.

7. Schiebel, J.; Krimmer, S. G.; Röwer, K.; Knörlein, A.; Wang, X.; Park, A. Y.*; et al.*, High-Throughput Crystallography: Reliable and Efficient Identification of Fragment Hits. *Structure (London, England : 1993)* **2016,** *24* (8), 1398-1409.

8. Emsley, P.; Lohkamp, B.; Scott, W. G.; Cowtan, K., Features and development of Coot. *Acta crystallographica. Section D, Biological crystallography* **2010,** *66* (Pt 4), 486-501.

9. Liebschner, D.; Afonine, P. V.; Baker, M. L.; Bunkóczi, G.; Chen, V. B.; Croll, T. I.*; et al.*, Macromolecular structure determination using X-rays, neutrons and electrons: recent developments in Phenix. *Acta crystallographica. Section D, Structural biology* **2019,** *75* (Pt 10), 861-877.

10. Grosdidier, A.; Zoete, V.; Michielin, O., Fast docking using the CHARMM force field with EADock DSS. *Journal of computational chemistry* **2011,** *32* (10), 2149-59.

11. Grosdidier, A.; Zoete, V.; Michielin, O., SwissDock, a protein-small molecule docking web service based on EADock DSS. *Nucleic acids research* **2011,** *39* (Web Server issue), W270-7.

12. Mulder, F. A. A.; Schipper, D.; Bott, R.; Boelens, R., Altered flexibility in the substrate-binding site of related native and engineered high-alkaline Bacillus subtilisins11Edited by P. E. Wright. *Journal of Molecular Biology* **1999,** *292* (1), 111-123.

13. Milligan, J. C.; Zeisner, T. U.; Papageorgiou, G.; Joshi, D.; Soudy, C.; Ulferts, R.*; et al.*, Identifying SARS-CoV-2 antiviral compounds by screening for small molecule inhibitors of Nsp5 main protease. *Biochemical Journal* **2021,** *478* (13), 2499-2515.

14. Andrianov, A. M.; Kornoushenko, Y. V.; Karpenko, A. D.; Bosko, I. P.; Tuzikov, A. V., Computational discovery of small drug-like compounds as potential inhibitors of SARS-CoV-2 main protease. *Journal of Biomolecular Structure and Dynamics* **2021,** *39* (15), 5779-5791.

15. Kneller, D. W.; Li, H.; Galanie, S.; Phillips, G.; Labbé, A.; Weiss, K. L.*; et al.*, Structural, Electronic, and Electrostatic Determinants for Inhibitor Binding to Subsites S1 and S2 in SARS-CoV-2 Main Protease. *Journal of Medicinal Chemistry* **2021,** *64* (23), 17366-17383.

16. Phillips, A. P., Some 5-Substituted Aminouracils. *Journal of the American Chemical Society* **1951,** *73* (3), 1061-1062.

17. Gaulon, C.; Dijkstra, H. P.; Springer, C. J., A General and Facile Route to New Trisubstituted Purin-8-ones. *Synthesis* **2005,** *2005* (13), 2227-2233.

18. Moore, J. L.; Taylor, S. M.; Soloshonok, V. A., An efficient and operationally convenient general synthesis of tertiary amines by direct alkylation of secondary amines with alkyl halides in the presence of Huenig's base. *ARKIVOC* **2005,** *2005* (6), 287-292.
